# Molecular Diagnosis of Chagas Disease in Colombia: Parasitic Loads and Discrete Typing Units in Patients from Acute and Chronic Phases

**DOI:** 10.1371/journal.pntd.0004997

**Published:** 2016-09-20

**Authors:** Carolina Hernández, Zulma Cucunubá, Carolina Flórez, Mario Olivera, Carlos Valencia, Pilar Zambrano, Cielo León, Juan David Ramírez

**Affiliations:** 1 Red Chagas Colombia, Instituto Nacional de Salud, Bogotá, Colombia; 2 Grupo de Parasitología, Instituto Nacional de Salud, Bogotá, Colombia; 3 Grupo de Investigaciones Microbiológicas-UR (GIMUR), Programa de Biología, Facultad de Ciencias Naturales y Matemáticas, Universidad el Rosario, Bogotá, Colombia; US Food and Drug Administration, UNITED STATES

## Abstract

**Background:**

The diagnosis of Chagas disease is complex due to the dynamics of parasitemia in the clinical phases of the disease. The molecular tests have been considered promissory because they detect the parasite in all clinical phases. *Trypanosoma cruzi* presents significant genetic variability and is classified into six Discrete Typing Units TcI-TcVI (DTUs) with the emergence of foreseen genotypes within TcI as TcIDom and TcI Sylvatic. The objective of this study was to determine the operating characteristics of molecular tests (conventional and Real Time PCR) for the detection of *T*. *cruzi* DNA, parasitic loads and DTUs in a large cohort of Colombian patients from acute and chronic phases.

**Methodology/Principal Findings:**

Samples were obtained from 708 patients in all clinical phases. Standard diagnosis (direct and serological tests) and molecular tests (conventional PCR and quantitative PCR) targeting the nuclear satellite DNA region. The genotyping was performed by PCR using the intergenic region of the mini-exon gene, the 24Sa, 18S and A10 regions. The operating capabilities showed that performance of qPCR was higher compared to cPCR. Likewise, the performance of qPCR was significantly higher in acute phase compared with chronic phase. The median parasitic loads detected were 4.69 and 1.33 parasite equivalents/mL for acute and chronic phases. The main DTU identified was TcI (74.2%). TcIDom genotype was significantly more frequent in chronic phase compared to acute phase (82.1% vs 16.6%). The median parasitic load for TcIDom was significantly higher compared with TcI Sylvatic in chronic phase (2.58 vs.0.75 parasite equivalents/ml).

**Conclusions/Significance:**

The molecular tests are a precise tool to complement the standard diagnosis of Chagas disease, specifically in acute phase showing high discriminative power. However, it is necessary to improve the sensitivity of molecular tests in chronic phase. The frequency and parasitemia of TcIDom genotype in chronic patients highlight its possible relationship to the chronicity of the disease.

## Author Summary

Chagas disease is a neglected tropical disease caused by the parasite *Trypanosoma cruzi* that shows tremendous genetic diversity evinced in at least six Discrete Typing Units and massive genetic diversity within TcI. Two clinical phases exist where acute phase shows high parasitemia and chronic phase shows low and intermittent parasite dynamics. One particularity of the disease is the diagnosis, because the parasitemia is highly variable during the phases of the disease. Molecular tests allow detecting DNA of the parasite in all clinical phases. Herein, we determined the operating characteristics of two molecular tests (cPCR and qPCR) to evaluate the performance of these tests for diagnosis of Chagas disease in 708 Colombian patients. We determined the parasitic loads and DTUs to assess how is the behaviour of these characteristics in relation to the clinical phases. We found that the performance of qPCR was higher compared to cPCR and the molecular tests are a precise tool for diagnostic of Chagas disease, mainly in the acute phase. The parasitemia was higher in the acute phase compared to chronic phase and the DTU predominant in Colombian patients was TcI. The behaviour of TcIDom genotype in the chronic phase patients evidenced possible relationship with the chronicity of the disease.

## Introduction

Chagas disease is a zoonotic parasitic disease caused by the protozoan *Trypanosoma cruzi*. It is considered a public health problem in Latin-America, where approximately 6 million people are currently infected [1]. The acute phase of the disease is characterised by usually mild fever that in a small proportion of cases can be accompanied by myocarditis and other lethal complications. Most of the patients continue through the chronic phase that is initially characterised by an asymptomatic clinical course during two or three decades, and about 30% of the infected patients will develop heart or digestive complications afterwards [2].

*T*. *cruzi* parasite shows significant genetic variability and classified into at least six Discrete Typing Units TcI-TcVI (DTUs), that present associations with the geographical distribution, epidemiological transmission cycles, insect vectors and clinical manifestations of Chagas disease [3–5]. Recent studies suggest the occurrence of an emerging clade within TcI named TcIDom which is distributed in the Americas and associated with domestic cycles of transmission and human infection [6–10]. Recently, a genotype detected in anthropogenic bats and named as TcBat has been described in Panama, Ecuador, Colombia and Brazil including a case of human infection in Colombia [11–14].

The diagnosis of Chagas disease is complex due to the dynamics of parasitemia in the phases of the disease. During the acute phase the parasitemia is high, therefore the diagnosis is performed by direct parasitological tests [15,16]. Nevertheless, direct parasitological tests are not useful in the chronic phase due to the low and intermittent parasitemias. Therefore, the diagnosis of Chagas disease in the chronic phase is determined by serological tests such as ELISA: enzyme-linked immunosorbent assay, IFA: indirect immunofluorescence assay or HAI: Hemagglutination Inhibition Test [17–19]. Recently, molecular techniques such as cPCR (conventional PCR) and qPCR (quantitative real-time PCR) have been considered as supportive diagnostic tests due to their ability to determine parasitic loads of *T*. *cruzi* in all clinical phases of the disease [20–22]. The operating characteristics of molecular tests for diagnosis of *T*. *cruzi* infection have varied according to clinical phase and technical specifications. Sensitivity for identifying chronic infection with cPCR has ranged between 22 and 75% [23,24] and in both cases with a specificity of 100%. Contrastingly, for qPCR, sensitivity has ranged between 60 and 80% [22,25,26] in chronic phase and between 88% and 100% for acute phase [25,26], whereas specificity is between 70–100% [26–28]. Sampling methods have not been always clearly stated and the role of these techniques for diagnosis of Chagas disease in the different clinical phases still remains poorly understood.

The objective of this work was to determine the operating capabilities of qPCR and cPCR targeting the satellite nuclear DNA region, compared with standard diagnosis methods for acute and chronic Chagas disease. Additionally, we evaluated the plausible associations between parasitic load and DTUs in Colombian patients from the acute and chronic phases to untangle the natural course of *T*. *cruzi* infection in terms of parasite dynamics.

## Materials and Methods

### Participants

All patients who attended the Colombian National Health Institute (Overall 985 individuals) seeking diagnostic tests for Chagas disease in acute (113 patients) or chronic phase (872 patients) between 2004 and 2015 were considered as potential participants. Inclusion criteria were: i. Clinical or epidemiological suspect of Chagas disease in acute or chronic phase ii. Not having received aetiological treatment for Chagas disease iii. Positive serological tests for Chagas disease (IFA, ELISA and/or HAI) iv. Adequate blood and serum samples available for performing diagnostic tests according to the clinical phase. v. Acceptance to participate and sign the informed consent.

### Ethical statement

The Technical Research Committee and Ethics Research Board at the National Health Institute in Bogotá, Colombia approved the study protocol CTIN-014-11. Participation was voluntary and patients were asked for informed written consent authorising to take blood and serum samples and access information on their clinical records.

### Sample size calculation and sampling methods

The total sample size (N) was calculated for test binary outcomes and separately for each clinical phase: acute and chronic. Considering, n = Z^2^ S (1−S) *d*^2^, where for a confidence level of 95% (1- α, with α = 0.05) Z is inserted by 1.96, and a maximum marginal error of estimate, *d*, is a desired value for precision based on researchers judgment, and S is a pre-determined value of sensitivity [29]. Based in previous studies, for the acute phase S was pre-established at 92% and with *d* at 8% [25,26], whereas for chronic phase S was pre-established at 60% with *d* at 5% [22–26]. Then, N = n /P, being P the estimated prevalence in this specific population under study. Given this is a selected population, composed of patients with some suspicion of the infection and remitted to a reference centre, P was specified at 60% in suspected cases for both acute and chronic phases. This value was obtained as an approximation based on the laboratory records at the NHI (Bogota, Colombia). The minimum total sample sizes were then calculated as N = 74 and N = 615 for suspected cases in acute and chronic phases respectively. The tests were performed to all subjects without knowing their previous clinical status. Clinical evaluation was conducted simultaneously to all individuals as part of the study to determine health status and then to the confirmed cases to evaluate heart complications. The inclusion of participants was conducted retrospectively for the period 2004 to 2012, and prospectively between 2013 and 2015. At the end, a total of 86 suspected acute patients and 622 suspected chronic patients were included in the study (Table 1).

**Table 1:** General characteristics of patients included in the study.

General characteristics		Acute phase^b^ N = 86		Chronic phase^c^ N = 622	
		Positive	Negative	Positive	Negative^d^
**Patients number (N)**	708	71	15	481	141
**Age, median (Q1-Q3)**^**a**^	48 (47–49)	31 (26–35)	27 (23–30)	51 (50–53)	37 (39–41)
**Sex, n (%)**					
**Female**	428 (60.4)	26 (36.6)	8 (53.3)	313 (65.1)	60 (42.5)
**Male**	280 (39.6)	45 (63.4)	7 (46.7)	168 (34.9)	81 (52.4)

^**a**^ Age in years

^b^ Positive patients were those who had positive direct parasitological tests, symptomatology and/or serological tests. Negative patients comprise a group of febrile patients with negative serology for Chagas disease and diagnosed with dengue.

^**c**^ Positive patients were those who had two positive serological tests and negative patients were those with two negative serological tests.

^**d**^ Twenty-nine were negative without risk factor and 112 negative with risk factor

### Clinical classification

#### Acute phase

a suspected case was defined as an individual with > 7 days of fever accompanied or not by hepatomegaly or splenomegaly. The patient was considered with acute Chagas disease if additionally to symptoms tested positive by parasitological tests (Strout, micro-strout, blood thick smear, or hemoculture) [15] or presented positive results to two serological tests over the course of the following weeks [30,31]. The patients were classified as negative to Chagas disease otherwise noted.

#### Chronic phase

individuals without criteria for acute phase but with clinical or epidemiological suspicion of Chagas disease. The patients were confirmed as positive *T*. *cruzi* infection when tested positive to two serological tests (IFA, ELISA and/or HAI). It was then classified as chronic undetermined (when no evidence of signs or symptoms of heart complications were evinced) or chronic determined otherwise.

The risk factors classification was conducted through a survey applied to each of the patients included in the study. A series of questions were asked such as the place of birth, knowledge of vector insects, blood donations and/or organ transplantation, housing type and presence of cardiac symptoms based on previous evaluated questionnaires (Survey 1) [32]. Patients whose serological tests were negative were classified into two groups according to the presence or absence of risk factors. The patients, who had one or more risk factors, were categorized as "negative with risk factors" and those patients that did not have any risk factors were categorized as "negative without risk factors".

### Laboratory tests

#### Parasitological methods

The direct parasitological methods were performed (Strout, micro-strout, blood thick smear, or hemoculture) according to the methodology described by Freilij et al., 1983 [33]. The results were considered positive when morphology compatible with the *T*.*cruzi* was observed. All samples were analysed without knowledge of the clinical status or other test.

#### Serological tests

Enzyme-linked immunosorbent assay (ELISA), immunofluorescence antibody assay (IFA) or hemaglutination-inhibition assay (HAI) were originally standardized at the National Health Institute [34] with *T*. *cruzi* strains belonging to TcI. All serological tests were conducted in duplicate and positive and negative controls were used for each assay. ELISA test was considered as positive when absorbance was ≥0.300, IFA when titres were ≥1/32 and HAI when titres were ≥1/32.) (S1 Appendix). All samples were analysed without knowledge of the clinical status or other tests. The indeterminate results in the serology tests (ELISA and IFI) were resolved by use of HAI test.

#### Molecular diagnostic tests

10 mL of blood samples were collected and stored with equal volume of Guanidine Hydrochloride 6M, EDTA 0.2 M buffer, pH 8.00 (GEB) and subsequently stored at 8°C. 5mL of serum was frozen at -70°C as described elsewhere [25,35]. 300 μL aliquots of GEB were employed and 5μL of IAC plasmid (40pg/μL) were added as internal control. The samples were submitted to DNA extraction using the High Pure PCR Template Roche kit according to Duffy et al., 2013. Conventional PCR (cPCR) and multiplex quantitative PCR (qPCR) for detection of satellite DNA of *T*. *cruzi* and IAC plasmid DNA were performed as reported elsewhere [23,26]. The qPCR test was considered positive when the amplification exceeded the threshold of fluorescence 0.01 and cPCR when was observed a DNA fragment of 166 bp in the electrophoresis. The positive samples for satellite nuclear PCR (qPCR and cPCR), were confirmed by kPCR. Parasitic loads by qPCR were measured as parasite equivalents per mL according to Moreira et al., 2013, using a TcI strain as standard curve (MHOM/CO/01/DA] [22]. All samples were analysed without knowledge of the clinical status or other tests (S1 Appendix).

#### DTUs discrimination

PCR was performed using five different molecular markers aimed at detecting the six DTUs and the two subdivisions of TcI previously described by other authors (TcIDom and TcI sylvatic) as recommended elsewhere [36–41] (S1 Appendix and S1 Fig).

### Statistical analysis

Operating characteristics of the molecular tests were estimated by comparing against standard diagnosis (described above). Sensitivity, specificity, positive (+LR) and negative likelihood ratio (LR-), predictive values (PV), diagnostic precision (DP), Area under the curve (AUC), and Kappa index (K) were estimated for each phase of the disease (acute and chronic), the clinical stage of chronic patients (determined and undetermined) and according to DTUs and TcI genotypes identified (TcI sylvatic/TcIDom) (S2 Appendix). Results are presented as percentages, with corresponding 95% confidence intervals (95% CI). Additionally, operational capabilities in chronic patients were calculated in two ways: the first including negative patients without risk factors since they are the true negative and the second including all the negative patients (with and without risk factors). Due to over dispersion of parasitic loads, medians and quartiles are presented. Comparisons are based on Mann-Whitney test between clinical phases, chronic clinical stages and the different *T*. *cruzi* DTUs and genotype groups identified. A p value at <0.05 was considered as statistically significant. All analysis was performed in Stata: Data Analysis and Statistical Software version 12.

## Results

### General characteristics of the patients included in the study

**Fig 1:**
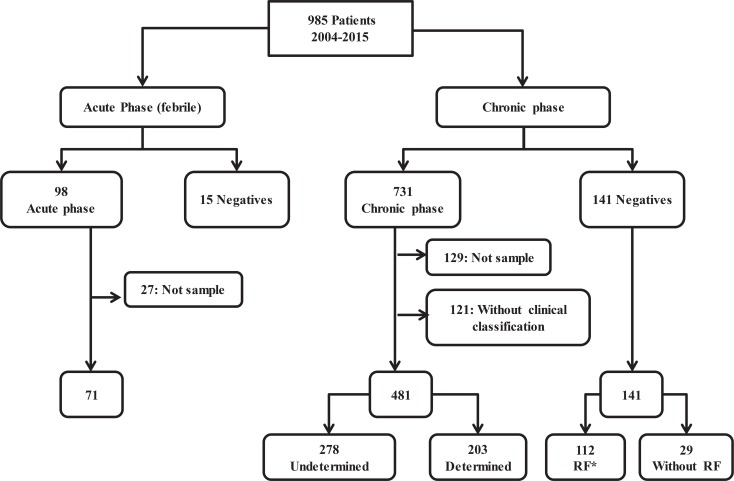
Algorithm for selection and classification of patients. There were selected 708 patients, 71 in acute phase, 15 febrile negatives, 481 in chronic phase and 141 negatives. *RF: Risk Factor.

Overall, 985 patients were included, 872 suspected of chronic and 113 of acute infection. General demographic characteristics are shown in Table 1. Out of the initial potential participants, 27 and 129 were excluded for incomplete samples to perform all analysis from the acute and chronic groups, respectively and 121 from the chronic group due to absence of clinical information (Fig 1). The inclusion of patients was prospective, whereas the sample collection was both retrospective (for the period 2004–2011) and prospective (for the period 2012–2015). This means that for the retrospective component the samples were part of the repository. The repository consists of 144 samples, collected between 2004 and 2011, and corresponds to serum samples stored at (-80°C). In these samples, serological tests were repeated and it was found that the results were the same that they had been reported at the time of collection of samples and molecular tests were performed. The prospective component consists of 564 samples, collected in the period between 2012 and 2015, and maintained in guanidine hydrochloride solution until processing.

In patients from the acute phase, the qPCR test was positive in 95.7% of the patients and cPCR in 84.5%. In patients from the undetermined chronic phase, qPCR was positive in 68.0% of the cases and in 55.4% by cPCR. In the cardiac chronic phase, qPCR positivity was 59.1% and 58.6% by cPCR. The positive samples for satellite nuclear PCR (qPCR and cPCR), were confirmed by kPCR. In patients that were negative by serology but with risk factors cPCR (2.6%) and qPCR (3.6%) were positive. In febrile and negative patients without risk factors both tests were negative in all samples. In all samples analyzed we detected the internal amplification control for both cPCR and qPCR, the average Ct value in all samples tested was 21.

### Operating characteristics of molecular methods vs standard diagnostic tests

The operating characteristics including all negatives patients of chronic phase (Negatives with and without risk factors) are presented in Tables 2 and 3.

**Table 2:** Operating characteristics of molecular tests in acute and chronic phases including all negative patients (with and without risk factors).

Operating characteristics	Acute phase N = (71/86)		Chronic phase N = (481/622)	
	qPCR (95% CI)	cPCR (95% CI)	qPCR (95% CI)	cPCR (95% CI)
**Sensitivity**	95.7 (88.3–98.5)	84.5 (74.3–91.2)	64.2 (59.8–68.4)	56.8 (52.3–61.1)
**Specificity**	100.0 (79.6–100.0)	100.0(79.6–100.0)	97.1 (92.9–98.8)	97.9 (93.9–99.2)
**PPV**	100.0 (94.6–100.0)	100.0 (93.9–100.0)	98.7 (96.7–99.5)	98.9(96.8–99.6)
**NPV**	83.3 (60.8–94.2)	57.69 (38.9–74.5)	44.3 (38.9–49.9)	39.8 (34.9–45.1)
**DP**	96.5 (90.2–98.8)	87.2 (78.5–92.8)	71.7 (68.0–75.1)	66.0 (62.3–69.6)
**LR+**	Undefined		22.6 (13.82–37.09)	26.68 (13.8–51.5)
**LR-**	0.04 (0.02–0.1)	0.15 (0.13–0.18)	0.37 (0.36–0.37)	0.44 (0.43–0.45)
**Kappa index**	0.89 (0.7–1.1)	0.62 (0.5–0.9)	0.43 (0.36–0.49)	0.36 (0.29–0.42)
**AUC**	0.98 (0.91–0.99)	0.92 (0.88–0.96)	0.81 (0.77–0.84)	0.77 (0.74–0.81)

PPV: Positive predictive value; NPV: Negative predictive value; DP: diagnostic precision; LR+: positive likelihood ratio; LR-: negative likelihood ratio. N = (Positive gold standard/ total assayed). When the specificity is 100% the positive likelihood ratio is undefined.

**Table 3:** Operating characteristics of molecular tests in chronic phases (undetermined and determined) of Chagas disease including all negatives patients (with and without risk factors).

Operating characteristics	Chronic undetermined phase N = 278/419		Chronic determined phase N = 203/344	
	qPCR (95%CI)	cPCR (95%CI)	qPCR (95%CI)	cPCR (95%CI)
**Sensitivity**	67.9 (62.3–73.1)	55.4 (49.5–61.1)	59.1 (52.2–65.6)	58.6 (51.7–65.1)
**Specificity**	97.2 (92.9–98.8)	97.9 (93.9–99.2)	97.2 (92.9–98.8)	97.9 (93.9–99.2)
**PPV**	97.9 (94.8–99.2)	98.0 (94.5–99.3)	96.7 (92.0–98.7)	97.5 (93.0–99.1)
**NPV**	60.6 (54.1–66.7)	52.7 (46.6–58.6)	62.2 (55.7–68.4)	62.1 (55.6–68.2)
**DP**	77.8 (73.6, 81.5)	69.6 (65.1–73.9)	74.7 (69.86–79.0)	74.7 (69.9–79.0)
**LR+**	24.0 (14.6–39.3)	26.0 (13.4–50.5)	20.8 (12.6–34.4)	27.5 (14.2–53.5)
**LR-**	0.33 (0.32–0.33)	0.45 (0.44–0.46)	0.42 (0.41–0.43)	0.42 (0.41–0.43)
**K**	0.57 (0.48–0.65)	0.44 (0.36–0.52)	0.52 (0.42–0.61)	0.51 (0.42–0.61)
**AUC**	0.83 (0.79–0.86)	0.77 (0.72–0.80)	0.77 7(0.73–0.82)	0.77 (0.73–0.82)

PPV: Positive predictive value; NPV: Negative predictive value; DP: diagnostic precision; LR+: positive likelihood ratio; LR-: negative likelihood ratio. N = (Positive gold standard/ total assayed).

**Fig 2:**
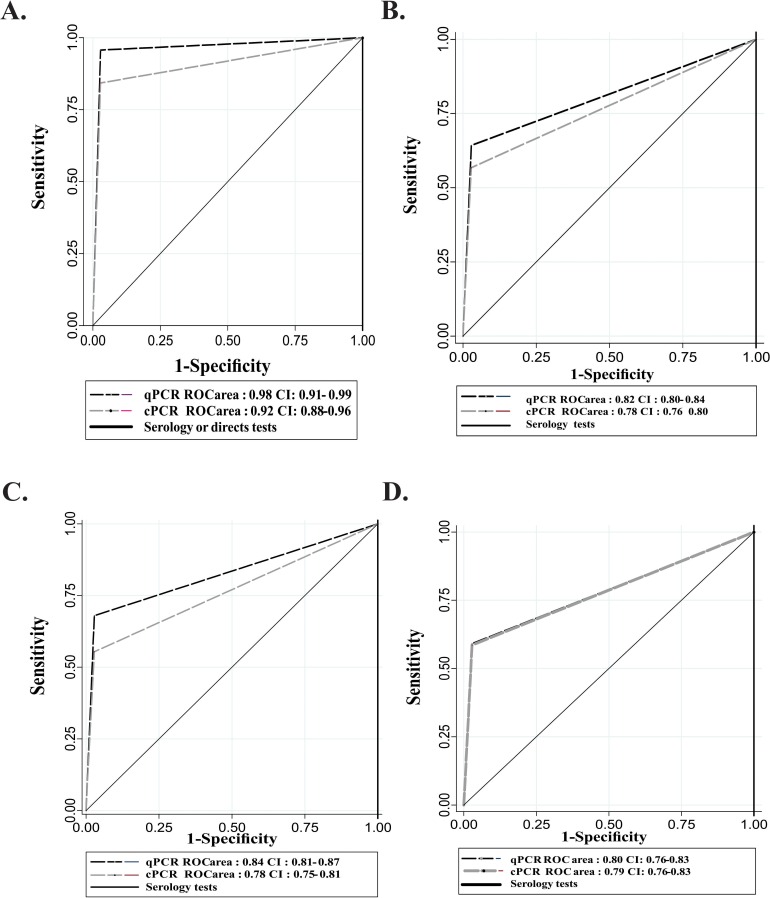
ROC curves of molecular tests in clinical phases of Chagas disease. **A**. Acute phase **B**. Chronic phase **C**. Chronic undetermined phase **D**. Chronic determined phase.

Performance of qPCR was higher compared to cPCR in both acute (AUC 0.98 vs 0.92) and chronic phase including only negatives with risk factors (0.82 vs 0.78) (Fig 2). Likewise, the performance was significantly higher in acute compared with chronic phase and in overall a specificity higher than sensitivity particularly in chronic phase (Tables 2, 4 and 5).

**Table 4:** Operating characteristics of molecular tests in chronic phase of Chagas disease including only negatives without risk factors.

Operating characteristics	Chronic phase N = (481/510)	
	qPCR (95% CI)	cPCR (95% CI)
**Sensitivity**	64.2 (59.9–58.4)	56.8 (52.3–61.1)
**Specificity**	100.0 (88.3–100.0)	100 (88.3–100.0)
**PPV**	100.0 (98.8–100.0)	100.0 (98.6–100.0)
**NPV**	14.4 (10.2–19.9)	12.2 (8.7–17.0)
**DP**	66.3(62.1–70.2)	59.2 (54.9–63.4)
**LR+**	Undefined	
**LR-**	0.36 (0.35–0.36)	0.43 (0.42–0.44)
**Kappa index**	0.17(0.1213–0.218)	0.13 (0.09–0.17)

PPV: Positive predictive value; NPV: Negative predictive value; DP: diagnostic precision; LR+: positive likelihood ratio; LR-: negative likelihood ratio. When the specificity is 100% the positive likelihood ratio is undefined. N = (Positive gold standard/ total assayed)

**Table 5:** Operating characteristics of molecular tests in chronic phases (undetermined and determined) of Chagas disease including only negatives without risk factors.

Operating characteristics	Chronic undetermined phase N = 278/307		Chronic determined phase N = 203/232	
	qPCR (95%CI)	cPCR (95%CI)	qPCR (95%CI)	cPCR (95%CI)
**Sensitivity**	68.0 (62.3–73.2)	55.4 (49.5–61.1)	59.1 (52.2–65.7)	58.6 (51.8–65.2)
**Specificity**	100 (88.3–100)	100.0 (88.3–100.0)	100 (88.3–100)	100.0 (88.3–100.0)
**PPV**	100 (98.0–100)	100.0 (97.6–100.0)	100 (96.9–100)	100.0 (96.8–100.0)
**NPV**	24.6 (17.7–37.1)	18.9 (13.5–25.9)	25.9 (18.7–34.7)	25.6 (18.5–34.4)
**DP**	71.0 (65.7–75.8)	59.6 (54.0–64.9)	64.2 (57.9–70.1)	63.6 (57.2–69.6)
**LR+**	Undefined			
**LR-**	0.32 (0.31–0.33)	0.44 (0.43–0.45)	0.41 (0.39–0.41)	0.41 (0.40–0.43)
**K**	0.29 (0.21–0.36)	0.19(0.12–0.25)	0.26(0.17–0.35)	0.26 (0.17–0.34)

PPV: Positive predictive value; NPV: Negative predictive value; DP: diagnostic precision; LR+: positive likelihood ratio; LR-: negative likelihood ratio. When the specificity is 100% the positive likelihood ratio is undefined.

### *T*. *cruzi* parasitic loads and clinical phase

**Fig 3:**
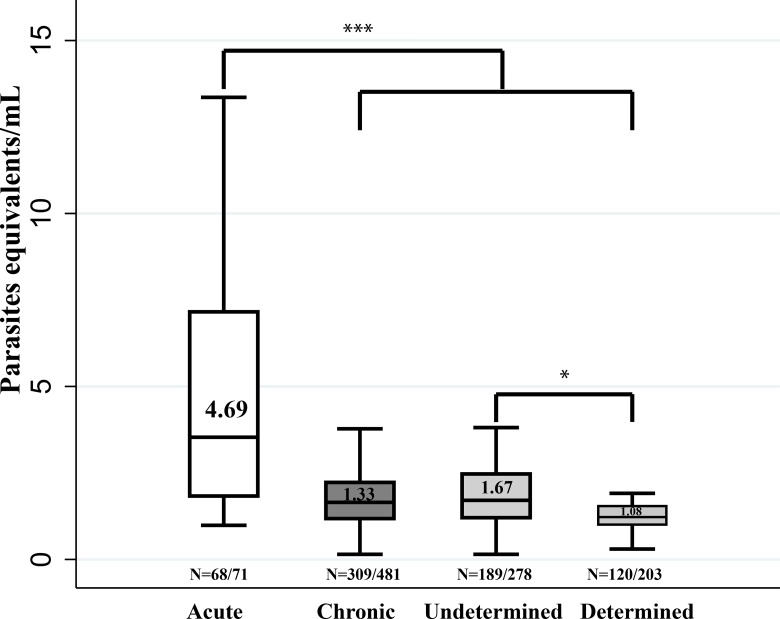
Comparative analysis of parasitic loads in patients with Chagas disease. Distribution of parasitic load and medians on the basis of the clinical phases. The outliers were removed from the graph for convenience. * p < 0.05 ** p<0.01 *** p < 0.001.

Parasitic loads were determined in samples that tested positive by qPCR. Significantly different median values were detected in acute (4.69 parasite equivalents/mL) versus chronic phase (1.33 parasite equivalents/mL). A statistically median difference was also found between determined and undetermined chronic phase (Fig 3).

### Molecular tests performance according to *T*. *cruzi* DTUs and clinical phase

**Fig 4:**
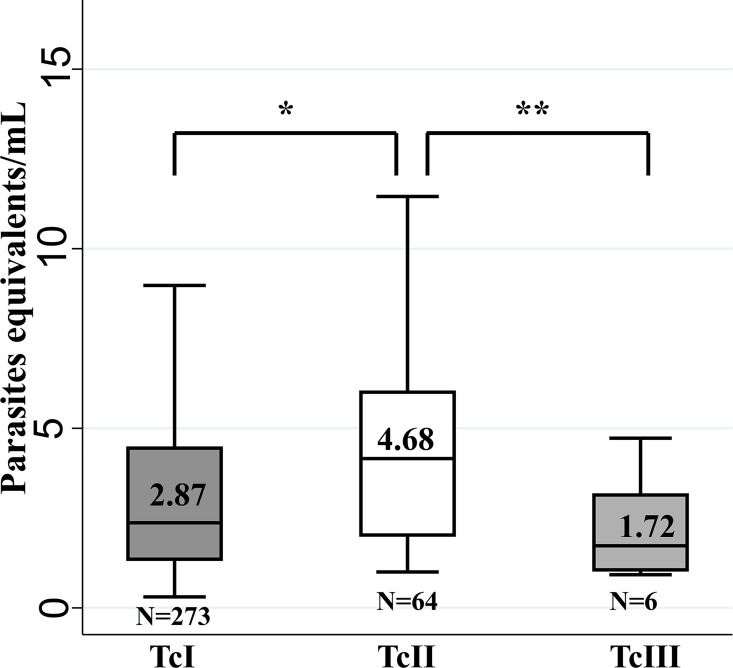
Comparative analysis of parasitic loads for DTUs. Distribution of parasitic load and medians on the basis of the *T*.*cruzi* DTUs. For convenience the outliers were removed for the graph. * p < 0.05 ** p<0.01 *** p < 0.001.

In samples that tested positive (n = 407) by cPCR, the DTUs TcI-TcVI and TcI (TcI Dom, TcI Sylvatic) were evaluated. The distribution of DTUs was 74.2% for TcI, 17.2% for TcII, 1.48% for TcIII, 0.5% for TcV and 6.7% for mixed infections. For the latter seven different combinations were identified: TcIDom/TcII/TcV, TcIDom/TcII, TcIDom/TcISylv, TcIDom/TcISylv/TcII, TcIDom/TcISylv/TcIII, TcIDom/TcIV, TcISylv/TcII. With respect to TcI, the genotyping was feasible in 290/302 samples. Out of them, 28.7% were classified as TcI Sylvatic and 71.4% as TcIDom. The median load parasitic value for TcII (4.68 parasite equivalents/mL) was significantly different to the one for TcI (2.87 parasite equivalents/mL) and TcIII (1.72 parasite equivalents/mL) (Fig 4).

**Fig 5:**
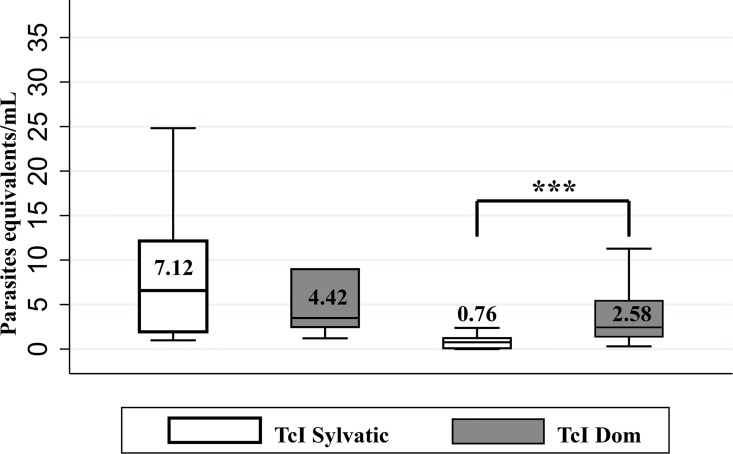
Comparative analysis of parasitic loads for TcI Genotypes in the clinical phases. Distribution of parasitic load and medians on the basis of the TcI genotypes in the acute and chronic phases. For convenience the outliers were removed for the graph. * p < 0.05 ** p<0.01 *** p < 0.001.

The genotype distribution according to clinical phase evidenced that TcIDom was significantly more frequent in chronic phase compared with acute phase (Table 6). The operating characteristics of molecular tests for the different genotypes were calculated, observing that the sensitivity for identifying TcII was slightly higher than for TcI, mainly for qPCR (S1 Table). The median parasitic load for TcIDom was significantly higher (2.58 parasite equivalents/ml) compared with TcI Sylvatic (0.76 parasite equivalents/ml) in chronic phase (Fig 5).

**Table 6:** Frequency of DTUs and TcI genotypes from clinical phases of Chagas disease patients.

DTUs	Clinical phases								P value
	Acute				Chronic				
	N = 68				N = 332				
	N	(%)	(95%CI)		n	(%)	(95%CI)		
TcI	54	79.4	69.3	88.9	241	72.6	67.8	77.4	0.25
TcII	6	9.0	2.0	15.9	64	19.3	15.0	23.5	**0.04**
TcIII	4	6.0	0.2	11.7	2	0.6	0.2	1.4	**<0.001**
TcV	-	-	-	-	2	0.6	0.2	1.4	0.53
Mixed	4	6.0	0.2	11.7	23	6.9	4.2	9.7	0.15
TcI Genotypes	N = 48				N = 235				
TcI Sylvatic	40	85.1	74.8	95.4	42	17.9	12.9	22.8	**<0.001**
TcI Dom	8	16.6	4.6	25.2	193	82.1	77.2	87.0	**<0.001**

DTU: Discrete Unit Typing; bold text: p value at <0.05

## Discussion

### Operating characteristics of molecular methods against standard diagnostic tests

The main limitation involved in this study is the fact that there is not a gold standard test for all clinical phases of Chagas disease. Particularly for chronic phase, the best comparators are serological tests but these techniques measure the immune response and not the relative presence of the parasite. This particular situation impacts the evaluation of new diagnostic tests. This is reflected mainly in the kappa index (Tables 2 and 4) that presented very low values in the undetermined and determined chronic phases. Unfortunately, it has not a simple solution and more understanding of the course of the infection is still needed.

The results obtained for the molecular diagnosis in acute phase were optimal in terms of sensitivity for both qPCR (95.7%; 95%CI: 88.3–98.5) and cPCR sensitivity (84.5%; 95%CI: 74.3–91.2), and same specificity. Although the results are showing a potential superior performance of the sensitivity of qPCR compared with cPCR, this difference needs a cautious interpretation. This might be explained due to the fact that detection by qPCR increases the sensitivity and specificity because of the hybridization of the Taqman probe in the amplicon, whereas in the case of the cPCR it requires a considerable amount of amplicon so that it can be observed in agarose gels [25,26] In addition, the confidence intervals were slightly overlapped, meaning that there is some indication of this difference but it is not statistically significant, so not definitive. The performance of the molecular tests in the acute phase is explained because there are large numbers of parasites, for example in cases of reactivation in immunosuppressed patients and in oral outbreaks. The values obtained for LR evinced the high probability that positive results correspond to diseased patients (LR+) and the low probability that the diseased patients present negative results (LR-). In addition, the DP was very optimal specifically for qPCR test confirming that this molecular test is very useful for the diagnosis in the acute phase, considering that the direct diagnosis is complex when the parasitemia is low (As is the case of the acute patients detected more than a month after the infection where the parasitemia normally begins to decrease due to the control of the immune response) and are required many tests for the confirmation of the acute cases (direct tests, serology tests and clinical information). Regarding the predictive power of molecular tests in the acute phase, these tests are very good predictors of the disease presence when positive results are obtained (PPV) but their performance as predictors of absence of the disease are less (NPV). However, it is worth noting that the predictive values depend on disease prevalence in the evaluated population.

The analysis of operational capabilities in the chronic phase was conducted in the first instance including only negative patients without risk factors or true negatives. For the chronic phase, qPCR sensitivity was 64.2% and 56.8% for cPCR and in concordance with previous reports obtained by qPCR that have shown sensitivity ranging from 60–80% and 20–70% for cPCR [22–24,26,28,42]. These sensitivity results may be due to low and intermittent parasitic loads during chronic phase.

The performance of qPCR was better than cPCR in the chronic undetermined phase, while that was very similar between the two tests in the determined chronic phase (Tables 3 and 5). The discriminative power of the two molecular tests was acceptable in the chronic phase. For qPCR, the AUC and DP values obtained (Tables 3 and 5) were better for the undetermined phase than for determined phase. The differences between undetermined and determined phases for qPCR of the chronic phase can be explained by the natural course of the disease, in which the parasitic load decreases while increases the infection time. This is supported by several studies showing that there is no relationship between the evolution of the cardiac form of the disease and parasitemia but it declines with time as observed in this study [43,44]. Also, some studies show that cardiac form is mainly related to different types of strains, increased parasitemia, reinfection or immune system disorders in chronic patients [45,46]. In the cPCR AUC values were the same for both phases, while the value of DP was best for the determined phase. Possibly, this is because the detection limit of the cPCR is lower than qPCR, for this reason the cPCR behaves similarly in the two phases. In the two stages of the chronic phase, there is a high probability that patients with negative results in the molecular tests have the disease (LR-) and these tests are not good predictors of the absence of the disease (NPV) (Table 5). Therefore, the use of molecular methods as diagnostic tests is not appropriate due to the better performance displayed by serology. The probability that the results are positive is high in diseased individuals with respect to healthy individuals (LR +) and the molecular tests are excellent predictors of the presence of disease (PPV). Thus, these tests could be used in situations in which the diagnosis is doubtful, allowing the confirmation of the parasite in diseased patients, which is of great importance for example when monitoring etiological treatment. However, it is necessary to improve the sensitivity, which can be performed by analysing serial samples for each patient as seen in some studies in which such sensitivity improved from 69.2% to 85.2% with the addition of a second sample or conducting DNA extraction from a larger volume of the sample [47,48].

In addition, the operating capabilities of patients in chronic phase were calculated including all negatives by serology with and without risk factors (Table 1, N = 141). It was observed in the group of negative patients with risk factors a positivity of 2.6% (3 patients) by cPCR and 3.6% (4 patients) by qPCR, possibly due to an immunosuppression issue in these patients preventing the detection of antibodies or infection. Three patients are from the department of Casanare, which is an endemic area, and five patients had less than 24 years of age suggesting a recent infection. Also, all patients reported to know the vectors and have lived during his/her childhood in homes with features such as thatched or ‘barheque’, floor or wood and/or tread walls of earth, wood or ‘barheque’. Two of the seven patients that were negative by serology and had risk factors, whose ages were 36 and 51 showed the presence of symptoms at cardiac level. In this group of 7 patients, 4 presented the ELISA absorbance values greater than 0.200 and 4 detectable titles in the IFA (1/8 and 1/16).

As the operating capabilities calculated including all negative patients, a small percentage of decreased specificity in the two platforms was observed (S3 Appendix). The positivity of these serologically negative patients that generated the decrease can probably be explained because cases of recent infection or patients with some form of immunosuppression that has generated the absence of detectable antibodies. In fact, in the group of acute patients, 4 patients whose serology was negative showed positive PCR, in these patients the detection was achieved by direct parasitological methods. Regarding the molecular techniques, given that in all PCR runs were included negative controls including reagents controls, a plausible contamination with parasite DNA is discarded. Significantly, the DP and AUC values showed no obvious changes unlike the values obtained for the NPV and the Kappa index, in which there was a marked increase. However, the changes obtained do not change the interpretation of the usefulness of the test in the clinical setting, but can show that there are few cases where serological tests may have false negatives as noted previously using cPCR by Ramirez et al., 2009 [23]. Even though serological tests are considered the best current option for the diagnosis of Chagas disease, in a meta-analysis of high quality tests their sensitivity has been estimated at 90% [49]. Given this, we believe that an improvement of diagnostic tests for Chagas disease is needed for both serology and PCR techniques. An appropriate use of the comparator as gold standard and the inclusion of different phases of the disease are crucial to understand the utility of different diagnostic tests.

To our knowledge, this is the first study to include statistical calculation of the sample, which allowed the analysis of operating characteristics of the molecular tests in all clinical phases of Chagas disease. In addition, this study is the first in analysing the two PCR platforms (qPCR and PCR) for the same target (stDNA) in patients from all clinical phases of Chagas disease. The conventional technique was included, due to the vast use of this technique in the diagnosis and its ease implementation in laboratories with restricted equipment (a Real Time PCR machine is not available) [23,24,28]. Lastly, acute patients had a less median age than chronic phase patients and in turn the largest number of acute cases are male. This possibly is because economic activity in endemic areas is developed by males that assist to the field and this facilitates direct patient contact with the vector and therefore with the parasite. On the other hand, females ratio and the median age were higher in chronic phase patients that are usually detected by screening blood banks or present cardiac abnormalities in chronic phase, then the detection occurs at a greater age. Additionally, in Colombia most blood donors are women facilitating their diagnosis.

### *T*. *cruzi* parasitemia, DTUs and clinical phases

Regarding the parasitemia, it is observed that the median parasitemia was higher in acute patients compared to chronic phase, which is expected given the dynamics of parasitemia in the disease [25,26]. As for the group of chronic patients, the herein reported median of parasitemia is similar to those previously reported for Colombia [22,26]. In addition, the difference in medians between cardiac chronic and undetermined chronic stages was statistically significant, being higher in the undetermined chronic phase unlike the findings described by Ramirez et al, 2015 [26], in which statistically significant difference was not detected. However, our results are in accordance with the natural history of the disease where parasitic loads decrease with the chronicity of the infection and this is probably associated with the type of strain and/or the immune response [2].

The DTU with highest frequency was TcI, both in acute and chronic patients, consistent with findings previously reported in Colombia [8,39,50,51]. Followed by TcII most often detected in chronic than acute patients. These findings are congruent due to the predominance of TcII in domestic cycles of transmission for the case of Colombia [50]. Regarding the parasitic loads of the DTUs detected, we observed that TcII had higher median parasitemia than other DTUs, consistent with the number of copies that has been reported in the DNA nuclear satellite region being higher for TcII than for TcI [52–54]. These findings highlight the importance of using the most representative DTU to generate the standard curves for quantification [22,25,26]. In addition, in murine models TcII shows higher parasitemias than TcI when performing individual and mixed infections [55].

In this study, acute cases are likely caused by vector transmission and possible oral route. In most of the cases TcI (TcI sylvatic), TcII and TcIII infection was observed. These findings are consistent with previously documented reports for acute patients where DTUs associated with the sylvatic cycle of transmission were depicted [4,5,40,51,56–61]. An interesting finding was the detection of TcV in the patients surveyed. This DTU has been already reported in dogs and *Rhodnius prolixus* from eastern Colombia but this would be the first report of TcV human infection in the country [50]. It is necessary to conduct further studies to understand the host-parasite associations of this foreseen DTU in patients from northern areas of the continent. It is well known that TcV infection is endemic in Bolivia, Brazil and Argentina but in Colombia is a novel case that requires further investigation; in fact high-resolution markers have been applied to the few isolates of Colombian TcV showing a tailored hybrid profile suggesting a Pan-American import from south America [62]. The DTU TcVI, is mainly detected in the South Cone of Latin America. Normally associated with megavisceral syndromes and some cases of congenital heart disease [4]. In Colombia, TcVI has been very rare and almost infrequent. In fact it is limited to a report in which was detected in humans and *R*. *prolixus* isolates (4% and 1.4% respectively). In addition, in different studies with a considerable number of patients conducted in Colombia it was not detected, confirming the low prevalence of the DTU in the country [39,51,63].

Recently, it has been highlighted the emergence of a genotype named as TcIDom and associated to human infection and domestic transmission cycles via different molecular markers [5,6,8,64–66]. Other studies have shown the presence of TcI Sylvatic genotype in tissue and TcIDom in bloodstream of patients with Chagas cardiomyopathy [41]. In murine models was observed that TcIDom produced high parasitemia and low tissue invasion, a process that allows an adaptation to the host prolonging its permanence and likely generation of chronicity, opposite process to what happened with the TcI sylvatic strains [67]. In accordance with these previous findings, our results show that in chronic patients the frequency and parasitemia of TcIDom genotype were significantly higher in chronic patients than in acute patients, supporting the hypothesis that this genotype may be related to chronicity in patients with Chagas cardiomyopathy.

In conclusion, the molecular diagnostic tests are becoming a precise tool to complement the standard diagnostic methods for Chagas disease. This study shows that in general qPCR has a better performance than cPCR. Also, the results confirm that PCR is highly specific for both acute and chronic clinical phases, whereas sensitivity is acceptable for acute phase but still very low for chronic patients. This situation could be partially explained by the higher parasitic loads detected in acute phase and the intermittent nature of the parasite release to the bloodstream in chronic phase. We explored for the first time in a large cohort of Chagas disease patients the DTU parasitemia and the natural course of infection. This type of studies is required in Latin-America for a better understanding of disease progression and molecular epidemiology of Chagas disease. This makes PCR a potential tool for its use in acute phase diagnosis in a routine basis, and potentially for determining aetiological treatment failure when tests positive but not substantially useful when tests negative and these results must be interpreted cautiously as in the clinical trials previously published [21,68]. Further research is needed to improve the sensitivity of this test and the mandatory deployment of new diagnostic tests.

## Supporting Information

S1 Fig Algorithm for genotyping of *T*. *cruzi* DTUs. Molecular characterization of *T*.*cruzi* by five molecular markers and genotyping of TcI DTU in two genotypes TcI Dom and *TcI Sylv: TcI Sylvatic.
(JPG)

S1 Table Operating characteristics of molecular test for DTUs and genotypes TcI. (DOC)

S1 Appendix Methodology of reference tests employed in the study. (DOCX)

S2 Appendix Detailed methodology of the molecular tools employed in the study. (DOC)

S3 Appendix Sensitivity and specificity calculations. (DOC)

S4 Appendix STARD. Standards for Reporting of Diagnostic Accuracy Checklist. (DOCX)

S5 Appendix Research protocol. Project: Characterization of a cohort of patients with Chagas disease, its etiological treatment, adverse events and therapeutic response.
(PDF)
