# Correction: Molecular Diagnosis of Chagas Disease in Colombia: Parasitic Loads and Discrete Typing Units in Patients from Acute and Chronic Phases

**DOI:** 10.1371/journal.pntd.0005112

**Published:** 2016-10-28

**Authors:** Carolina Hernández, Zulma Cucunubá, Carolina Flórez, Mario Olivera, Carlos A. Valencia-Hernandez, Pilar Zambrano, Cielo León, Juan David Ramírez

The fifth author's name is spelled incorrectly. The correct name is: Carlos A. Valencia-Hernandez. The correct citation is: Hernández C, Cucunubá Z, Flórez C, Olivera M, Valencia-Hernandez C.A., Zambrano P, et al. (2016) Molecular Diagnosis of Chagas Disease in Colombia: Parasitic Loads and Discrete Typing Units in Patients from Acute and Chronic Phases. PLoS Negl Trop Dis 10(9): e0004997. doi: 10.1371/journal.pntd.0004997
