# Supplementary figures and images for: Molecular Diagnosis of Chagas Disease in Colombia: Parasitic Loads and Discrete Typing Units in Patients from Acute and Chronic Phases
Source: PLoS Negl Trop Dis. 2016 Sep 20;10(9):e0004997. doi: 10.1371/journal.pntd.0004997 (PMC5029947; doi:10.1371/journal.pntd.0004997)

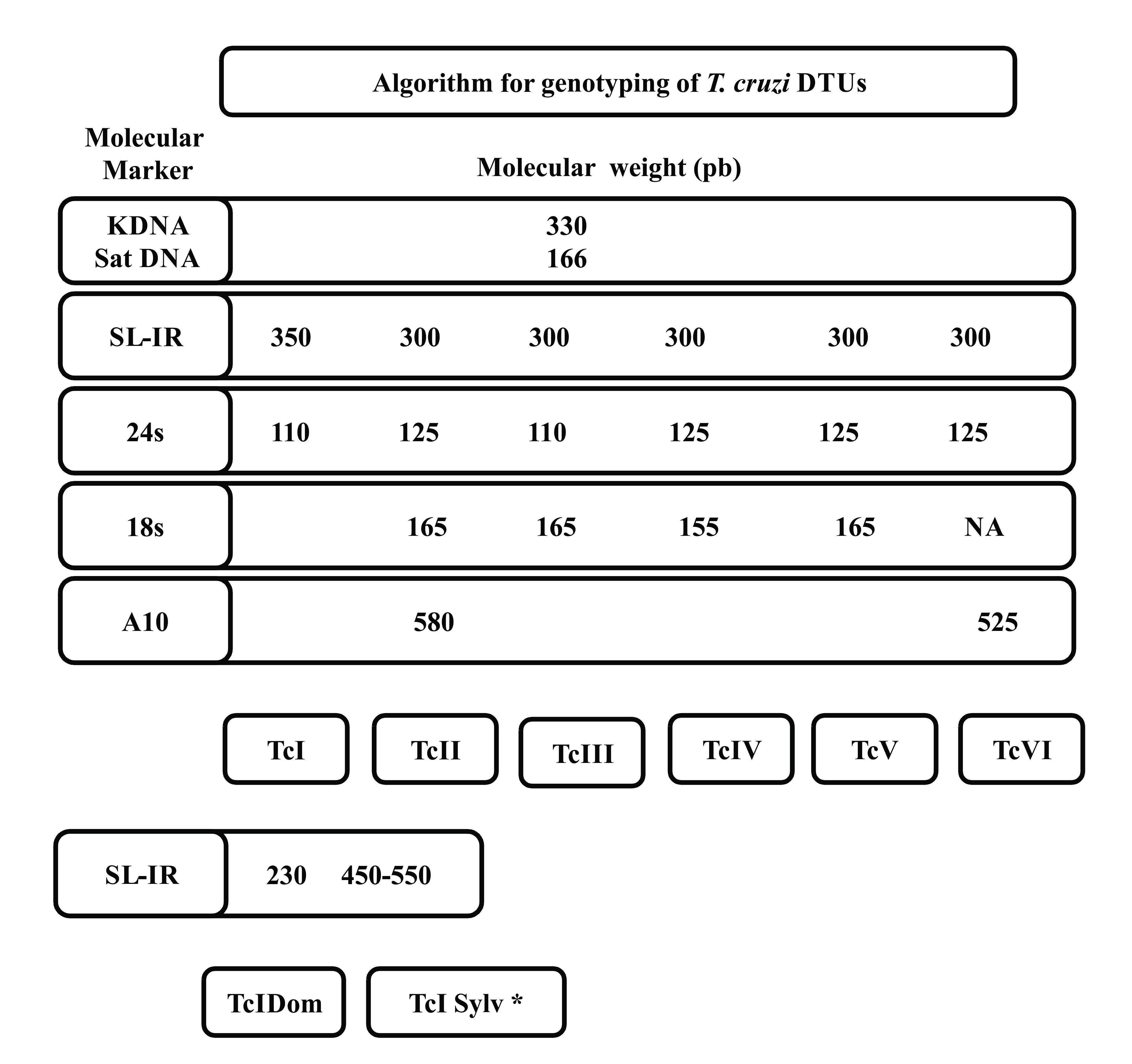

Supplement: S1 Fig — Molecular characterization of T.cruzi by five molecular markers and genotyping of TcI DTU in two genotypes TcI Dom and *TcI Sylv: TcI Sylvatic. (JPG) [file pntd.0004997.s001.jpg]
