# Supplementary material for: Molecular Diagnosis of Chagas Disease in Colombia: Parasitic Loads and Discrete Typing Units in Patients from Acute and Chronic Phases
Source: PLoS Negl Trop Dis. 2016 Sep 20;10(9):e0004997. doi: 10.1371/journal.pntd.0004997 (PMC5029947; doi:10.1371/journal.pntd.0004997)
Supplement: S1 Table — (DOC) [file pntd.0004997.s002.doc]

**Table S1. Operating characteristics of molecular test for DTUs and genotypes TcI**

| **Operating characteristics** | **TcI** | | **TcII** | |
| --- | --- | --- | --- | --- |
|  | **qPCR (95% CI)** | **cPCR (95% CI)** | **qPCR (95% CI)** | **cPCR (95% CI)** |
| **Sensitivity** | 90.4% (86.55, 93.23 ) | 82.12% (77.4, 86.03) | 91.43% (82.53, 96.01 ) | 82.86% (72.38, 89.91) |
| **Specificity** | 5.263% (2.434, 11.01) | 21.93% (15.32, 30.37) | 8.406% (5.916, 11.8) | 19.42% (15.59, 23.92) |
| **PPV** | 71.65% (66.93, 75.95) | 73.59% (68.64, 78.01) | 16.84% (13.42, 20.93) | 17.26% (13.6, 21.67) |
| **NPV** | 17.14% (8.103, 32.68) | 31.65% (22.45, 42.55) | 82.86% (67.32, 91.9) | 84.81% (75.3, 91.09) |
| **DP** | 67.07% (62.41, 71.41) | 65.63% (60.94, 70.03) | 22.41% (18.66, 26.66) | 30.12% (25.91, 34.7) |
| **LR+** | 0.9542 (0.9363 - 0.9724) | 1.052 (1.027 - 1.077) | 0.9982 (0.9892 - 1.007) | 1.028 (1.014 - 1.043) |
| **LR-** | 1.825 (0.004766 - 698.5) | 0.8154 (0.5948 - 1.118) | 1.02 (0.3522 - 2.952) | 0.8827 (0.664 - 1.173) |
| **K** | 0.05533 (-0.1316 - 0.02093) | 0.0447 (-0.04867 - 0.1382) | 0.000599 (-0.02642 - 0.02522) | 0.009057 (-0.03106 - 0.04917) |

**PPV: Positive predictive value; NPV: Negative predictive value; DP: diagnostic precision; LR+: positive likelihood ratio; LR-: negative likelihood ratio**

| **Operating characteristics** | **TcI Sylvatic** | | **TcIDom** | |
| --- | --- | --- | --- | --- |
|  | **qPCR (95% CI)** | **cPCR (95% CI)** | **qPCR (95% CI)** | **cPCR (95% CI)** |
| **Sensitivity** | 89.16% (80.66, 94.19) | 83.13% (73.66, 89.68) | 90.29% (85.48, 93.63) | 81.55% (75.7, 86.26 ) |
| **Specificity** | 7.831% (5.4, 11.23) | 19.58% (15.67, 24.18) | 7.177% (4.397, 11.5) | 8.197% (5.03, 13.08) |
| **PPV** | 19.47% (15.81, 23.75) | 20.54% (16.56, 25.18) | 48.95% (43.96, 53.96 ) | 50% (44.68, 55.32) |
| **NPV** | 74.29% (57.93, 85.84) | 82.28% (72.42, 89.14) | 42.86% (27.98, 59.14 ) | 28.3% (17.97, 41.57) |
| **DP** | 24.1% (20.23, 28.44) | 32.29% (27.97, 36.93) | 48.43% (43.66, 53.23 ) | 47.04% (42.14, 52.01) |
| **LR+** | 0.9673 (0.9581 - 0.9767) | 1.034 (1.02 - 1.047) | 0.9727 (0.9619 - 0.9837) | 0.8883 (0.8757 - 0.9011) |
| **LR-** | 1.385 (0.4586 - 4.18) | 0.8615 (0.6617 - 1.122) | 1.353 (0.2263 - 8.086) | 2.25 (0.4947 - 10.24) |
| **K** | 0.01286 (-0.0414 - 0.01568) | 0.01265 (-0.03141 - 0.05671) | 0.02516 (-0.07832 - 0.02799) | 0.1067 (-0.1779 - -0.03561) |

PPV: Positive predictive value; NPV: Negative predictive value; DP: diagnostic precision; LR+: positive likelihood ratio; LR-: negative likelihood ratio
