# Supplementary material for: Molecular Diagnosis of Chagas Disease in Colombia: Parasitic Loads and Discrete Typing Units in Patients from Acute and Chronic Phases
Source: PLoS Negl Trop Dis. 2016 Sep 20;10(9):e0004997. doi: 10.1371/journal.pntd.0004997 (PMC5029947; doi:10.1371/journal.pntd.0004997)
Supplement: S1 Appendix — (DOCX) [file pntd.0004997.s003.docx]

**Appendix 1. Methodology of reference tests employed in the study**

***Parasitological tests:***

- **Strout**: Obtain a blood sample (5-10 ml) without anticoagulant and allow clot retraction. Remove the clot and centrifuge serum obtained twice. Perform the first centrifugation for 5 minutes at 500-800 rpm and carefully remove (supernatant). Subject the supernatant obtained and centrifuge again for 10 minutes at 2500 r.p.m. Finally removing the supernatant and observe the pellet decanted under the microscope (1).
- **Micro-strout:** Obtain a blood sample in a capillary tube or heparinized micro-hematocrit. Centrifuge at 10,000 rpm for 3 min and microscopically observe the interface between the plasma and red blood cells (buffy coat) (1).
- **Hemoculture**: Collect 5 mL of whole blood into tubes with sodium citrate. Take 3 mL and seeding Tobie medium under sterile conditions and incubated at 28 ° C for six months performing constant observation (1).
- **Blood thick smear**: Place a drop of blood, about 2 mm in diameter approximately inch from the frosted area of the slide. Place the slide on a flat surface, and hold the narrow side of the nonfrosted edge between your left thumb and forefinger.With your right hand, place the smooth clean edge of a second (spreader) slide on the specimen slide, just in front of the blood drop. Hold the spreader slide at a 30 angle, and draw it back against the drop of blood. Allow the blood to spread almost to the edges of the slide.Push the spread forward with one light, smooth, and fluid motion. A thin film of blood in the shape of a bullet with a feathered edge will remain on the slide. Label the frosted edge with patient name, ID# and date. Allow the blood film to air-dry completely before staining. Attach a clothes pin (or use forceps) to the thick edge of the blood smear. Place the slide in the Coplin jar with Wright’s stain. Allow to stand 5-10 seconds. Place the slide in the jar containing deionized water. Allow to stand 10-20 seconds.Remove the slide carefully and dip several times in the second jar containing deionized water to rinse off the excess stain. Wipe off excess fluid from the back of the slide. Place the slide upright on a paper towel with the feathered edge up and allow to air dry. When completely dry, examine the smear with the microscope as follows (1).

The results were considered positive when morphology compatible with the *T.cruzi* was observed.

***Serological tests***

- **ELISA:**
  - **Antigen sensitization:** Add to each of the wells plate 100μl of optimal concentration of antigen. Incubate for 3 hours in a humid chamber at 4 ° C ± 1 C. Perform three washings of the microplate, if manually, using 300 μl of PBS-working solution Tween 0.1 M pH 7.4 in each well, for 5 minutes . If the plate washings are performed in automated microplate washer, use the computer card (4 washes x 12 strips). Either way, the washings must be performed with PBS working solution-Tween 0.1 M pH 7.4 to remove traces of the antigen and thus prevent a pH change in the reaction.

- - **Optimal antigen concentration:** Perform the dilution in 0.05 M carbonate buffer pH 9.6, whose main function is to stabilize the antigen in the optimal working dilution. This concentration is standardized at 7.5 mg/ml, and according to standardized and constant primary method validation.

- - **Optimal Sample Dilution:** Samples problems and known samples used as controls should be diluted 1: 1000 for serum (10 μl of sample in 10 ml of PBS-Tween buffer).
  - **Adding of sample:** Add 100 μl duplicate of the optimal dilution of each of the patient samples under study and respective known controls. Leave 2 hours of incubation in a humid chamber at room temperature 20 ° C ± 5 ° C. Perform three washings of the microplate, if manually, using 300 ul of PBS-working solution Tween0.1 M pH 7.4 in each well, for 5 minutes. If the plate washings are performed in automated microplate washer, use the computer card (4 washes x 12 strips). Either way, the washings must be performed with PBS working solution Tween0.1 M pH 7.4 to remove traces of the antigen and thus prevent a pH change in the reaction.
  - **Optimal dilution of conjugate (anti - human IgG coupled to alkaline phosphatase):** The conjugate used (anti - human IgG coupled with alkaline phosphatase) should be titrated previously to obtain the optimum working dilution. Titration is performed with known samples used as controls, considering using dilutions of titles with commercial conjugate from 1:1000 to 1:12000. Dilutions were made in PBS-Tween buffer.
  - **Conjugate addition:** Add 100 μl of the optimal dilution of conjugate anti - IgG coupled with alkaline phosphatase in each of the wells. Incubate for 8 hours in a humid chamber at 4 ± 1 C. Perform three washings of the microplate, if manually, using 300 ul of PBS-working solution Tween0.1 M pH 7.4 in each well, for 5 minutes . If the plate washings are performed in automated microplate washer, use the computer card (4 washes x 12 strips) .In one way or another, be performed washings with PBS working solution Tween0.1 M pH 7 4 to remove traces of the antigen and thus prevent a pH change in the reaction**.**

- - **Adding or chromogenic substrate:** Add 100 μl of substrate dilution (To-nitrophenyl phosphate / Diethanolamine) and incubate in a humid chamber for 30 minutes at room temperature 20 ° C ± 5 ° C.

- - **Interruption of the reaction:** Finally quench with 25 μl of sodium hydroxide (NaOH) 3 M Sodium hydroxide also provides the immunoenzymatic reaction increased intensity and stability of yellow tone which gives the chromogenic reaction.

- - **Reading:** Perform reading of the optical density of each of the samples, for which a Multiskan photocolorimeter is used at a wavelength of 405 nm. ELISA test was considered positive as absorbance when was ≥0.300 (2).
- **IFA:** Label the polystyrene plates with U-bottom identification numbers of samples to be processed. Add 25 μl of PBS buffer pH 7.2 - 7.6 in all wells of the plate. To make the dilutions, add 25 μl of the serum sample positive internal control 1 to the first well of the first column, 25 μl of serum positive internal control 2 to the first well of the second column, 25 μl of negative control serum to the first well of the third column and 25 μl of each serum samples from the first well of columns of the plate. From this first dilution of each column transfer 25 μl to the next well and so on until the well No.11 which corresponds to the last dilution, from which 25 μl rule. dilutions 1/2, 1/4, 1/8, 1/16, 1/32, 1/64, 1/128, 1/256 and 1/512, 1/1024, 1/2048 be obtained. Prepare the antigen carrier sheets T. cruzi 15 minutes early to achieve room temperature 20 ° - 25 ° C, label them with the identification number assigned to the samples. Dispense 12 ul of each of the dilutions made of internal controls for assembly and test samples on wells sheets previously labeled antigen, initiating the more diluted (1/2048) to the more concentrated (1/8). Incubate the plates in a humid chamber at 37 ± 1 ° C for 45 minutes horizontally avoiding sudden movements. Fulfilled the incubation time wash each plate with PBS buffer solution pH 7.2 - 7.6, using a wash bottle. Wash the plates by immersion in PBS pH 7.2 buffer solution - 7.6 for 5 minutes by gentle rotation in shaker Mazzini 40 ± 5 rpm. Perform this step twice. Dry the sheets with dry compressed air or alternatively with a fan or free drying. Cover circular areas of the sheets with 12 ul of anti-IgG conjugate diluted in Evans Blue 1: 1000 according to predetermined title. Incubate again the sheets in a humid chamber at 37 ± 1 ° cPer 45 minutes. Wash each plate with PBS buffer solution pH 7.2 - 7.6, using a wash bottle. Wash the plates by immersion in PBS pH 7.2 buffer solution - 7.6 for 5 minutes by gentle rotation in shaker Mazzini 40 ± 5 rpm. Perform twice this step and a final wash with distilled water. Dry the sheets with dry compressed air, or alternatively with a fan or free drying. Add to each well 1 drop of glycerin working solution and completely cover sheet with lamellae 22 x 60 mm. Observe the fluorescence microscope with 40X objective. Considered as IFA test was positive titres When Were ≥1 / 32 (2,3)

**References**

1. Feilij H, Muller L, Gonzalez Cappa SM. Direct micromethod for diagnosis of acute and congenital Chagas’ disease. J Clin Microbiol. 1983 Aug;18(2):327–30.
2. López MC, Duque Beltrán S, Orozco Vargas LC, Camargo D, Gualdrón LE, Cáceres E, et al. Inmunodiagnóstico de la infección chagásica por ELISA. Biomédica, Rev del Inst Nac Salud. 1999;19(2):159–63.
3. Camargo, M.E. Fluorescent antibody test for the diagnosis of American trypanosomiasis. Technical modification employing preserved culture forms of *Trypanosoma cruzi* in a slide test. *Rev. Inst. Med. Trop. São Paulo, 8*:227-234, 1966.
