# Supplementary material for: Molecular Diagnosis of Chagas Disease in Colombia: Parasitic Loads and Discrete Typing Units in Patients from Acute and Chronic Phases
Source: PLoS Negl Trop Dis. 2016 Sep 20;10(9):e0004997. doi: 10.1371/journal.pntd.0004997 (PMC5029947; doi:10.1371/journal.pntd.0004997)
Supplement: S2 Appendix — (DOC) [file pntd.0004997.s004.doc]

### Apendix S2. Detailed methodology of the molecular tools employed in the study

### The procedures were performed following the parameters established by the Clinical & Laboratory Standards Institute (1). To avoid problems of contamination, the laboratory where the samples were processed consisted of 5 specific sub-areas for processing samples 1. Sample Preparation Zone (Mixture of whole blood and guanidine) 2. DNA extraction Zone 3. Pre mix Zone (PCR mixtures) amplification 4. DNA Zone (Add DNA and thermocycling reaction mixture) and 5. Electrophoresis Zone (identifying amplicons).

### 10mL samples of whole blood were collected in EDTA immediately equal volume of 6M guanidine hydrochloride was added and cooled (2-8 ° C) until processing, which was done after 48 hours of collection. In order to avoid contamination guanidine hydrochloride was previously prepared in the premix and there 10 mL aliquots in falcon tubes served, which were mixed with the blood at the site of sampling, this to avoid contamination prior hydrochloride. Serum collection was performed by centrifugal separation of whole blood in dry tube for 10 minutes at 3000 rpm, the sera obtained were frozen (-20 ° C) until processing.

1. **DNA extraction:**

10 mL of Blood samples treated with 10mL Guanidine Hidrochloride 6M, EDTA 0.2 M buffer, pH 8.00 (GEB) from patients were processed using the High Pure PCR Template Preparation kit Roche Diagnostics). Before the extraction, 5 µL of linearized IAC (Internal amplification control) (40 pg/µL) were added to 100 µL of binding solution in a clean tube and 300 µL of GEB, were added and the mix was homogenized. The solution was further mixed with 40 µL of proteinase K by vortexing during 15 sec., spinned down and incubated at 70°C for 10 min. in a dry thermo-block.

After spin down, 100 µL of isopropanol were added, vortexed during 15 sec. and spinned down. Each sample was loaded into an extraction column placed into a 2 mL microtube. The content was centrifuged at 8000 rpm during 1 min. The extraction column was placed into a new collection tube. Inhibitors removing solution (500 µL) was added to each column and centrifuged as described before. The column was placed into a new tube. Washing solution (500 µL) was added to the column and centrifuged as described before. The column was placed into a new tube and the washing step was repeated. The column was placed into a 1.5 mL microtube and centrifuged at maximum speed for 10 sec. One hundred µL of pre-heated elution buffer were added to the column and centrifuged as previously described (In a serum samples were added 50 µL of pre-heated elution buffer). The eluate was stored at 20°C for qPCR and cPCR analysis.

In order to build the standard curves for quantification of parasitic, DNA from 10-fold serial dilutions of blood spiked with *T. cruzi* epimastigotes were obtained. (2,3). To avoid contamination, 12 samples were extracted maximum by each procedure. The negative control (GEB from a seronegative patient and without risk factors) and a reagent control (water) were included.

1. **Conventional PCR**

### Conventional PCR for detection of Trypanosoma cruzi Satellite DNA (cPCR)

### For amplification of the *T. cruzi* nuclear satellite region, the amplification reactions were performed in a total volume of 30µl. This reaction mixture consisted of 1X Taq polymerase amplification buffer (100 mM Tris-HCl, pH 8.3; Invitrogen), 100 mM deoxynucleoside triphosphate solution, 25 mM MgCl2 solution, 5 U/µl of Taq polymerase platinum (Invitrogen), 50 pM of T. cruzi nuclear repetitive region-specific primers cruzi1 (5’ ASTCGGCTGATCGT TTTCGA 3’) and cruzi2 (5' AATTCCTCCAAGCAGCGGATA 3’), 5 µl of template DNA, and a quantity of water sufficient to give a final volume of 30 µl. Optimal cycling conditions were a first step of 10 min. at 94°C for 5 min. followed by 40 cycles of 94°C for 1 min., 64°C fo 30 seg. and 72°C for 1 min. in an automatic thermocycler (Bio Rad S100). The possibility of contamination of the PCR reagents and of the solutions used to prepare DNA was carefully examined through the use of appropriate controls (DNA from strain MHOM/CO/01/DA and DNA from acute patient with blood smear positive, as positive controls and DNA from blood serologically negative without risk factors as negative controls), and each sample was tested in duplicate. Twenty microliters PCR product was analyzed by electrophoresis on a 2% agarose gel and visualized by staining with Gel Red for gel staining (Sigma) (4). The presence of a fragment of 166 bp was interpreted as positive result for *T. cruzi.*

### Conventional PCR for detection of Trypanosoma cruzi kinetoplast (kPCR)

### Only in the positives samples for satellite nuclear PCR, it was performed the kPCR to determinate the presence of *T. cruzi* or *T. rangeli.* For amplification of the *T. cruzi* kinetoplast, the amplification reactions were performed in a total volume of 30µl. This reaction mixture consisted of 1X Taq polymerase amplification buffer (100 mM Tris-HCl, pH 8.3; Invitrogen), 100 mM deoxynucleoside triphosphate solution, 25 mM MgCl2 solution, 5 U/µl of Taq polymerase platinum (Invitrogen), 50 pM of *T. cruzi* minicircle-specific primers 121 (5’ AAATAATGTACGGGKGAGATGCATGA 3’) and 122 (5' GGTTCGATTGGGGTTGGTGTAATATA 3’), 3 µl of template DNA, and a quantity of water sufficient to give a final volume of 21 µl. Optimal cycling conditions were a first step of 10 min. at 94°C for 5 min. followed by 5 cycles of 94°C for 1 min., 68°C for 1 min. and 72°C for 1 min, followed by 35 cycles of 94°C for 1 min., 64°C for 1 min. and 72°C for 1 min and final extension of 72°C for 10 min in an automatic thermocycler (Bio Rad S100). DNA from strains MHOM/CO/01/DA and RHO/CO/82/Durán were used as positives controls of *T. cruzi* and *T. rangeli*, respectively. DNA from blood serologically negative without risk factors as negative controls. Ten microliters PCR product was analyzed by electrophoresis on a 2% agarose gel and visualized by staining with Gel Red for gel staining (Sigma) (4,5). The presence of a fragment of 330 bp was interpreted as positive result for *T. cruzi* while that fragments between 450 and 500 bp were interpreted as positive result for *T. rangeli.*

### Conventional PCR for detection of IAC (Internal amplification control)

### The amplification of IAC was performed in a total volume of 25µl using the master mix GoTaq® Green Master Mix Promega. The reaction mixture consisted in 12,5 µl of GoTaq® Green Master Mix, 10 µM of IAC specific primers IAC-FW (5’ ACCGTCATGGAACAGCACGTA3’) and IAC-Rv (5’ CTCCCGCAACAAACCCTATAAAT 3’), 5 µl of DNA template and water necessary to give a final volume of 25 µl. Optimal cycling conditions were a first step of 10 min. at 94°C for 5 min. followed by 40 cycles of 94°C for 1 min., 60°C for 1 min. and 72°C for 1 min and final extension of 72°C for 5 min in an automatic thermocycler (Bio Rad S100). The presence of a fragment of 199 bp was interpreted as positive result for internal control of cPCR. 5uL of plasmidic DNA (IAC) was used as positive control of PCR.

### Multiplex Real-Time PCR Assay Using TaqMan Probes for Quantification of Trypanosoma cruzi Satellite (qPCR)

### The qPCR reactions were carried out with 5 µL of resuspended DNA, using FastStart Universal Probe Master Mix (Roche Diagnostics) in a final volume of 20 µL, the PCR mix conditions can be observed in Table 1.

**Table 1. The qPCR mix conditions**

| **Reagents** | **[ ] Initial** | **Volume (µL)** | **[ ] Final** |
| --- | --- | --- | --- |
| **Master mix** | 2x | 10 | 1x |
| **Primer cruzi 1** | 10uM | 1.5 | 0.75 |
| **Primer cruzi 2** | 10uM | 1.5 | 0.75 |
| **Primer IACRv** | 10uM | 0.4 | 0.1 |
| **Primer IACFw** | 10uM | 0.4 | 0.1 |
| **Probe Cruzi 3** | 10uM | 0.2 | 0.05 |
| **Probe IACq** | 10uM | 0.2 | 0.05 |
| **Water** |  | 0.8 |  |
| **Final volumen** |  | 15 µL |  |

### *T. cruzi* and IAC primers and probes sequences can be observed in Table 2. Optimal cycling conditions were a first step of 10 min. at 95°C followed by 40 cycles at 95°C for 15 sec. and 58°C for 1 min (Fluorescence detection). The amplifications were carried out in a Applied Biosystems (ABI 7500, USA) device.

### Standard curves were constructed with 1/10 serial dilutions of total DNA obtained from a GEB sample spiked with 10^6^ parasite equivalents/mL of blood until 10^-1^ parasites equivalents/mL. TcI DTU strain (MHOM/CO/01/DA) was used for standard curve (2). The results were considered positives and were performed quantification when the amplification exceeded the threshold of fluorescence 0.01 and the dynamic range for quantification was 0.1 to 1.000.000 parasites equivalents/mL. The negative results were confirmed when the Ct for IAC was between 19 and 28. DNA from blood serologically negative without risk factors was used as negative controls and DNA from acute patient with blood smear positive. All samples were performed for duplicate.

**Table 2. *T. cruzi* and IAC primers and probes**

| **Pimers and probes** | **Sequence** |
| --- | --- |
| **Cruzi 1 (Forward)** | ASTCGGCTGATCGTTTTC |
| **Cruzi 2 (Reverse)** | AATTCCTCCAAGCAGCGGATA |
| **Cruzi 3 (probe)** | FAM-CACACACTGGACACCAA-NFQ-MGB |
| **IACRv** | CTCCCGCAACAAACCCTATAAAT |
| **IACFw** | ACCGTCATGGAACAGCACGTA |
| **IAC (probe)** | VIC-AGCATCTGTTCTTGAAGGT-NFQ-MGB |

1. **Genotyping Techniques**

In samples positive for T. cruzi molecular diagnostics, PCR techniques were applied to amplify five molecular markers : two regions of the intergenic miniexon - gene (SL- IR), ribosomal DNA 24Sα , 18S ribosomal DNA and the fragment A10, widely used for discrimination in its six T. cruzi DTUs . Green Master Mix the GoTaq® Promega was used for amplification of these 5 targets. In all reactions there were added 12.5 uL of master mix GoTaq® Green , 10 uM of each primer, and 5 uL of DNA sufficient to complete 25 uL final reaction with water. The primers and thermal profiles for each target are described below

- **(SL-IR)**

| **Primer** | **Sequence** |
| --- | --- |
| **TCC** | 5´- CCCCCCTCCCAGGCCACACTG-´3 |
| **TC1M** | 5´- GTGTCCGCCACCTCCTTCGGGCC-´3 |
| **TC2** | 5´- CCTGCAGGCACACGTGTGTGTG-´3 |

| **Thermal profile SL-IR** | | |
| --- | --- | --- |
| **Temperature** | | **Time** |
| **95°C** | | 5 min |
| **5 CYCLES** | | |
| **94°C**  **67°C**  **72°C** | | 1 min  1 min  1 min |
| **5 CYCLES** | | |
| **94°C**  **65°C**  **72°C** | | 1 min  1 min  1 min |
| **5 CYCLES** | | |
| **94°C**  **63°C**  **72°C** | 1 min  1 min  1 min | |
| **30 CYCLES** | | |
| **94°C**  **61°C**  **72°C** | | 1 min  1 min  1 min |
| **FINAL EXTENSION** | | |
| **72°C** | 10 min | |

- **24Sα**

| **Primer** | **Sequence** |
| --- | --- |
| **D71** | 5´-CCCCCCTCCCAGGCCACACTG -´3 |
| **D72** | 5´- GTGTCCGCCACCTCCTTCGGGCC -´3 |

- **18s**

| **Primers** | **Sequence** |
| --- | --- |
| **V1** | 5´- CAAGCGGCTGGGTGGTTATTCCA -´3 |
| **V2** | 5´- TTGAGGGAAGGCATGACACATGT -´3 |

- **Fragment A10**

| **Primer** | **Sequence** |
| --- | --- |
| **Pr1** | 5´- CCGCTAAGCAGTTCTGTCCATA -´3 |
| **Pr3** | 5´- TGCTTTATTACCCCATGCCACAG-´3 |

| **THERMAL PROFILE 24Sα, 18s y A10** | | |
| --- | --- | --- |
| **Temperature** | | **Time** |
| **94°C** | | 5 minutes |
| **30 cycles** | | |
| **94 °C** | | 1 minute |
| **60°C** | | 30 seconds |
| **72°C** | | 1 minute |
| **1 cycle** | | |
| **72°C** | 5 minutes | |
| **4°C** | Infinite | |

To classify the DTU TcI in TcIDom and TcISylvatic genotypes, the reaction mixture consisted of 1X Taq polymerase amplification buffer (100 mM Tris-HCl, pH 8.3; Invitrogen), 100 mM deoxynucleoside triphosphate solution, 25 mM MgCl2 solution, 5 U / .mu.l of Taq polymerase platinum (Invitrogen), 50 pM of *T. cruzi* mini-exon primers 1am (5'TGTGTGTGTATGTATGTG 3 ') and 1B (5' CGGAGCGGTGTGTGCAG 3 '). The thermal profile consisted of initial denaturation of 94 ° C for 4 min, followed by 35 cycles of 94 ° for 30 sec, 55 ° C for 20 sec and 72 ° C for 20 sec and finally final extension of 72 ° C for 10 min (6,7).

Based on the sizes of amplicons in each PCR according to the algorithm (Figure S1.) DTU genotyping in each of the samples (8-14,6,7) was performed. As positive controls 7 strains were used: MHOM / CO / 01 / DA (TcIDom), MHOM / CO / 10 / GC (TcISylvatic), TcII (Y), TcIII (CM17), TcIV (YLY), TcV (V) and TcVI (CLBrener) previously genotyped using various molecular markers and sequencing.

**References**

1. Clinical and Laboratory Standards Institute. CLSI. Quantitative Molecular Methods for Infectious Diseases ; Approved Guideline — Second Edition. 2010 p. 80.

2. Duffy T, Cura CI, Ramirez JC, Abate T, Cayo NM, Parrado R, et al. Analytical Performance of a Multiplex Real-Time PCR Assay Using TaqMan Probes for Quantification of Trypanosoma cruzi Satellite DNA in Blood Samples. PLoS Negl Trop Dis. 2013;7(1).

3. Melo MF, Moreira OC, Tenório P, Lorena V, Lorena-Rezende I, Júnior WO, et al. Usefulness of real time PCR to quantify parasite load in serum samples from chronic Chagas disease patients. Parasit Vectors. 2015;8:154.

4. Ramírez JD, Guhl F, Umezawa ES, Morillo C a., Rosas F, Marin-Neto J a., et al. Evaluation of adult chronic Chagas’ heart disease diagnosis by molecular and serological methods. J Clin Microbiol. 2009 Dec;47(12):3945–51.

5. Guhl F, Jaramillo C, Carranza JC, Vallejo G a., Article R. Molecular Characterization and Diagnosis of Trypanosoma cruzi and T . rangeli. Arch Med Res. 2002;33(4976):362–70.

6. Villa LM, Guhl F, Zabala D, Ramírez JD, Urrea DA, Hernández DC, et al. The identification of two Trypanosoma cruzi I genotypes from domestic and sylvatic transmission cycles in Colombia based on a single polymerase chain reaction amplification of the spliced-leader intergenic region. Mem Inst Oswaldo Cruz. 2013;108(7):932–5.

7. León CM, Hernández C, Montilla M, Ramírez JD. Retrospective distribution of Trypanosoma cruzi I genotypes in Colombia. 2015;110(May):387–93.

8. Brisse S, Verhoef J, Tibayrenc M. Characterisation of large and small subunit rRNA and mini-exon genes further supports the distinction of six Trypanosoma cruzi lineages. Int J Parasitol. 2001;31(11):1218–26.

9. Ramírez JD, Guhl F, Rendón LM, Rosas F, Marin-Neto JA, Morillo CA. Chagas cardiomyopathy manifestations and trypanosoma cruzi genotypes circulating in chronic chagasic patients. PLoS Negl Trop Dis. 2010;4(11):1–9.

10. Burgos JM, Diez M, Vigliano C, Bisio M, Risso M, Duffy T, et al. Molecular Identification of *Trypanosoma cruzi* Discrete Typing Units in End‐Stage Chronic Chagas Heart Disease and Reactivation after Heart Transplantation. Clin Infect Dis. 2010;51(5):485–95.

11. Cruz L, Vivas A, Montilla M, Hernández C, Flórez C, Parra E, et al. Comparative study of the biological properties of Trypanosoma cruzi I genotypes in a murine experimental model. Infect Genet Evol [Internet]. Elsevier B.V.; 2015;29:110–7. Available from: http://dx.doi.org/10.1016/j.meegid.2014.11.012

12. Cura CI, Duffy T, Lucero RH, Bisio M, Péneau J, Jimenez-Coello M, et al. Multiplex Real-Time PCR Assay Using TaqMan Probes for the Identification of Trypanosoma cruzi DTUs in Biological and Clinical Samples. PLoS Negl Trop Dis [Internet]. 2015;9(5):e0003765. Available from: http://dx.plos.org/10.1371/journal.pntd.0003765

13. Souto RPP, Fernandes O, Macedo AMM, Campbell D a. A, Zingales B. DNA markers define two major phylogenetic lineages of Trypanosoma cruzi. Mol Biochem Parasitol. 1996;83(2):141–52.

14. Liarte DB, Murta SMF, Steindel M, Romanha AJ. Experimental Parasitology Trypanosoma cruzi : Multiplex PCR to detect and classify strains according to groups I and II q. Exp Parasitol [Internet]. Elsevier Inc.; 2009;123(4):283–91. Available from: http://dx.doi.org/10.1016/j.exppara.2008.12.005
