# Supplementary material for: Molecular Diagnosis of Chagas Disease in Colombia: Parasitic Loads and Discrete Typing Units in Patients from Acute and Chronic Phases
Source: PLoS Negl Trop Dis. 2016 Sep 20;10(9):e0004997. doi: 10.1371/journal.pntd.0004997 (PMC5029947; doi:10.1371/journal.pntd.0004997)
Supplement: S3 Appendix — (DOC) [file pntd.0004997.s005.doc]

**Appendix S3. Sensitivity and specificity calculations**

1. **qPCR Acute phase**

|  |  | ***Gold standard*** | |  |
| --- | --- | --- | --- | --- |
|  | **Results** | Positive | Negative | **Total** |
| **qPCR** | Positive | 68 | 0 | **68** |
|  | Negative | 3 | 15 | **18** |
|  | **Total** | 71 | 15 | **86** |

**Sensitivity:** (68*100) / 71 = 95.7%

**Specificity:** (15*100) / 15 = 100.0%

1. **cPCR Acute Phase**

|  |  | ***Gold standard*** | |  |
| --- | --- | --- | --- | --- |
|  | **Results** | Positive | Negative | **Total** |
| **cPCR** | Positive | 60 | 0 | **60** |
|  | Negative | 11 | 15 | **26** |
|  | **Total** | 71 | 15 | **86** |

**Sensitivity:** (60*100) / 71 = 84.5%

**Specificity:** (15*100) / 15 = 100.0%

1. **qPCR Chronic phase (Negatives without risk factors)**

|  |  | ***Gold standard*** | |  |
| --- | --- | --- | --- | --- |
|  | **Results** | Positive | Negative | **Total** |
| **qPCR** | Positive | 309 | 0 | **309** |
|  | Negative***** | 172 | 29 | **201** |
|  | **Total** | 481 | 29 | **510** |

*****Negatives without risk factors

**Sensitivity:** (309*100) /481 = 64.2%

**Specificity:** (29*100) / 29 = 100.0%

1. **cPCR Chronic phase (Negatives without risk factors)**

|  |  | ***Gold standard*** | |  |
| --- | --- | --- | --- | --- |
|  | **Results** | Positive | Negative | **Total** |
| **cPCR** | Positive | 273 | 0 | **273** |
|  | Negative***** | 208 | 29 | **237** |
|  | **Total** | 481 | 29 | **510** |

*****Negatives without risk factors

**Sensitivity:** (273*100) /481 = 56.8%

**Specificity:** (29*100) / 29 = 100.0%

1. **qPCR Chronic indeterminate phase (Negatives without risk factors)**

|  |  | ***Gold standard*** | |  |
| --- | --- | --- | --- | --- |
|  | **Results** | Positive | Negative | **Total** |
| **qPCR** | Positive | 189 | 0 | **189** |
|  | Negative***** | 89 | 29 | **118** |
|  | **Total** | 278 | 29 | **307** |

*****Negatives without risk factors

**Sensitivity:** (189*100) /278 = 68.0%

**Specificity:** (29*100) / 29 = 100.0%

1. **cPCR Chronic indeterminate phase (Negatives without risk factors)**

|  |  | ***Gold standard*** | |  |
| --- | --- | --- | --- | --- |
|  | **Results** | Positive | Negative | **Total** |
| **cPCR** | Positive | 154 | 0 | **154** |
|  | Negative***** | 124 | 29 | **153** |
|  | **Total** | 278 | 29 | **3017** |

*****Negatives without risk factors

**Sensitivity:** (154*100) /278 = 55.4%

**Specificity:** (29*100) / 29 = 100.0%

1. **qPCR Chronic determinate phase (Negatives without risk factors)**

|  |  | ***Gold standard*** | |  |
| --- | --- | --- | --- | --- |
|  | **Results** | Positive | Negative | **Total** |
| **qPCR** | Positive | 120 | 0 | **120** |
|  | Negative***** | 83 | 29 | **112** |
|  | **Total** | 203 | 29 | **232** |

*****Negatives without risk factors

**Sensitivity:** (120*100) /203 = 59.1%

**Specificity:** (29*100) / 29 = 100.0%

1. **cPCR Chronic determinate phase (Negatives without risk factors)**

|  |  | ***Gold standard*** | |  |
| --- | --- | --- | --- | --- |
|  | **Results** | Positive | Negative | **Total** |
| **cPCR** | Positive | 119 | 0 | **119** |
|  | Negative***** | 84 | 29 | **113** |
|  | **Total** | 203 | 29 | **232** |

*****Negatives without risk factors

**Sensitivity:** (119*100) /203 = 58.6%

1. **qPCR Chronic phase (All Negatives: Negatives with risk factors + Negatives without risk factors)**

|  |  | ***Gold standard*** | |  |
| --- | --- | --- | --- | --- |
|  | **Results** | Positive | Negative | **Total** |
| **qPCR** | Positive | 309 | 4 | **313** |
|  | Negative | 172 | 137 | **309** |
|  | **Total** | 481 | 141 | **622** |

**Sensitivity:** (309*100) /481 = 64.2%

**Specificity:** (137*100) / 141 = 97.2%

1. cPCR Chronic phase (All Negatives: Negatives with risk factors + Negatives without risk factors)

|  |  | ***Gold standard*** | |  |
| --- | --- | --- | --- | --- |
|  | **Results** | Positive | Negative | **Total** |
| **cPCR** | Positive | 273 | 3 | **276** |
|  | Negative | 208 | 138 | **346** |
|  | **Total** | 481 | 141 | **622** |

**Sensitivity:** (273*100) /481 = 56.8%

**Specificity:** (138*100) /141 = 97.9%

1. **qPCR Chronic indeterminate phase (All Negatives: Negatives with risk factors + Negatives without risk factors)**

|  |  | ***Gold standard*** | |  |
| --- | --- | --- | --- | --- |
|  | **Results** | Positive | Negative | **Total** |
| **qPCR** | Positive | 189 | 4 | **193** |
|  | Negative | 89 | 137 | **226** |
|  | **Total** | 278 | 141 | **419** |

**Sensitivity:** (189*100) /278 = 68.0%

**Specificity:** (137*100) / 141 = 97.2%

1. **cPCR Chronic indeterminate phase (All Negatives: Negatives with risk factors + Negatives without risk factors)**

|  |  | ***Gold standard*** | |  |
| --- | --- | --- | --- | --- |
|  | **Results** | Positive | Negative | **Total** |
| **cPCR** | Positive | 154 | 3 | **157** |
|  | Negative | 124 | 138 | **262** |
|  | **Total** | 278 | 141 | **419** |

**Sensitivity:** (154*100) /278 = 55.4%

**Specificity:** (138*100) /141 = 97.9%

1. **qPCR Chronic determinate phase (All Negatives: Negatives with risk factors + Negatives without risk factors)**

|  |  | ***Gold standard*** | |  |
| --- | --- | --- | --- | --- |
|  | **Results** | Positive | Negative | **Total** |
| **qPCR** | Positive | 120 | 4 | **124** |
|  | Negative | 83 | 137 | **220** |
|  | **Total** | 203 | 141 | **344** |

**Sensitivity:** (120*100) /203 = 59.1%

**Specificity:** (137*100) / 141 = 97.2%

1. **cPCR Chronic determinate phase** (All Negatives: Negatives with risk factors + Negatives without risk factors)

|  |  | ***Gold standard*** | |  |
| --- | --- | --- | --- | --- |
|  | **Results** | Positive | Negative | **Total** |
| **cPCR** | Positive | 119 | 3 | **122** |
|  | Negative | 84 | 138 | **222** |
|  | **Total** | 203 | 141 | **344** |

**Sensitivity:** (119*100) /203 = 58.6%

**Specificity:** (138*100) /141 = 97.9%
