# Supplementary material for: Molecular Diagnosis of Chagas Disease in Colombia: Parasitic Loads and Discrete Typing Units in Patients from Acute and Chronic Phases
Source: PLoS Negl Trop Dis. 2016 Sep 20;10(9):e0004997. doi: 10.1371/journal.pntd.0004997 (PMC5029947; doi:10.1371/journal.pntd.0004997)
Supplement: S4 Appendix — (DOCX) [file pntd.0004997.s006.docx]

|  | **Section & Topic** | **No** | **Item** | **Reported on page #** |
| --- | --- | --- | --- | --- |
|  |  |  |  |  |
|  | **TITLE OR ABSTRACT** |  |  |  |
|  |  | **1** | Identification as a study of diagnostic accuracy using at least one measure of accuracy  (such as sensitivity, specificity, predictive values, or AUC) | 2-3 |
|  | **ABSTRACT** |  |  |  |
|  |  | **2** | Structured summary of study design, methods, results, and conclusions  (for specific guidance, see STARD for Abstracts) | 2-3 |
|  | **INTRODUCTION** |  |  |  |
|  |  | **3** | Scientific and clinical background, including the intended use and clinical role of the index test | 4-5 |
|  |  | **4** | Study objectives and hypotheses | 5 |
|  | **METHODS** |  |  |  |
|  | *Study design* | **5** | Whether data collection was planned before the index test and reference standard  were performed (prospective study) or after (retrospective study) | 7 |
|  | *Participants* | **6** | Eligibility criteria | 6 |
|  |  | **7** | On what basis potentially eligible participants were identified  (such as symptoms, results from previous tests, inclusion in registry) | 7-8 |
|  |  | **8** | Where and when potentially eligible participants were identified (setting, location and dates) | 7 |
|  |  | **9** | Whether participants formed a consecutive, random or convenience series | 7-8 |
|  | *Test methods* | **10a** | Index test, in sufficient detail to allow replication | Page 8-9  Apendix S2. |
|  |  | **10b** | Reference standard, in sufficient detail to allow replication | Appendix S1. |
|  |  | **11** | Rationale for choosing the reference standard (if alternatives exist) | NA |
|  |  | **12a** | Definition of and rationale for test positivity cut-offs or result categories of the index test, distinguishing pre-specified from exploratory | 9  Appendix S2. |
|  |  | **12b** | Definition of and rationale for test positivity cut-offs or result categories of the reference standard, distinguishing pre-specified from exploratory | 8-9  Appendix S2. |
|  |  | **13a** | Whether clinical information and reference standard results were available  to the performers/readers of the index test | 9 |
|  |  | **13b** | Whether clinical information and index test results were available  to the assessors of the reference standard | 8-9 |
|  | *Analysis* | **14** | Methods for estimating or comparing measures of diagnostic accuracy | 10 |
|  |  | **15** | How indeterminate index test or reference standard results were handled | 9 |
|  |  | **16** | How missing data on the index test and reference standard were handled | 6 |
|  |  | **17** | Any analyses of variability in diagnostic accuracy, distinguishing pre-specified from exploratory | NA |
|  |  | **18** | Intended sample size and how it was determined | 6-7 |
|  | **RESULTS** |  |  |  |
|  | *Participants* | **19** | Flow of participants, using a diagram | 10-11  Figure 1. |
|  |  | **20** | Baseline demographic and clinical characteristics of participants | 10-11  Table 1. |
|  |  | **21a** | Distribution of severity of disease in those with the target condition | 11 |
|  |  | **21b** | Distribution of alternative diagnoses in those without the target condition | Notes Table 1. |
|  |  | **22** | Time interval and any clinical interventions between index test and reference standard | 10-11 |
|  | *Test results* | **23** | Cross tabulation of the index test results (or their distribution)  by the results of the reference standard | Appendix S3. |
|  |  | **24** | Estimates of diagnostic accuracy and their precision (such as 95% confidence intervals) | Tables 2,3,4, 5. |
|  |  | **25** | Any adverse events from performing the index test or the reference standard | NA |
|  | **DISCUSSION** |  |  |  |
|  |  | **26** | Study limitations, including sources of potential bias, statistical uncertainty, and generalisability | 17, 19-21 |
|  |  | **27** | Implications for practice, including the intended use and clinical role of the index test | 17-18,21 |
|  | **OTHER INFORMATION** |  |  |  |
|  |  | **28** | Registration number and name of registry | 6 |
|  |  | **29** | Where the full study protocol can be accessed | 6 |
|  |  | **30** | Sources of funding and other support; role of funders | 26 |
|  |  |  |  |  |

STARD 2015

### AIM

STARD stands for “Standards for Reporting Diagnostic accuracy studies”. This list of items was developed to contribute to the completeness and transparency of reporting of diagnostic accuracy studies. Authors can use the list to write informative study reports. Editors and peer-reviewers can use it to evaluate whether the information has been included in manuscripts submitted for publication.

### Explanation

A **diagnostic accuracy study** evaluates the ability of one or more medical tests to correctly classify study participants as having a **target condition.** This can be a disease, a disease stage, response or benefit from therapy, or an event or condition in the future. A medical test can be an imaging procedure, a laboratory test, elements from history and physical examination, a combination of these, or any other method for collecting information about the current health status of a patient.

The test whose accuracy is evaluated is called **index test.** A study can evaluate the accuracy of one or more index tests. Evaluating the ability of a medical test to correctly classify patients is typically done by comparing the distribution of the index test results with those of the **reference standard**. The reference standard is the best available method for establishing the presence or absence of the target condition. An accuracy study can rely on one or more reference standards.

If test results are categorized as either positive or negative, the cross tabulation of the index test results against those of the reference standard can be used to estimate the **sensitivity** of the index test (the proportion of participants *with* the target condition who have a positive index test), and its **specificity** (the proportion *without* the target condition who have a negative index test). From this cross tabulation (sometimes referred to as the contingency or “2x2” table), several other accuracy statistics can be estimated, such as the positive and negative **predictive values** of the test. Confidence intervals around estimates of accuracy can then be calculated to quantify the statistical **precision** of the measurements.

If the index test results can take more than two values, categorization of test results as positive or negative requires a **test positivity cut-off**. When multiple such cut-offs can be defined, authors can report a receiver operating characteristic (ROC) curve which graphically represents the combination of sensitivity and specificity for each possible test positivity cut-off. The **area under the ROC curve** informs in a single numerical value about the overall diagnostic accuracy of the index test.

The **intended use** of a medical test can be diagnosis, screening, staging, monitoring, surveillance, prediction or prognosis. The **clinical role** of a test explains its position relative to existing tests in the clinical pathway. A replacement test, for example, replaces an existing test. A triage test is used before an existing test; an add-on test is used after an existing test.

Besides diagnostic accuracy, several other outcomes and statistics may be relevant in the evaluation of medical tests. Medical tests can also be used to classify patients for purposes other than diagnosis, such as staging or prognosis. The STARD list was not explicitly developed for these other outcomes, statistics, and study types, although most STARD items would still apply.

### DEVELOPMENT

This STARD list was released in 2015. The 30 items were identified by an international expert group of methodologists, researchers, and editors. The guiding principle in the development of STARD was to select items that, when reported, would help readers to judge the potential for bias in the study, to appraise the applicability of the study findings and the validity of conclusions and recommendations. The list represents an update of the first version, which was published in 2003.

More information can be found on [http://www.equator-network.org/reporting-guidelines/stard](http://www.equator-network.org/reporting-guidelines/stard/).

**TITLE OR ABSTRACT**

1. Identification as a study of diagnostic accuracy using at least one measure of accuracy (such as sensitivity, specificity, predictive values, or AUC)

- Lines 22-24

The objective of this study was to determine the operating characteristics of molecular tests (conventional and Real Time PCR) for the detection of *T. cruzi* DNA, parasitic loads and DTUs in a large cohort of Colombian patients from acute and chronic phases

- Lines 29-32

The operating characteristics showed that performance of qPCR was higher compared to cPCR. Likewise, the performance of qPCR was significantly higher in acute phase compared with chronic phase.

- Lines 38-40

The molecular tests are a precise tool to complement the standard diagnosis of Chagas disease, specifically in acute phase showing high discriminative power. However, it is necessary to improve the sensitivity of molecular tests in chronic phase.

**ABSTRACT**

1. **Structured summary of study design, methods, results, and conclusions (for specific guidance, see STARD for Abstracts)**

**INTRODUCTION**

1. Scientific and clinical background, including the intended use and clinical role of the index test

- Lines 62-69:

Chagas disease is a zoonotic parasitic disease caused by the protozoan *Trypanosoma cruzi.* It is considered a public health problem in Latin-America, where approximately 6 million people are currently infected (1). The acute phase of the disease is characterised by usually mild fever that in a small proportion of cases can be accompanied by myocarditis and other lethal complications. Most of the patients continue through the chronic phase that is initially characterised by an asymptomatic clinical course during two or three decades, and about 30% of the infected patients will develop heart or digestive complications afterwards (2).

- Lines 80-97

The diagnosis of Chagas disease is complex due to the dynamics of parasitemia in the phases of the disease. During the acute phase the parasitemia is high, therefore the diagnosis is performed by direct parasitological tests (15,16). Nevertheless, direct parasitological tests are not useful in the chronic phase due to the low and intermittent parasitemias. Therefore, the diagnosis of Chagas disease in the chronic phase is determined by serological tests such as ELISA: enzyme-linked immunosorbent assay, IFA: indirect immunofluorescence assay or HAI: Hemagglutination Inhibition Test (17–19). Recently, molecular techniques such as cPCR (conventional PCR) and qPCR (quantitative real-time PCR) have been considered as supportive diagnostic tests due to their ability to determine parasitic loads of *T. cruzi* in all clinical phases of the disease (20–22). The operating characteristics of molecular tests for diagnosis of *T. cruzi* infection have varied according to clinical phase and technical specifications. Sensitivity for identifying chronic infection with cPCR has ranged between 22 and 75% (23,24) and in both cases with a specificity of 100%. Contrastingly, for qPCR, sensitivity has ranged between 60 and 80% (22,25,26) in chronic phase and between 88% and 100% for acute phase (25,26), whereas specificity is between 70-100% (26–28). Sampling methods have not been always clearly stated and the role of these techniques for diagnosis of Chagas disease in the different clinical phases still remains poorly understood.

1. Study objectives and hypotheses

- Lines 99-104

The objective of this work was to determine the operating characteristics of qPCR and cPCR targeting the satellite nuclear DNA region, compared with standard diagnosis methods for acute and chronic Chagas disease. Additionally, we evaluated the plausible associations between parasitic load and DTUs in Colombian patients from the acute and chronic phases to untangle the natural course of *T. cruzi* infection in terms of parasite dynamics.

**METHODS**

*Study design*

1. Whether data collection was planned before the index test and reference standard
   were performed (prospective study) or after (retrospective study)

- Lines 140-143 :

The inclusion of participants was conducted retrospectively for the period 2004 to 2012, and prospectively between 2013 and 2015. At the end, a total of 86 suspected acute patients and 622 suspected chronic patients were included in the study (Table 1).

*Participants*

1. Eligibility criteria

-Lines 109-116:

All patients who attended the Colombian National Health Institute (Overall 985 individuals) seeking diagnostic tests for Chagas disease in acute (113 patients) or chronic phase (872 patients) between 2004 and 2015 were considered as potential participants.

Inclusion criteria were: i. Clinical or epidemiological suspect of Chagas disease in acute or chronic phase ii. Not having received aetiological treatment for Chagas disease iii. Positive serological tests for Chagas disease (IFA, ELISA and/or HAI) iv. Adequate blood and serum samples available for performing diagnostic tests according to the clinical phase. v. Acceptance to participate and sign the informed consent.

1. On what basis potentially eligible participants were identified (such as symptoms, results from previous tests, inclusion in registry)

- Lines 138-140:

Clinical evaluation was conducted simultaneously to all individuals as part of the study to determine health status and then to the conformed cases to evaluate heart complications.

- Lines 145-164

**Clinical classification**

**Acute phase:** a suspected case was defined as an individual with > 7 days of fever accompanied or not by hepatomegaly or splenomegaly. The patient was considered with acute Chagas disease if additionally to symptoms tested positive by parasitological tests (Strout, micro-strout, blood thick smear, or hemoculture) (15) or presented positive results to two serological tests over the course of the following weeks (30,31). The patients were classified as negative to Chagas disease otherwise noted.

**Chronic phase:** individuals without criteria for acute phase but with clinical or epidemiological suspicion of Chagas disease. The patients were confirmed as positive *T. cruzi* infection when tested positive to two serological tests (IFA, ELISA and/or HAI). It was then classified as chronic indeterminate (when no evidence of signs or symptoms of heart complications were evinced) or chronic determined otherwise.

The risk factors classification was conducted through a survey applied to each of the patients included in the study. A series of questions were asked such as the place of birth, knowledge of vector insects, blood donations and/or organ transplantation, housing type and presence of cardiac symptoms based on previous evaluated questionnaires (32). Patients whose serological tests were negative were classified into two groups according to the presence or absence of risk factors. The patients, who had one or more risk factors, were categorized as "negative with risk factors" and those patients that did not have any risk factors were categorized as "negative without risk factors".

1. Where and when potentially eligible participants were identified (setting, location and dates)

- Lines 109-116:

All patients who attended the Colombian National Health Institute (Overall 985 individuals) seeking diagnostic tests for Chagas disease in acute (113 patients) or chronic phase (872 patients) between 2004 and 2015 were considered as potential participants

1. Whether participants formed a consecutive, random or convenience series

- Lines 109-111:

All patients who attended the Colombian National Health Institute (Overall 985 individuals) seeking diagnostic tests for Chagas disease in acute (113 patients) or chronic phase (872 patients) between 2004 and 2015 were considered as potential participants.

- Lines 145-164

**Clinical classification**

**Acute phase:** a suspected case was defined as an individual with > 7 days of fever accompanied or not by hepatomegaly or splenomegaly. The patient was considered with acute Chagas disease if additionally to symptoms tested positive by parasitological tests (Strout, micro-strout, blood thick smear, or hemoculture) (15) or presented positive results to two serological tests over the course of the following weeks (30,31). The patients were classified as negative to Chagas disease otherwise noted.

**Chronic phase:** individuals without criteria for acute phase but with clinical or epidemiological suspicion of Chagas disease. The patients were confirmed as positive *T. cruzi* infection when tested positive to two serological tests (IFA, ELISA and/or HAI). It was then classified as chronic indeterminate (when no evidence of signs or symptoms of heart complications were evinced) or chronic determined otherwise.

The risk factors classification was conducted through a survey applied to each of the patients included in the study. A series of questions were asked such as the place of birth, knowledge of vector insects, blood donations and/or organ transplantation, housing type and presence of cardiac symptoms based on previous evaluated questionnaires (32). Patients whose serological tests were negative were classified into two groups according to the presence or absence of risk factors. The patients, who had one or more risk factors, were categorized as "negative with risk factors" and those patients that did not have any risk factors were categorized as "negative without risk factors".

*Test methods*

1. Index test, in sufficient detail to allow replication

The information about Index Test (cPCR and qPCR) is in papper: Lines 183-195 and Appendix S2.

1. Reference standard, in sufficient detail to allow replication

The information about reference standard (serology and direct methods) is in paper: Lines 173-181 and Appendix S1.

1. Rationale for choosing the reference standard (if alternatives exist)

NA

1. Definition of and rationale for test positivity cut-offs or result categories
   of the index test, distinguishing pre-specified from exploratory

- Paper Lines 190-192:

The qPCR test was considered positive when the amplification exceeded the threshold of fluorescence 0.01 and cPCR when was observed a DNA fragment of 166 bp in the electrophoresis

**Appendix S2.**

**qPCR:** “The results were considered positives and were performed quantification when the amplification exceeded the threshold of fluorescence 0.01 and the dynamic range for quantification was 0.1 to 1.000.000 parasites equivalents/mL**.**

**cPCR:** “Tthe presence of a fragment of 166 bp was interpreted as positive result for *T. cruzi.* “

1. Definition of and rationale for test positivity cut-offs or result categories of the reference standard, distinguishing pre-specified from exploratory

- Paper Lines 170-171:

The results were considered positive when morphology compatible with the *T.cruzi* was observed.

- Paper Lines 175-176:

All serological tests were conducted in duplicate and positive and negative controls were used for each assay. ELISA test was considered as positive when absorbance was ≥0.300, IFA when titres were ≥1/32 and HAI when titres were ≥1/32.

- Appendix S1 :

1. Whether clinical information and reference standard results were available to the performers/readers of the index test

- Lines 194-195: All samples were analysed without knowledge of the clinical status or other tests

1. Whether clinical information and index test results were available to the assessors of the reference standard

- Lines 171-172 and 178-179:

All samples were analysed without knowledge of the clinical status or other tests

*Analysis*

1. Methods for estimating or comparing measures of diagnostic accuracy

-Lines 201-205:

Operating characteristics of the molecular tests were estimated by comparing against standard diagnosis (described above). Sensitivity, specificity, positive (+LR) and negative likelihood ratio (LR-), predictive values (PV), diagnostic precision (DP), Area under the curve (AUC), and Kappa index (K) were estimated for each phase of the disease (acute and chronic), the clinical stage of chronic patients (determined and indeterminate) and according to DTUs and TcI genotypes identified (TcI-sylv/TcIDom).

- Appendix S3. Sensitivity and specificity calculations

1. How indeterminate index test or reference standard results were handled

-Lines 179-181:

The indeterminate results in the serology tests (ELISA and IFI) were resolved by use of HAI test.

1. How missing data on the index test and reference standard were handled

- Lines 114-115:

An inclusion criteria was: adequate blood and serum samples available for performing diagnostic tests according to clinical phase.

- Lines 220-223:

Out of the initial potential participants, 27 and 129 were excluded for incomplete samples to perform all analysis from the acute and chronic groups, respectively and 121 from the chronic group due to absence of clinical information.

1. Any analyses of variability in diagnostic accuracy, distinguishing pre-specified from exploratory
2. Intended sample size and how it was determined

- Lines 125-137:

The total sample size (N) was calculated for test binary outcomes and separately for each clinical phase: acute and chronic. Considering, n=Z^2^ S (1−S) *d*^2^, where for a confidence level of 95% (1- α, with α= 0.05) Z is inserted by 1.96, and a maximum marginal error of estimate, *d*, is a desired value for precision based on researchers judgment, and S is a pre-determined value of sensitivity (29). Based in previous studies, for the acute phase S was pre-established at 92% and with *d* at 8% (25,26), whereas for chronic phase S was pre-established at 60% with *d* at 5% (22–26). Then, N= n /P, being P the estimated prevalence in this specific population under study. Given this is a selected population, composed of patients with some suspicion of the infection and remitted to a reference centre, P was specified at 60% in suspected cases for both acute and chronic phases. This value was obtained as an approximation based on the laboratory records at the NHI (Bogota, Colombia). The minimum total sample sizes were then calculated as N=74 and N=615 for suspected cases in acute and chronic phases respectively.

**RESULTS**

1. Flow of participants, using a diagram

- Lines 219-223:

Overall, 985 patients were included, 872 suspected of chronic and 113 of acute infection. General demographic characteristics are shown in Table 1. Out of the initial potential participants, 27 and 129 were excluded for incomplete samples to perform all analysis from the acute and chronic groups, respectively and 121 from the chronic group due to absence of clinical information (Figure 1).

**Figure 1. Algorithm for selection and classification of patients.** There were selected 708 patients, 71 in acute phase, 15 febrile negatives, 481 in chronic phase and 141 negatives. *FR: Risk Factor

1. Baseline demographic and clinical characteristics of participants

Overall, 985 patients were included, 872 suspected of chronic and 113 of acute infection. General demographic characteristics are shown in Table 1. Out of the initial potential participants, 27 and 129 were excluded for incomplete samples to perform all analysis from the acute and chronic groups, respectively and 121 from the chronic group due to absence of clinical information.

**Table 1. General characteristics of patients included in the study**

| **General characteristics** | | **Acute phase^b^**  **N= 86** | | **Chronic phase^c^**  **N=622** | |
| --- | --- | --- | --- | --- | --- |
|  |  | **Positive** | **Negative** | **Positive** | **Negative^d^** |
| **Patients number (N)** | 708 | 71 | 15 | 481 | 141 |
| **Age, median (Q1-Q3)^a^** | 48 (47-49) | 31 (26-35) | 27 (23-30) | 51 (50-53) | 37 (39-41) |
| **Sex, n (%)** |  |  |  |  |  |
| **Female** | 428 (60.4) | 26 (36.6) | 8 (53.3) | 313 (65.1) | 60 (42.5) |
| **Male** | 280 (39.6) | 45 (63.4) | 7 (46.7) | 168 (34.9) | 81 (52.4) |

**^a^**Age in years

^b^ Positive patients were those who had positive direct parasitological tests, symptomatology and/or serological tests. Negative patients comprise a group of febrile patients with negative serology for Chagas disease and diagnosed with dengue.

**^c^** Positive patients were those who had two positive serological tests and negative patients were those with two negative serological tests.

**^d^** Twenty-nine were negative without risk factor and 112 negative with risk factor

1. Distribution of severity of disease in those with the target condition

- Lines 241- 248:

In patients from the acute phase, the qPCR test was positive in 95.7 % of the patients and cPCR in 84.5 %. In patients from the indeterminate chronic phase, qPCR was positive in 68.0% of the cases and in 55.4 % by cPCR. In the cardiac chronic phase, qPCR positivity was 59.1% and 58.6% by cPCR. In all the positive cases *T. cruzi* kPCR was positive. In patients that were negative by serology but with risk factors cPCR (2.6%) and qPCR (3.6%) were positive. In febrile and negative patients without risk factors both tests were negative in all samples.

1. Distribution of alternative diagnoses in those without the target condition

Table 1. Note ^b^ Negative patients comprise a group of febrile patients with negative serology for Chagas disease and diagnosed with dengue.

1. Time interval and any clinical interventions between index test and reference standard

- Lines 223-231:

The inclusion of patients was prospective, whereas the sample collection was both retrospective (for the period 2004-2011) and prospective (for the period 2012-2015). This means that for the retrospective component the samples were part of the repository. The repository consists of 144 samples, collected between 2004 and 2011, and corresponds to serum samples stored at (-80 ° C). In these samples, serological tests were repeated it was found that the results were same that they had been reported at the time of collection of samples and molecular tests were performed. The prospective component consists of 564 samples, collected in the period between 2012 and 2015, and maintained in guanidine hydrochloride solution until processing.

1. Cross tabulation of the index test results (or their distribution) by the results of the reference standard

Appendix S3. Sensitivity and specificity calculations

1. Estimates of diagnostic accuracy and their precision (such as 95% confidence intervals)

Tables 2, 3, 4, and 5 and Figure 2.

1. Any adverse events from performing the index test or the reference standard

NA

DISCUSION

1. Study limitations, including sources of potential bias, statistical uncertainty, and generalizability

- Lines 337-344:

The main limitation involved in this study is the fact that there is not a gold standard test for all clinical phases of Chagas disease. Particularly for chronic phase, the best comparators are serological tests but these techniques measure the immune response and not the relative presence of the parasite. This particular situation impacts the evaluation of new diagnostic tests. This is reflected mainly in the kappa index (Tables 2 and 5) that presented very low values in the undetermined and determined chronic phases. Unfortunately, it has not a simple solution and more understanding of the course of the infection is still needed.

- Lines 402-433:

In addition, the operating capabilities of patients in chronic phase were calculated including all negative (Negatives with and without risk factors). It was observed in the group of negative patients with risk factors a positivity of 2.6% (3 patients) by cPCR and 3.6% (4 patients) by qPCR, possibly due to an immunosuppression issue in these patients preventing the detection of antibodies or infection. Three patients are from the department of Casanare, which is an endemic area, and five patients had less than 24 years of age suggesting a recent infection. Also, all patients reported to know the vectors and have lived during his/her childhood in homes with features such as thatched or ‘barheque’, floor or wood and/or tread walls of earth, wood or ‘barheque’. Two of the seven whose ages were 36 and 51 show the presence of symptoms at the cardiac level. In this group of 7 patients, 4 presented the ELISA absorbance values ​​greater than 0.200 and 4 detectable titles in the IFA (1/8 and 1/16). As the operating capabilities calculated including all negative patients, a small percentage of decreased specificity in the two platforms was observed. The positivity of these serologically negative patients that generated the decrease can probably be explained because cases of recent infection or patients with some form of immunosuppression that has generated the absence of detectable antibodies. In fact, in the group of acute patients, 4 patients whose serology was negative showed positive PCR, in these patients the detection was achieved by direct parasitological methods. Regarding the molecular techniques, given that in all PCR runs were included negative controls including reagents controls, a plausible contamination with parasite DNA is discarded. Significantly, the DP and AUROC values ​​showed no obvious changes unlike the values ​​obtained for the VPN and the Kappa index, in which there was a marked increase. However, the changes obtained do not change the interpretation of the usefulness of the test in the clinical setting, but can show that there are few cases where serological tests may have false negatives as noted previously using cPCR by Ramirez et al., 2009 (23). Even though serological tests are considered the best current option for the diagnosis of Chagas disease, in a meta-analysis of high quality tests their sensitivity has been estimated at 90% (48). Given this, we believe that an improvement of diagnostic tests for Chagas disease is needed for both serology and PCR techniques. An appropriate use of the comparator as gold standard and the inclusion of different phases of the disease are crucial to understand the utility of different diagnostic tests.

1. Implications for practice, including the intended use and clinical role of the index test

- Lines 347-401:

The results obtained for the molecular diagnosis in acute phase were optimal in terms of sensitivity for both qPCR (95.7%; 95%CI: 88.3-98.5) and cPCR (sensitivity 84.5%; 95%CI: 74.3-91.2), and same specificity. Although the results are showing a potential superior performance of the sensitivity of qPCR compared with cPCR, this difference needs a cautious interpretation. Even though there are plausible explanations for this discrepancy, such as the use of hydrolysis probes, the confidence intervals were slightly overlapped, meaning that there is some indication of this difference but it is not statistically significant, so not definitive (25,26). The discriminative power of the molecular tests was high in this phase, mainly for qPCR test (Figure 2; Table 2). This is explained because in the acute phase there is large number of parasites, for example in cases of reactivation in immunosuppressed patients and in oral outbreaks. The values obtained for LR evinced the high probability that positive results correspond to diseased patients (LR+) and the low probability that the diseased patients present negative results (LR-). In addition, the DP was very optimal specifically for qPCR test confirming that this molecular test is very useful for the diagnosis in the acute phase, considering that the direct diagnosis is complex when the parasitemia is low and are required many tests for the confirmation of the acute cases (direct tests, serology tests and clinical information). Regarding the predictive power of molecular tests in the acute phase, these tests are very good predictors of the disease presence when positive results are obtained (PPV) but their performance as predictors of absence of the disease are less (NPV). However, it is worth noting that the predictive values depend on disease prevalence in the evaluated population.

The analysis of operational capabilities in the chronic phase was conducted in the first instance including only negative patients without risk factors or true negatives. For the chronic phase, qPCR sensitivity was 64.2% and 56.8% for cPCR and in concordance with previous reports obtained by qPCR that have shown sensitivity ranging from 60-80% and 20-70% for cPCR (22–24,26,28,41). These sensitivity results may be due to low and intermittent parasitic loads during chronic phase. The performance of qPCR was better than cPCR in the chronic undetermined phase, while that was very similar between the two tests in the determined chronic phase (Table 3 and 5). The discriminative power of the two molecular tests was acceptable in the chronic phase. For qPCR, the AUC and DP values obtained (Table 3 and 5) were better for the undetermined phase than for determined phase. cPCR equally to the AUC value was better in the undetermined phase while the value of DP was greater for the given phase to the determined. The differences between undetermined and determined stages of the chronic phase can be explained by the natural course of the disease, in which the parasitic load decreases while increases the infection time. A decrease in parasitemia probably corresponds to a gradual process between undetermined and heart chronic phase and tis consistent with the natural history of the disease. This is supported by several studies showing that there is no relationship between the evolution of the cardiac form of the disease and parasitemia but it declines with time as observed in this study (42,43). Also, some studies show that cardiac form is mainly related to different types of strains, increased parasitemia, reinfection or immune system disorders in chronic patients (44,45). In the two stages of the chronic phase, there is a high probability that patients with negative results in the molecular tests have the disease (LR-) and these tests are not good predictors of the absence of the disease (NPV) (Table 5). Therefore, the use of molecular methods as diagnostic tests is not appropriate due to the better performance displayed by serology. The probability that the results are positive is high in diseased individuals with respect to healthy individuals (LR +) and the molecular tests are excellent predictors of the presence of disease (PPV). Thus, these tests could be used in situations in which the diagnosis is doubtful, allowing the confirmation of the parasite in diseased patients, which is of great importance for example when monitoring etiological treatment. However, it is necessary to improve the sensitivity, which can be performed by analysing serial samples for each patient as seen in some studies in which such sensitivity improved from 69.2% to 85.2% with the addition of a second sample or conducting DNA extraction from a larger volume of the sample (46,47).

- Lines 434-447:

To our knowledge, this is the first study to include statistical calculation of the sample, which allowed the analysis of operating characteristics of the molecular tests in all clinical phases of Chagas disease. In addition, this study is the first in analysing the two PCR platforms (qPCR and PCR) for the same target (stDNA) in patients from all clinical phases of Chagas disease. The conventional technique was included, due to the vast use of this technique in the diagnosis and its ease implementation in laboratories with restricted equipment (a Real Time PCR machine is not available) (23,24,28). Lastly, acute patients had a less median age than chronic phase patients and in turn the largest number of acute cases are male. This possibly is because economic activity in endemic areas is developed by males that assist to the field and this facilitates direct patient contact with the vector and therefore with the parasite. On the other hand, females ratio and the median age were higher in chronic phase patients that are usually detected by screening blood banks or present cardiac abnormalities in chronic phase, then the detection occurs at a greater age. Additionally, in Colombia most blood donors are women facilitating their diagnosis.

OTHER INFORMATION

1. Registration number and name of registry

- Lines 119-120

The Technical Research Committee and Ethics Research Board at the National Health Institute in Bogotá, Colombia approved the study protocol CTIN-014-11.

1. Where the full study protocol can be accessed

- Lines 119-120

The Technical Research Committee and Ethics Research Board at the National Health Institute in Bogotá, Colombia approved the study protocol CTIN-014-11.

1. Sources of funding and other support; role of funders

- Lines 549-553:

This work was supported by Departamento Administrativo Nacional de Ciencia y Tecnología de Colombia ‘‘Francisco José de Caldas – COLCIENCIAS’’ and ‘‘Unión Temporal Programa Nacional de Investigación para la prevención, control y tratamiento integral de la enfermedad de Chagas en Colombia’’, Grant Number 380- 2011, code 5014-537-30398.
