# Supplementary material for: Molecular Diagnosis of Chagas Disease in Colombia: Parasitic Loads and Discrete Typing Units in Patients from Acute and Chronic Phases
Source: PLoS Negl Trop Dis. 2016 Sep 20;10(9):e0004997. doi: 10.1371/journal.pntd.0004997 (PMC5029947; doi:10.1371/journal.pntd.0004997)

RED CHAGAS Colombia	PROTOCOLOS DE INVESTIGACIÓN<br><br>RED CHAGAS COLOMBIA	
Elaborado por:<br>Juan David Ramírez<br>Coordinador CCDM<br>RED CHAGAS COLOMBIA	Revisado por:<br>Zulma Cucunubá<br>Gerente<br>RED CHAGAS COLOMBIA	

TÍTULO DEL PROYECTO

Caracterización de una cohorte de pacientes con enfermedad de Chagas, su tratamiento etiológico, eventos adversos y respuesta terapéutica

INVESTIGADOR PRINCIPAL

Nombre completo:	Zulma Milena Cucunubá Pérez
Cédula	33367567
Institución	UT- Red Chagas Colombia
Teléfono fijo y celular	3208408971
correo electrónico	zcucunuba@ins.gov.co
Dirección	Calle 22 Bis No. 48-40

CO-INVESTIGADORES

Nombre completo:	Astrid Carolina Flórez Sánchez
Cédula	51866117
Institución	Instituto Nacional de Salud
Teléfono fijo y celular	(1) 2304513 – 3108039570
correo electrónico	aflorez@ins.gov.co
Dirección	Cra. 69 D No. 1-60 Torre 3 Apto 13-02

Nombre completo:	Nubia Lucia Roa Buitrago
Cédula	35458010
Institución	Hospital Universitario San Ignacio
Teléfono fijo y celular	3115616407
correo electrónico	nroa@javeriana.edu.co
Dirección	

Nombre completo:	Diana Carolina Hernández Castro
Cédula	1.030.546.278
Institución	UT-Red Chagas Colombia
Teléfono fijo y celular	3167447716
correo electrónico	dcahernandezc@gmail.com
Dirección	Calle 23 B No. 113-32

Nombre completo:	Mario Javier Olivera
Cédula	1.032.380.726
Institución	Instituto Nacional de Salud
Teléfono fijo y celular	3178949657
correo electrónico	Molivera@ins.gov.co
Dirección	Calle 26 No. 50-26

Nombre completo:	Rafael Herazo
Cédula	8853944
Institución	UT-Red Chagas Colombia
Teléfono fijo y celular	3206733903
correo electrónico	rahetamd@hotmail.com
Dirección	Cra. 113 A No.78-74

Nombre completo:	Juan David Ramírez González
Cédula	1011716118
Institución	UT-Red Chagas Colombia
Teléfono fijo y celular	3188270427
correo electrónico	ccdm.red.chagas@gmail.com
Dirección	Cra. 51 No. 134-67

OBJETIVO GENERAL: Caracterizar un grupo de pacientes con enfermedad de Chagas, su tratamiento etiológico, eventos adversos y respuesta terapéutica.

OBJETIVOS ESPECÍFICOS:

1. Caracterizar un grupo de pacientes con enfermedad de Chagas en fase crónica en Colombia, quienes han recibido tratamiento etiológico.

2. Describir la seguridad y tolerabilidad del medicamento (Benzonidazol ó Nifurtimox), determinada por la frecuencia y distribución de los efectos adversos relacionados.

3. Caracterizar la respuesta al tratamiento etiológico medida por desenlaces clínicos (ecocardiograma), serológicos (IgG anti-T.cruzi) y parasitológicos (PCR)

METODOLOGÍA OBJETIVO ESPECÍFICO 1:

Objetivo 1: *Caracterizar un grupo de pacientes con enfermedad de Chagas en fase crónica en Colombia, quienes han recibido tratamiento etiológico.*

Definición operacional de las variables Objetivo 1

Variable	Definición conceptual	Definición operacional	Tipo de variable y escala de medición	Referencia bibliográfica /comentario
**Fecha de nacimiento**	dd/mm/aaaa de nacimiento	dd/mm/aaaa registrado en el documento de identificación de	Cualitativa Nominal	NA

		referencia del paciente.		
**Edad**	Tiempo vivido por una persona desde el nacimiento.	Edad expresada en años cumplidos basándose en la fecha de nacimiento registrada en documento de identificación de referencia para el paciente (Registro civil, tarjeta de identidad o Cedula de ciudadanía).	Cuantitativa discreta Razón	NA
**Sexo**	Variable biológica que diferencia al hombre de la mujer.	Se registra Masculino o Femenino de acuerdo a lo anotado en la HC.	Cualitativa Dicotómica Nominal	NA
**Etnia**	Grupo humano que comparte y reconoce como propias ciertas características culturales específicas, que determinan su identidad (ethos) frente a otros colectivos.	Se registra la pertenencia a alguno de los siguientes grupos étnicos: Indígena, Mestizo, Afrocolombiano, Blanco, Raizal, Gitano, Mulato u otro, basándose a lo registrado en la HC durante la entrevista clínica.	Cualitativa Politómica Nominal	Dane, 2007*
**Fecha de confirmación diagnostica**	dd/mm/aaaa en que se confirma el diagnostico serológico de infección por T. cruzi.	dd/mm/aaaa de recepción del 2do resultado serológico que confirma el diagnostico o 3er resultado en caso de dos discordantes.	Cualitativa Nominal	Se relaciona con la demora para inicio de tto etiológico y con el tiempo pos-tto para análisis prospectivo
**Fecha de inicio de tratamiento etiológico.**	dd/mm/aaaa de inicio de tratamiento etiológico para infección por T.cruzi.	dd/mm/aaaa de inicio de tratamiento etiológico con Benzonidazol o Nifurtimox.	Cualitativa Nominal	
**Procedencia**				
**Municipio/departamento de Nacimiento.**	Entidad fundamental de la División Político Administrativa del Estado. Pertenece a un departamento.	Identificación del municipio/departamento de nacimiento basándose en los registros de la HC o el documento de identificación de referencia para el paciente (Registro civil, tarjeta de identidad o Cedula de ciudadanía).	Cualitativa Politómica Nominal	Dane, 2007*
**Municipio/departamento de Expedición de la Cedula de ciudadanía. (>18años)**	Entidad fundamental de la División Político Administrativa del Estado. Pertenece a un departamento.	Identificación del municipio/departamento de expedición de la cedula de ciudadanía a través de la observación directa de este documento en los pacientes >18años.	Cualitativa Politómica Nomina	Dane, 2007*<br><br>Villar JC, 2009.
**Municipio /departamento de residencia.**	Entidad fundamental de la División Político Administrativa del Estado. Pertenece a un departamento.	Municipio/departamento de residencia al momento del registro médico, información obtenida de la HC.	Cualitativa Politómica Nominal	Dane, 2007*
**Antecedentes**				
**Nivel**	Grado de escolaridad	Ninguno	Cualitativa	Dane, 2007*

**educativo**	más alto al cual ha llegado la persona de acuerdo con los niveles del sistema educativo formal.	Preescolar<br>Primaria (completo-incompleto)<br>Secundaria (completo-incompleto)<br>Técnico. (completo-incompleto)<br>Tecnológico. (completo-incompleto)<br>Profesional. (completo-incompleto)	Politómica<br>Nominal	
**Familiar(es) con Dx Chagas confirmado**	Familiar(es) del paciente con Dx de Chagas confirmado.	Determinar durante la entrevista clínica y la revisión de la HC antecedentes de familiares con diagnostico de Chagas confirmado.<br>Si - No	Cualitativa dicotómica	Rosas et al, 2002<br><br>Cucunubá et al, 2012
**Parentesco de los familiares con Dx de Chagas confirmado.**	Consanguinidad o afinidad existente entre cada uno de los miembros del hogar y el paciente.	Algún familiar del paciente con Enfermedad de Chagas.<br>Padre, madre, hermano(a), hijo(a), Enumerar otros	Cualitativa Politómica Nominal	Rosas et al, 2002<br><br>Cucunubá et al, 2012
**Familiares con dispositivo cardiaco**	Familiar(es) del paciente que usan o usaron dispositivo cardiaco.	Algún familiar con antecedente de uso de dispositivo cardiaco:<br>Marcapaso<br>Desfibrilador<br>Si - No	Cualitativa Dicotómica Nominal	NA
**Muerte súbita en familiares**	Cese repentino e irreversible de todas las funciones biológicas.	Referencia de muerte súbita en algún familiar. ¿ha muerto algún familiar de manera súbita (repentino, inesperado)?<br>Si - No	Cualitativa Dicotómica Nominal	Rosas et al, 2002
**Medicamentos de uso crónico**	Medicamentos de uso crónico por el paciente.	Determinar durante la entrevista clínica o en la HC, uso de medicamentos para enfermedad crónica.<br>No usa<br>Captopril<br>Digoxina<br>ASA<br>Pednisolona<br>Salbutamol	Cualitativa Politómica Nominal	
**Co-morbilidades crónicas**	Enfermedades asociadas en el paciente con Chagas.	Enumerar co-morbilidades referenciadas diferentes a HTA y Falla Cardiaca. Puede ser:<br>Sin co-morbilidades<br>Diabetes<br>Asma<br>Artritis	Cualitativa Politómica Nominal	
**Transfusión / donación sanguínea**	Procedimiento médico en donde una persona recibe o dona sangre.	Referencia en HC de recepción y/o donación de transfusión sanguínea en algún momento de la vida<br>Ninguna<br>Recepción<br>Donación<br>Donación y recepción	Cualitativa Politómica Nominal	
**Año que**	Año en que el paciente	Expresado en aaaa. (ej, 1993)	Cuantitativa	NA

**recibió transfusión**	con Chagas recibió por 1era vez alguna transfusión.		Discreta<br>De razón	
**Año que realizó donación**	Año en que el paciente con Chagas realizo por última vez alguna donación.	Expresado en aaaa. (ej, 1999)	Cuantitativa<br>Discreta<br>De razón	NA
**Atopía**	Tendencia a desarrollar respuestas de Ac IgE a proteínas ambientales. Enfermedades asociadas con Atopía (eczema, asma y rinitis alérgica).	Antecedente de alguna de las siguientes enfermedades en cualquier momento:<br>Dermatitis atópica<br>Rinitis alérgica<br>Asma.<br>Si – No	Cualitativa<br>Dicotómica<br>Nominal	Avila et al, 2008<br><br>Variable enfocada a establecer la relación entre el antecedente y la presencia de eventos adversos al tto etiológico.
**Colelitiasis**	Presencia de cálculos en la vesícula biliar.	Referencia de colelitiasis con o sin tratamiento quirúrgico.<br>Si – No	Cualitativa<br>Dicotómica<br>Nominal	Crema et al, 2011.<br><br>Matsuda et al, 2009
**HTA**	Elevación de la tensión arterial >=140 PAS o >=90 PAD.	Antecedente de HTA registrado en la HC.<br>Si – No	Cualitativa<br>Dicotómica<br>Nominal	Chobanian et al, 2003<br>Ianni et al, 1998
**Presión arterial**	Elevación de la tensión arterial >=140 PAS o >=90 PAD.	Cifras de tensión arterial registradas en la HC.<br>PAS <120 y PAD <80 (Normal)<br>PAS 120-139 o PAD 80-89 (Pre hipertenso)<br>PAS 140-159 o PAD 90-99 (HTAEstadio I)<br>PAS >160 o PAD >100 (HTAEstadio 2)	Cualitativa<br>Ordinal	Chobanian et al, 2003<br><br>Ianni et al, 1998
**Falla cardiaca**	Incapacidad del corazón de bombear sangre en los volúmenes adecuados.	Historia de insuficiencia cardiaca.<br>Si – No	Cualitativa<br>Dicotómica<br>Nominal	Greenberg B & Kahn AM, 2011
**Clasificación falla cardiaca**	Estado de deterioro de la función cardiaca y su repercusión clínica y factores de riesgo.	Determinar Estadios por la HC:<br>A,B, C, D	Cualitativa<br>Ordinal	Hunt et al, 2005<br><br>Gómez et al, 2007<br><br>Mann, 2011
**Dispositivo**	Aparato que controla	Uso de dispositivo cardiaco.	Cualitativa	Rosas et al,

**electrónico cardiaco**	artificialmente el ritmo cardiaco.	Marcapaso<br>Desfibrilador<br>Si – No	Dicotómica<br>Nominal	2002<br><br>Mora et al, 2007
**Conocimiento del vector**	Identificación de alguno de los vectores (Rhodnius, Triatoma, Panstrongylus) relacionados con la transmisión del T.cruzi.	Conoce o ha visto en algún momento de la vida en su residencia o alrededor de esta algún vector de Chagas.	Cualitativa<br>Dicotómica<br>Nominal	Rosas et al, 2002<br><br>Cucunubá et al, 2012
**Tiempo aproximado desde la última exposición al vector (TAUEV)**	Tiempo transcurrido desde la última vez que vio al vector relacionado con la enfermedad de Chagas.	Determinar a través de la entrevista clínica la edad del paciente cuando vio por última vez a alguno de los vectores para Chagas. Con este dato establecer con referencia a la edad actual el TAUEV expresado en años.	Cuantitativa discreta<br>De razón	Alarcón et al, 2010
**Característica de la vivienda**				
**Techo**	Parte superior de una construcción, que lo cubre y cierra.	Características del techo de la vivienda<br>Paja<br>Palma<br>Madera<br>Zinc<br>Teja<br>Fibra prefabricada<br>Otros	Cualitativa<br>Politómica<br>Nominal	Campbell et al, 2007<br><br>Crocco et al, 2005
**Paredes**	Obra de albañilería vertical, que cierra o limita un espacio.	Características de las paredes de la vivienda durante su niñez.<br>Sin paredes<br>Adobe<br>Tapia pisada<br>Tierra<br>Metálica<br>Ladrillo/bloque<br>Madera<br>Otro material	Cualitativa<br>Politómica<br>Nominal	Campbell et al, 2007<br><br>Crocco et al, 2005<br><br>DANE, 2007*
**Piso**	Pavimento natural o artificial de las habitaciones, etc.	Características del piso de la vivienda<br>Barro<br>Madera<br>Baldosa<br>Cemento<br>Otro material	Cualitativa<br>Politómica	Campbell et al, 2007<br><br>Crocco et al, 2005<br><br>DANE, 2007*
**Ocupación**	Trabajo, empleo, oficio.	Abierta:<br>Hogar<br>Fontanero<br>Madre comunitaria<br>Agricultor	Cualitativa<br>Politómica<br>Nominal	Rosas et al, 2002<br><br>DANE, 2005

		Profesor<br>Estudiante<br>Conductor<br>Promotor de salud<br>Comerciante<br>Desconocida		
**Síntomas**				
**Asintomático**	Sin síntomas relacionados con la enfermedad de Chagas	Paciente que en la entrevista clínica o HC no refiere síntomas como Disnea (clase funcional), palpitaciones, Dolor precordial, constipación y disfagia)<br>Si – No	Cualitativa Dicotómica Nominal	Rosas et al, 2002
**Palpitaciones**	Latido del corazón, sensible e incómodo para el enfermo, y más frecuente que el normal.	Referencia de palpitaciones. Durante la entrevista clínica y/o revisión de la HC.<br>Si – No	Cualitativa Dicotómica Nominal	
**Disnea**	Dificultad para respirar.	Referencia de disnea. Durante la entrevista clínica y/o revisión de la HC. Si- No	Cualitativa Dicotómica Nominal	
**Dolor precordial**	Malestar y dolor sentido en el centro del tórax, generalmente de tipo opresivo que puede tener irradiación a cuello, cabeza y brazos.	Manifestación de dolor en zona central del tórax.<br>Si-No	Cualitativa Dicotómica Nominal	
**Clase funcional NYHA clínica**	Relación entre la limitación de actividad física y la presencia de síntomas cardiacos.	Relación entre la limitación a la actividad física y palpitaciones, disnea, dolor precordial o fatiga. Clasificar clase funcional (CF).<br>CF I<br>CF II<br>CF III<br>CF IV	Cualitativa Ordinal	Greenberg B & Kahn AM, 2011
**Pre-sincope**	Alteración transitoria del nivel de conciencia, sin pérdida completa de ésta.	Sensación de que van a perder la conciencia de forma inminente pero sin llegar a hacerlo.<br>Si-No	Cualitativa Dicotómica Nominal	Rosas et al, 2002<br><br>Peinado et al, 2004
**Sincope**	Pérdida transitoria de conciencia, con pérdida de tono postural, recuperación espontánea, sin maniobras de reanimación.	Historia de algún episodio de Sincope.<br>Si – No	Cualitativa Dicotómica Nominal	Rosas et al, 2002<br><br>Peinado, 2004<br><br>Prata, 2000
**Disfagia**	Dificultad para la deglución.	Historia de disfagia<br>Si – No	Cualitativa Dicotómica	Matsuda et al, 2009

			Nominal	
**Tiempo con presencia de Disfagia.**	Tiempo durante el cual el paciente viene presentando dificultad para la deglución.	Referencia de disfagia expresada en años.	Cuantitativa<br>De razón	Pérez et al, 2011<br><br>Rassi A & Rezende JM, 2011
**Constipación**	Retención de materia fecal o dificultad para eliminarla.	Historia de constipación o estreñimiento.<br>Si – No	Cualitativa<br>Dicotómica<br>Nominal	
**Tiempo con presencia de Constipación.**	Tiempo durante el cual el paciente viene presentando constipación.	Referencia de constipación expresada en años.	Cuantitativa<br>De razón	
**Examen físico**				
**Peso**	Medida derivada de la fuerza gravitacional sobre el cuerpo humano.	Medición del peso corporal al momento del registro médico. Expresado en kilogramos	Cuantitativa<br>Continua<br>De razón	Variables para establecer cambios del peso como efecto adverso e influencia de IMC en PCR
**Talla**	Estatura o altura de las personas.	Medición de la talla al momento del registro médico. Expresado en centímetros.	Cuantitativa<br>Continua<br>Razón	
**Soplo sistólico**	Ruido anormal por el flujo sanguíneo desigual a través de las válvulas cardiacas.	Presencia a la auscultación de soplo cardiaco en sístole.<br>Si – No	Cualitativa<br>Dicotómica<br>Nominal	Rosas et al, 2002
**Radiografía de Tórax**				
**Índice Cardiotorácico (ICT)**	Relación que existe entre el diámetro transverso del corazón y el diámetro transverso del tórax, medidos en la proyección PA de la Radiografía de Tórax.	ICT >0.5<br>Si – No	Cualitativa<br>Dicotómica<br>Nominal	Rassi A Jr. et al, 2006
**Electrocardiograma**				
**Registro ECG Normal**	Ausencia de alteraciones en el registro electrocardiográfico	Lectura del ecg normal en el registro medico.<br>Si – No	Cualitativa<br>Dicotómica<br>Nominal	
**Frecuencia cardiaca**	Numero de despolarizaciones ventriculares en un minuto.	Frecuencia cardiaca en ritmo sinusal expresada en #latidos/minutos.	Cuantitativa<br>Discreta<br>De razón	
**BCRD (bloqueo completo de rama derecha)**	QRS ≥ 120 mseg<br>rsr ', RSR', o 'rSR, en V1 y V2<br>S en DI y V6 ≥40 ms<br>R > 50 ms en V1	Registro de ecg con morfología de BCRD. Si – No	Cualitativa<br>Dicotómica<br>Nominal	
**Bloqueo fascicular anterior**	Eje QRS = -45 a -90 grados, en plano frontal.	Lectura de ecg con BFAI<br>Si – No	Cualitativa<br>Dicotómica<br>Nominal	Rosas et al, 2002

**izquierdo (BFAI)**	qR en aVL<br>QRS <120 ms<br>R en aVL ≥ 45 mseg			Salles et al, 2003<br><br>Olgin J & Zipes DP, 2011<br><br>Mirvis DM & Goldberger AL, 2011<br><br>Rassi A Jr. et al, 2006<br><br>Isselbacher et al, 2010<br><br>Garzón et al, 1995
**Trastornos inespecífico de repolarización**	Depresión leve ST o inversión de la onda T o T aplanada sin causa evidente.	Lectura de ecg con Trastorno inespecíficos de la repolarización.<br>Si – No	Cualitativa<br>Dicotómica<br>Nominal	
**QTd-ECG**	QT(máx.)-QT(min)	< 65ms<br>Si – No	Cualitativa<br>Dicotómica<br>Nominal	
**Bloqueo Bifascicular**	Bloqueo de 2 fascículos<br>1. BCRD + LAFB (patrón BCRD + eje >-45 grados.)<br>2. BCRD + LPFB (patrón BCRD + eje 120 grados.)<br>3. BRI<br>QRS ≥ 120 mseg<br>R muescas en I, aVL, V5 y V6<br>r pequeña/ausente + S profunda en V1 y V2.<br>ausencia de ondas Q septales I, V5 y V6<br>R (> 60 ms) en V5 y V6	ECG con bloqueo bifascicular<br>Grupo 1<br>Grupo 2<br>Grupo 3	Cualitativa<br>Politómica<br>Nominal	
**Bloqueos AV**	Bloqueo variable de la conducción auriculo-ventricular	Grado 1<br>Grado 2<br>Grado 3	Cualitativa<br>Politómica<br>Nominal	
**Extrasístoles ventriculares**	Despolarización ectópica ventricular con uno o múltiples focos.	Pueden ser<br>- Sin extrasístoles<br>- Monomórfica<br>- Polimórfica	Cualitativa<br>Politómica<br>Nominal	
**Bajo voltaje QRS**	Amplitud total del QRS en cada una de las derivaciones: DI-DIII y AvR-AvL-AvF es =<0,5 mV o de V1-V6 =<1,0 mV.	Bajo voltaje en ECG.<br>Si – No	Cualitativa<br>Dicotómica<br>Nominal	
**Fibrilación Auricular**	Arritmia supraventricular con oscilaciones de baja amplitud de línea de base (fibrilación o las ondas f) y un ritmo ventricular irregular.	Ecg con FA<br>Si – No	Cualitativa<br>Dicotómica<br>Nominal	
**Zonas de inactividad**	Zonas que no produce fuerza eléctricas (no	Lectura de ECG con ZIE	Cualitativa<br>Dicotómica	

**eléctrica (ZIE)**	despolariza ni repolariza), se expresa como Onda Q.		Nominal	
**Holter**				
**Taquicardia ventricular no sostenida**	Presencia de 3 o + latidos consecutivos originados en tejido ventricular con una frecuencia promedio de 100 latidos por minuto durante no más de 30 segundos.	Presencia de TVNS en Holter<br>Si - No	Cualitativa<br>Dicotómica<br>Nominal	Rassi A Jr. et al, 2006<br><br>Grupi et al, 1995
**Registro Holter Normal**	Ausencia de alteraciones en el registro Holter	Lectura del Holter normal en el registro medico.<br>Si – No	Cualitativa<br>Dicotómica<br>Nominal	Rosas et al, 2002<br><br>Salles et al, 2003<br><br>Olgin J & Zipes DP, 2011<br><br>Mirvis DM & Goldberger AL, 2011<br><br>Rassi A Jr. et al, 2006<br><br>Isselbacher et al, 2010<br><br>Garzón et al, 1995
**BCRD (bloqueo completo de rama derecha)**	QRS ≥ 120 mseg<br>rsr ', RSR', o 'rSR, en V1 y V2<br>S en DI y V6 ≥40 ms<br>R > 50 ms en V1	Registro del Holter con morfología de BCRD.<br>Si – No	Cualitativa<br>Dicotómica<br>Nominal	
**Bloqueo fascicular anterior izquierdo (BFAI)**	Eje QRS = -45 a -90 grados, en plano frontal.<br>qR en aVL<br>QRS <120 ms<br>R en aVL ≥ 45 mseg	Lectura del Holter con BFAI<br>Si – No	Cualitativa<br>Dicotómica<br>Nominal	
**Trastornos inespecífico de repolarización**	Depresión leve ST o inversión de la onda T o T aplanamiento de la onda sin causa evidente.	Lectura de ecg con Trastorno inespecíficos de la repolarización.<br>Si – No	Cualitativa<br>Dicotómica<br>Nominal	
**QTd - Holter**	QT (máx.)-QT(min)	< 65ms<br>Si – No	Cualitativa<br>Dicotómica<br>Nominal	
**Bloqueo Bisfacicular**	Bloqueo de 2 fascículos<br>1. BCRD + LAFB (patrón BCRD + eje >-45 grados.)<br>2. BCRD + LPFB (patrón BCRD + eje 120 grados.)<br>3. BRI<br>QRS ≥ 120 mseg<br>R muescas en I, aVL, V5 y V6<br>r pequeña/ausente + S profunda en V1 y V2.<br>ausencia de ondas Q septales I, V5 y V6<br>R (> 60 ms) en V5 y	ECG con bloqueo bifascicular<br>Grupo 1<br>Grupo 2<br>Grupo 3	Cualitativa<br>Politómica<br>Nominal	

	V6			
**Bloqueos AV**	Bloqueo variable de la conducción auriculo-ventricular	Grado 1<br>Grado 2<br>Grado 3	Cualitativa<br>Politómica<br>Nominal	
**Extrasístoles ventriculares monomórfica**	Despolarización de un foco ectópico ventricular.	Numero de extrasístoles monomórficas registradas durante el registro Holter.	Cuantitativa<br>De razón	
**Extrasístoles ventriculares polimórficas (multifocales)**	Despolarización ectópica ventricular con uno o múltiples focos.	Numero de extrasístoles registradas durante el registro Holter	Cuantitativa<br>De razón	
**Fibrilación Auricular**	Arritmia supraventricular con oscilaciones de baja amplitud de línea de base (fibrilación o las ondas f) y un ritmo ventricular irregular.	Holter con FA sostenida o FA paroxísticas<br>Si - No	Cualitativa<br>Dicotómica<br>Nominal	
**Ecocardiograma (ECG)**				
**Aneurisma apical de VI (ventrículo izquierdo)**	Dilatación localizada en pared de VI.	Visualización en eco de aneurisma apical VI.<br>Si – No	Cualitativa<br>Dicotómica<br>Nominal	
**Trombo intracavitario**	Coagulo de sangre en VI	Visualización en eco de trombo intracavitario.<br>Si – No	Cualitativa<br>Dicotómica<br>Nominal	
**Incremento Diámetro VI al final de la diástole**	Aumento diámetro VI al final de la diástole.	Si - No	Cualitativa<br>Dicotómica<br>Nominal	Rassi A Jr. et al,<br>2006
**Incremento diámetro VI sístole**	Aumento diámetro VI durante la contracción.	Si - No	Cualitativa<br>Dicotómica<br>Nominal	Rassi A Jr. et al, 2007
**Fracción de eyección VI**	Proporción de sangre expulsada en cada contracción ventricular.	Expresado en %	Cuantitativa<br>Continua<br>De razón	Viotti et al, 2004<br><br>Isselbacher et al, 2010
**Anormalidad segmentaria o global de la movilidad de VI**	Alteración en la movilidad de la pared ventricular.	Si - No	Cualitativa<br>Dicotómica<br>Nominal	Chaves et al, 2006
**Aumento velocidad onda A mitral**	Incremento de la velocidad de la onda A mitral. Con consiguiente disminución del índice E/A.	Si – No	Cualitativa<br>Dicotómica<br>Nominal	Acquatela et al, 2007
**Disminución del índice E/A**		Si – No	Cualitativa<br>Dicotómica<br>Nominal	
**Aumento del tiempo relajación Isovolumétrica**	Prolongación del tiempo de relajación del VI.	Si - No	Cualitativa<br>Dicotómica<br>Nominal	

**VI**				
**Alteración valvular mitral**	Incompetencia valvular mitral durante su cierre o apertura.	Si - No	Cualitativa Dicotómica Nominal	
**Clasificación Miocardiopatía**				
**Clasificación AHA**	Clasificaciones que establecen la relación entre la presencia o ausencia de síntomas cardiacos y los hallazgos de normalidad o anormalidad en el Electrocardiograma, Ecocardiograma o la radiografía de tórax.	Clasificación según AHA de acuerdo a lo registrado en la HC.<br>A, B, C, D	Cualitativa Ordinal	Hunt et al, 2005<br><br>Gómez et al, 2007<br><br>Andrade et al, 2011<br><br>Ministerio da Saude, 2005
**Clasificación Consenso. Brasil.**		Clasificación según Cons. Brasil de acuerdo a lo registrado en la HC.<br>A, B1, B2, C, D.	Cualitativa Ordinal	
**Clasificación Andes Modificada.**		Clasificación según Andes modificada de acuerdo a lo registrado en la HC.<br>IA, IB, II, III.	Cualitativa Ordinal	
**Clasificación Kuschnir**		Clasificación según Kuschnir modificada de acuerdo a lo registrado en la HC.<br>0, I, II, III	Cualitativa Ordinal	
**Laboratorio**				
**IFI positividad**	Presencia de anticuerpos IgG anti-*T. cruzi* en suero, detectados mediante fluorescencia	Reactivo/No reactivo	Cualitativa Dicotómica Nominal	Storch W ,2000
**IFI títulos**	Contenido de anticuerpos IgG anti-*T.cruzi* en diluciones seriadas del suero que se unen a una cantidad estándar de antígeno y determinan el nivel en el cual se produce la reacción positiva, detectada por fluorescencia.	1/8, 1/16, 1/32, 1/64,1/128, 1/256, 1/512,1/1024, 1/2048	Cualitativa Ordinal	Roitt, 2008
**ELISA Positividad**	Presencia de anticuerpos IgG anti-*T. cruzi* en suero unidos al antígeno, detectados mediante espectrofotometría.	Positivo/Negativo	Cualitativa Dicotómica Nominal	Crowther, 1995<br><br>Venkatesan, 1993
**ELISA Absorbancia**	Medida espectrofotométrica de anticuerpos IgG-anti *T.cruzi* en función de su afinidad y concentración.	0.0-2.5	Cuantitativa Intervalo	Venkatesan P and Wakelin D, 1993
**HAI Positividad**	Presencia de anticuerpos IgG anti-*T. cruzi* en suero,	Reactivo/No reactivo	Cualitativa Dicotómica Nominal	Gilbert, 1992

	detectados mediante aglutinación.			
**TESAblot**	Presencia de anticuerpos IgG anti-*T. cruzi* en suero, detectados por Westernblot utilizando TESA (antígenos de secreción y excreción obtenidos mediante cultivo de formas tripomastigotes de *T. cruzi*)	Positivo/ Negativo	Cualitativa Dicotómica Nominal	Umezawa, 1996 Ramírez et al., 2009 Nakazawa et. al.,2001
**PCR Positividad**	Detección de ADN de *T.cruzi* en sangre mediante amplificación enzimática de ADN diana.	Positivo/Negativo	Cualitativa Dicotómica Nominal	Higuchi R, 1993 Basquiera et al., en 2003 Hidron et al, 2010 Ramírez et al., 2009
**PCR Carga parasitaria**	Medición de la concentración inicial delADN diana en muestras de sangre, equivalente al número de parásitos por mL.	$0.0 - 1 \times 10^5$	Cuantitativa Razón	Higuchi R, 1993 Moreira et al., 2013
**Genotipificación de *T.cruzi***	Determinación del contenido genómico para clasificación de *T.cruzi* en Unidades de Tipificación Discretas en ADN de *T.cruzi*.	DTU´s:<br>TcI<br>TcII<br>TcIII<br>TcIV<br>TcV<br>TcVI	Cualitativa Politómica Nominal	Zingales et al., 2010 y 2012 Ramírez et al., 2010

Tipo de estudio y diseño general Objetivo 1

Estudio observacional de cohorte retrospectiva. Para este objetivo, se analizarán las variables de línea de base, definida como el momento de inicio del tratamiento.

Universo de estudio, selección y tamaño de muestra, unidad de análisis y observación.

El tamaño de la muestra que será utilizado en este estudio estará conformado aproximadamente por pacientes 785 pacientes quienes han recibido terapia tripanocida en el Instituto Nacional de Salud, Instituto Colombiano de Medicina

Tropical, Secretaría de Salud de Boyacá y Secretaría de Salud de Casanare. De acuerdo a las bases de datos, cada una de estas instituciones aglomera un número aproximado de 400, 20, 120 y 125 pacientes respectivamente.

Criterios de inclusión y exclusión Objetivo 1

Se incluirán los pacientes que iniciaron el manejo etiológico por enfermedad de Chagas entre los años 2000 y 2012 en las siguientes instituciones; Instituto Nacional de Salud, Instituto Colombiano de Medicina Tropical, Secretaría de Salud de Boyacá y Secretaría de Salud de Casanare.
Se excluirán los pacientes con diagnóstico de enfermedad de Chagas fase aguda y los menores de 18 años de edad.

Procedimientos para la recolección de información, instrumentos a utilizar y métodos para el control y calidad de los datos Objetivo 1

En coordinación con el Centro Coordinador de Datos y Muestras (CCDM) se diseñara el formato de historia clínica sistematizada, para su validación se realizarán pruebas piloto con algunos pacientes, buscando ajustar términos y variables. Una vez validada, la historia clínica sistematizada será el documento de referencia para el manejo de la información de cada paciente.
Con respecto al proceso de recolección de la información, esta será digitada utilizando la historia clínica sistematizada, de esta, se extraerán los datos necesarios para la descripción y análisis de las variables. Se realizarán entrenamientos sucesivos a los investigadores en la aplicación de los formatos de recolección de la información y se realizarán pilotos para el ajuste y resolución de dudas específicas en las variables del documento. Se harán dos evaluaciones de la calidad de la información registrada en los formatos, por medio de verificación de una muestra aleatoria del total de los formatos registrados, correspondiente al 5% de las mismas. Errores superiores al 20% del total de variables registradas obligarán a la revisión del total de la información.

Dificultades y limitaciones del estudio Objetivo 1

Dada la naturaleza retrospectiva de esta fase del análisis, la principal limitación de esta fase del estudio se relaciona con valores perdidos. El manejo de estas pérdidas de información se explica durante el plan de análisis.

Plan de análisis de los resultados Objetivo 1

Una vez exportada la base y evaluada la calidad de los datos, se realizará un análisis exploratorio de la información. Se obtendrán histogramas y box-plot para las variables continuas y gráficos de barras para variables categóricas. Se evaluarán las pérdidas de información

para cada variable, según si son informativas o sugieren sesgo. Pérdidas de información superiores a 20% en la variable implicará su exclusión del análisis y evaluación de sesgo de información. En pérdidas inferiores al 20% se evaluará la probabilidad de sesgo de información y en caso de ameritarlo serán tratadas con estrategias de imputación y análisis de sensibilidad para las mismas. Para cada medida de asociación se estimarán los intervalos de confianza al 95%. Se empleará el software Stata 10.0 (StataCorp. 2007. *Stata Statistical Software: Release 10.* College Station, TX: StataCorp LP.)

Métodos y modelos de análisis de los datos según tipo de variables Objetivo 1

Las variables continuas serán descritas como medias con desviación estándar, mediana y rango intercuartílico y las variables categóricas como porcentajes.

METODOLOGÍA OBJETIVO ESPECÍFICO 2:

Objetivo 2: *Describir la seguridad y tolerabilidad del medicamento (Benzonidazol ó Nifurtimox), determinada por la frecuencia y distribución de los efectos adversos relacionados.*

Definición operacional de las variables objetivo 2

La clasificación por sistemas se hizo de acuerdo al sistema de terminología de efectos adversos de la organización mundial de la salud WHO-ART CLASS (Terminology for coding clinical information in relation to drug therapy) (WHO-ART, 2005).

Variable	Definición conceptual	Definición operacional	Tipo de variable y escala de medición	Referencia
**Tratamiento etiológico**	Medicamento que se emplea para tratar la enfermedad de Chagas	Clasificar en Benzonidazol o en Nifurtimox	Cualitativa nominal dicotómica	MPS, 2010
**Dosis**	Cantidad de Benzonidazol o Nifurtimox suministrado en mg/Kg/día.	Dosis de Nifurtimox o Benzonidazol	Cuantitativa continua de razón	MPS, 2011
**Número de tabletas tomadas**	Cantidad de tabletas de Nifurtimox o Benzonidazol ingeridas al día.	Conteo de tabletas ingeridas.	Cuantitativa discreta de razón	MPS, 2011
**Duración del Tratamiento**	Tiempo que el paciente ingiere Nifurtimox o Benzonidazol	Días que el paciente tomó Nifurtimox o Benzonidazol	Cuantitativa discreta de razón	MPS, 2012

**Suspensión temporal del tratamiento**	Interrupción por un tiempo definido del uso de Benzonidazol o Nifurtimox	Clasificar en Suspensión temporal del tratamiento o no.	Cualitativa nominal dicotómica	Coelho et al., 1999
**Días de suspensión temporal del tratamiento**	Número de días que el paciente no ingirió Nifurtimox o Benzonidazol debido a una suspensión temporal del tratamiento.	Conteo de días	Cuantitativa discreta de razón	Coelho et al., 1999
**Suspensión definitiva del tratamiento**	Interrupción definitiva del uso de Benzonidazol o Nifurtimox	Clasificar en Suspensión definitiva del tratamiento o no.	Cualitativa nominal dicotómica	Coelho et al., 1999
**Número de Hospitalizaciones**	Cantidad de veces que tuvo que ser atendido en un hospital durante el tratamiento etiológico Nifurtimox o Benzonidazol por una reacción adversa.	Conteo de hospitalizaciones	Cuantitativa discreta de razón	Coelho et al., 1999
**Trastornos de piel y anexos**				
**Eritema**	Inflamación superficial de la piel, caracterizada por manchas rojas.	Clasificar en presencia o en ausencia de eritema	Cualitativa nominal dicotómica	Pinazo et al., 2010
**Eritema multiforme**	Tipo de reacción de hipersensibilidad con Inflamación superficial de la piel, generalizada que se caracterizada por muchas manchas rojas que confluyen.	Clasificar en presencia o en ausencia de eritema multiforme	Cualitativa nominal dicotómica	Pinazo et al., 2010
**Eczema**	Erupción cutánea con eritema, edema, vesículas, costras, escamas.	Clasificar en presencia o en ausencia de eczema	Cualitativa nominal dicotómica	Altcheh et al., 2011
**Prurito**	Picazón o irritación en la piel que despierta la necesidad de rascarse esa área	Clasificar en presencia o en ausencia de prurito	Cualitativa nominal dicotómica	Pinazo et al., 2010
**Síndrome Stevens Johnson.**	.Es una variante severa del eritema multiforme, consistente en una reacción de hipersensibilización que afecta la piel y las membranas mucosa	Clasificar en presencia o en ausencia de Stevens Johnson.	Cualitativa nominal dicotómica	Yun et al., 2009

**Urticaria**	Erupción de la piel caracterizada por lesiones cutáneas edematosas, de contornos delimitados y con un halo eritematoso	Clasificar en presencia o en ausencia de urticaria	Cualitativa nominal dicotómica	Pinazo et al., 2010
**Trastornos del sistema músculo-esquelético.**				
**Artralgia**	Dolor de las articulaciones.	Clasificar en presencia o en ausencia de artralgia	Cualitativa nominal dicotómica	Pinazo et al., 2010
**Calambres**	contracciones o espasmos súbitos, involuntarios en uno o más músculos	Clasificar en presencia o en ausencia de artralgia	Cualitativa nominal dicotómica	Altcheh et al., 2011
**Mialgia**	Dolor muscular	Clasificar en presencia o en ausencia de mialgia	Cualitativa nominal dicotómica	Pinazo et al., 2010
**Trastornos del sistema nervioso central y periférico**				
**Mareos**	Sensación de que se podría desmayar.	Clasificar en presencia o en ausencia de mareos	Cualitativa nominal dicotómica	Altcheh et al., 2011
**Pérdida temporal de la memoria**	Olvido inusual que dificulta recordar hechos nuevos.	Clasificar en presencia o en ausencia de pérdida de la memoria	Cualitativa nominal dicotómica	Valencia et al., 2012
**Neuropatía periférica**	Daño del sistema nervioso periférico. Aparece como una sensación de hormigueo, quemazón, endurecimiento o adormecimiento de los pies, incluyendo los dedos. También puede producir una sensación de cosquilleo, dolor sin motivo o sensaciones que parecen ser más intensas que las normales.	Clasificar en presencia o en ausencia de Neuropatía periférica	Cualitativa nominal dicotómica	Coura et al.,1997
**Somnolencia**	Sentirse anormalmente soñoliento durante el día.	Clasificar en presencia o en ausencia de somnolencia	Cualitativa nominal dicotómica	Coura et al.,1997

**Dolor de cabeza**	Cefalalgia intermitente, que afecta ordinariamente a uno de los lados de la cabeza	Clasificar en presencia o en ausencia de dolor de cabeza	Cualitativa nominal dicotómica	Pinazo et al., 2010
**Parestesia**	Sensación o conjunto de sensaciones anormales como el hormigueo, adormecimiento o ardor	Clasificar en presencia o en ausencia de parestesia	Cualitativa nominal dicotómica	Pinazo et al., 2010
**Trastornos de la visión**				
**Visión borrosa**	Pérdida de la agudeza visual y la incapacidad para visualizar pequeños detalles	Clasificar en presencia o en ausencia de visión borrosa	Cualitativa nominal dicotómica	Altcheh et al., 2011
**Audición y los trastornos vestibulares**				
**Vértigo**	Sensación de movimiento o giros. Los pacientes sienten como si realmente estuvieran girando o moviéndose, o como si el mundo estuviera girando a su alrededor.	Clasificar en presencia o en ausencia de vértigo	Cualitativa nominal dicotómica	Carrilero et al., 2011
**Tinitus**	Acúfenos o zumbidos del oído.	Clasificar en presencia o en ausencia de tinitus.	Cualitativa nominal dicotómica	Valencia et al., 2012
**Trastornos psiquiátricos**				
**Insomnio**	Trastorno del sueño en el cual los pacientes tienen dificultades para quedarse dormidas, para continuar durmiendo o para hacer ambas cosas	Clasificar en presencia o en ausencia de insomnio	Cualitativa nominal dicotómica	Carrilero et al., 2011
**Irritabilidad**	Estado de ánimo o rasgo de personalidad caracterizado por reacciones afectivas exageradas, de ira o de malhumor	Clasificar en presencia o en ausencia de irritabilidad	Cualitativa nominal dicotómica	Guhl et al., 2001
**Trastornos sistema gastrointestinal**				
**Distensión abdominal**	Es una afección en la que el abdomen (vientre) se siente lleno y apretado.	Clasificar en presencia o en ausencia de distensión abdominal	Cualitativa nominal dicotómica	Pinazo et al., 2010
**Diarrea**	Síntoma o fenómeno morboso que consiste en evacuaciones de vientre líquidas y frecuentes.	Clasificar en presencia o en ausencia de diarrea	Cualitativa nominal dicotómica	Pinazo et al., 2010

**Náusea**	Sensación de malestar o de estómago revuelto junto con una urgencia por vomitar	Clasificar en presencia o en ausencia de náusea	Cualitativa nominal dicotómica	Pinazo et al., 2010
**Epigastralgia**	Sensación de dolor en la boca del estómago.	Clasificar en presencia o en ausencia de epigastralgia	Cualitativa nominal dicotómica	Pinazo et al., 2010
**Dolor abdominal**	Sensación desagradable en la región comprendida entre el pecho hasta la ingle	Clasificar en presencia o en ausencia de dolor abdominal	Cualitativa nominal dicotómica	Altcheh et al., 2011
**Boca seca**	Sensación de que no hay suficiente saliva en su boca	Clasificar en presencia o en ausencia de boca seca	Cualitativa nominal dicotómica	de Pontes et al., 2010
**Pirosis**	Sensación de ardor dolorosa en el pecho o la garganta	Clasificar en presencia o en ausencia de pirosis	Cualitativa nominal dicotómica	Pinazo et al., 2010
**Vómito**	Expulsión violenta y espasmódica del contenido del estómago a través de la boca	Clasificar en presencia o en ausencia de vómito	Cualitativa nominal dicotómica	Pinazo et al., 2010
**Trastornos metabólicos y nutricionales**				
**Anorexia**	Falta anormal de ganas de comer	Clasificar en presencia o en ausencia de anorexia	Cualitativa nominal dicotómica	Pinazo et al., 2010
**Disminución de Peso**	Pérdida del 10% o más del peso que no se hace de manera voluntaria.	Identificar disminución de por lo menos un 1.67% de peso corporal durante la toma del medicamento comparado con el peso inicial antes de comenzar el tratamiento etiológico	Cualitativa nominal dicotómica	Valencia et al., 2012
**Polifagia**	Aumento anormal de la necesidad de comer	Clasificar en presencia o en ausencia de polifagia	Cualitativa nominal dicotómica	de Pontes et al., 2010
**Trastornos generales del Cuerpo en general**				
**Astenia**	Falta o decaimiento considerable de fuerzas.	Clasificar en presencia o en ausencia de astenia	Cualitativa nominal dicotómica	de Pontes et al., 2010

**Fiebre**	Aumento en la temperatura corporal por encima de lo que se considera normal.	Sensación subjetiva de ausencia o presencia de fiebre.	Cualitativa nominal dicotómica	Pinazo et al., 2010
**Paraclínicos**				
**Leucocitos**	Células que están principalmente en la sangre y circulan por ella con la función de combatir las infecciones o cuerpos extraño	Conteo de leucocitos en el hemograma.	Cuantitativa discreta de razón	Altcheh et al., 2011
**Neutrófilos**	un tipo de glóbulo blanco, de tipo de granulocito, cuya principal función es fagocitar y destruir a bacterias y participar en el inicio del proceso inflamatorio	Conteo absoluto de Neutrófilos en el hemograma	Cuantitativa continua de rezón	Carrilero et al., 2011
**Eosinofilos**	Célula fagocitaria del sistema inmunológico que elimina los complejos antígeno-anticuerpo y que por su capacidad citotóxica tiene una función de defensa ante los microorganismos no fagocitables, como los parásitos	Conteo absoluto de Eosinofilos en el hemograma	Cuantitativa continua de rezón	Altcheh et al., 2011
**Plaquetas**	Células que circulan en la sangre, participan en la formación de coágulos sanguíneos y en la reparación.	Conteo de Plaquetas en el hemograma	Cuantitativa discreta de razón	Carrilero et al., 2011
**Nitrógeno ureíco en la sangre.**	Es un producto que se forma cuando la proteína se descompone.	Nivel de nitrógeno ureíco en sangre	Cuantitativa continua de rezón	Carrilero et al., 2011
**Creatinina**	Es un producto de degradación de la creatina, una parte importante del músculo.	Nivel de creatinina en sangre	Cuantitativa continua de rezón	Carrilero et al., 2011
**Transaminasa Glutamo oxalacética**	Enzima que mide la función citoplasmática y mitocondrial. Del hígado.	Nivel de Transaminasa Glutamo oxalacética en sangre	Cuantitativa continua de rezón	Altcheh et al., 2011
**Transaminasa Glutamo pirúvica.**	Enzima hepática que mide la función citoplasmática.	Nivel de Transaminasa Glutamo pirúvica en sangre	Cuantitativa continua de rezón	Altcheh et al., 2011

Tipo de estudio y diseño general Objetivo 2

Estudio observacional, analítico tipo cohorte retrospectiva. Para este objetivo, se analizará la incidencia de efectos adversos durante el tratamiento etiológico de los pacientes. La información será obtenida de las historias clínicas.

Universo de estudio, selección y tamaño de muestra, unidad de análisis y observación. Criterios de inclusión y exclusión Objetivo 2

El tamaño de la muestra que será utilizado en este estudio estará conformado aproximadamente por pacientes 785 pacientes quienes han recibido terapia tripanocida en el Instituto Nacional de Salud, Instituto Colombiano de Medicina Tropical, Secretaría de Salud de Boyacá y Secretaría de Salud de Casanare. De acuerdo a las bases de datos, cada una de estas instituciones aglomera un número aproximado de 400, 20, 120 y 125 pacientes respectivamente.

Criterios de Inclusión:

- Registros de pacientes adultos que hayan recibido tratamiento tripanocida por diagnóstico de enfermedad de Chagas fase crónica.
- Registros de pacientes que tengan por lo menos un control de seguimiento médico en donde se identificaron síntomas relacionados con efectos adversos durante el tratamiento: días 20, 40 o 60.
- Registros de pacientes que tengan por lo menos en 1 control de seguimiento médico durante el tratamiento: días 20, 40 o 60, con resultados de los siguientes exámenes de laboratorio (cuadro hemático, parcial de orina, pruebas de función hepática y renal).

Criterios de exclusión:

- Registros de pacientes con diagnóstico de enfermedad de Chagas fase aguda.
- Registros de pacientes con edad menor a 18 años.

Procedimientos para la recolección de información, instrumentos a utilizar y métodos para el control y calidad de los datos Objetivo 2

En la sección de tratamiento etiológico de la historia clínica sistematizada se registrarán los efectos adversos a medicamentos tripanocida previamente reconocidas en la literatura científica. Se

realizarán procedimientos de evaluación de la calidad del registro de la información con la revisión del 5% de las variables registradas al 30, 60 y 100% del registro de los datos, seleccionados de manera aleatoria. Variables con errores superiores al 10% o errores superiores al 20% de los datos registrados al momento del análisis obligarán a la revisión de la totalidad de la base. Se realizarán entrenamientos sucesivos a los investigadores en la aplicación de los formatos de recolección de la información y se realizarán pilotos para el ajuste y resolución de dudas específicas en las variables del documento.

Dificultades y limitaciones del estudio Objetivo 2

En esta fase, la historia clínica sistematizada debe tener la información necesaria para garantizar los datos requeridos. Hay riesgo de sesgo de información, en la medida que efectos poco sintomáticos para los pacientes, no estén registrados en las historias clínicas y no se pueda valorar causalidad. Igualmente, puede encontrarse subregistro por interrupción del manejo por parte del paciente debido a los efectos adversos. Además existe riesgo de sesgo por indicación en el cual no es claro como fue el proceso de la asignación para la intervención farmacológica.

Plan de análisis de los resultados Objetivo 2

Una vez exportada la base y evaluada la calidad de los datos, se realizará un análisis exploratorio de la información. Se obtendrán histogramas y box-plot para las variables continuas y gráficos de barras para variables categóricas. Las variables continuas serán descritas como medianas y rango intercuartílico y las variables categóricas como porcentajes. Se evaluarán las pérdidas de información para cada variable, según si son informativas o sugieren sesgo. Pérdidas de información superiores a 20% en la variable implicará su exclusión del análisis y evaluación de sesgo de información. En pérdidas inferiores al 20% se evaluará la probabilidad de sesgo de información y en caso de ameritarlo serán tratadas con estrategias de imputación y análisis de sensibilidad para las mismas.
Se empleará el software Stata 10.0 (StataCorp. 2007. *Stata Statistical Software: Release 10.* College Station, TX: StataCorp LP.)

Métodos y modelos de análisis de los datos según tipo de variables Objetivo 2

El análisis de la incidencia de los efectos adversos se realizará con modelo de sobrevida actuarial libre de efectos adversos, por medio de un gráfico de Kaplan-Meier, que permitirá modelar la ocurrencia de los efectos adversos en el tiempo. Se obtendrá un desenlace combinado de efectos adversos ocurridos, así como la modelación de los diferentes

efectos adversos observados. Posteriormente, para evaluar la ocurrencia de los efectos adversos en relación a variables de interés, se aplicará la prueba de Long Rank y se obtendrán Hazard Ratios por medio del modelo de sobrevida de Cox, para identificar potenciales factores de riesgo relacionados con los efectos adversos
Para evaluar causalidad se empleará la escala de Naranjo (WHO, 2003)
La severidad de los efectos adversos se evaluará según la escala de la Organización Mundial para la salud. (Coelho et al., 1999)

Justificación del presupuesto solicitado para cada objetivo específico:

Para el cumplimiento de este objetivo se contará con la participación de dos Médicos, entre ellos un Joven investigador. Realizarán la detección y descripción de los efectos adversos en los pacientes sometidos a tratamiento.
Con el fin de recolectar información de pacientes se realizarán viajes a los departamentos de Casanare y Boyacá, financiados por Colciencias. Finalmente, se contará presupuesto para llevar a cabo la divulgación y publicación de los resultados de la caracterización en eventos científicos y revistas científicas.

METODOLOGÍA OBJETIVO ESPECÍFICO 3

Definición operacional de las variables Objetivo 3:

Para determinar las variables del presente objetivo, sobre todo aquellas que valoran los cambios en el tiempo del progreso clínico, se tendrá en cuenta que las variables del objetivo 1 que hacen referencia a clase funcional, electrocardiograma, Holter, Ecocardiograma y clasificación de cardiomiopatía chagasica, serán evaluadas y registradas en la historia clínica sistematizada en cada seguimiento anual.

Variable	Definición conceptual	Definición operacional	Tipo de variable y escala de medición	Referencia
**Años de seguimiento**	Tiempo contado a partir del fin del tratamiento.	Tiempo entre el fin de tratamiento hasta la valoración de seguimiento. Expresado en años.	Cuantitativa<br>Continua<br>De razón	NA
**Edad de tratamiento**	Edad cumplida al momento de recibir el tto etiológico	Expresada en años	Cuantitativa<br>Continua<br>De razón	NA
**Tiempo de tratamiento**	Número de días efectivos que tomó medicamento	Expresado en número de días.	Cuantitativa<br>Discreta<br>De razón	NA

**Cambios clínicos**				
**Palpitaciones**	Latido del corazón, sensible e incómodo para el enfermo, y más frecuente que el normal.	Entrevista clínica de seguimiento para definir:<br>- Ausencia<br>- Mejoría (<frecuencia y <intensidad)<br>- Empeoramiento (>frecuencia o >intensidad)	Cualitativa Ordinal	Rosas et al, 2002
**Disnea**	Dificultad para respirar.			
**Dolor precordial**	Malestar y dolor sentido en el centro del tórax, generalmente de tipo opresivo que puede tener irradiación a cuello, cabeza y brazos.			
**Clase funcional al seguimiento**				
**Variación de la clase funcional NYHA**	Relación entre la limitación de actividad física y la presencia de síntomas cardiacos	Valoración clínica de seguimiento anual años, Cambio en la CF así:<br>- Mejoró CF<br>- Igual CF<br>- Empeoró CF	Cualitativa Ordinal	Villar JCI, 2002
**Cambio de clasificación miocardiopatía AHA**	Clasificaciones que establecen la relación entre la presencia o ausencia de síntomas cardiacos y los hallazgos de normalidad o anormalidad en el Electrocardiograma, Ecocardiograma o la radiografía de tórax.	Cambio de grupo según AHA durante el seguimiento.<br>- Mejoró clasificación<br>- Igual clasificación<br>- Empeoró clasificación	Cualitativa Ordinal	Viotti et al, 2004
**Cambio de clasificación miocardiopatía Cons. Brasil.**		Cambio de grupo según Consenso de Brasil durante el seguimiento<br>- Mejoró clasificación<br>- Igual clasificación<br>- Empeoró clasificación	Cualitativa Ordinal	Greenberg B & Kahn AM, 2011
**Cambio de clasificación miocardiopatía Andes Mod.**		Cambio de grupo según Andes modificada durante el seguimiento.<br>- Mejoró clasificación<br>- Igual clasificación<br>- Empeoró clasificación	Cualitativa Ordinal	Lauria et al, 2000
**Cambio de clasificación miocardiopatía Kuschnir**		Cambio de grupo según Kuschnir durante el seguimiento.<br>- Mejoró clasificación<br>- Igual clasificación<br>- Empeoró clasificación	Cualitativa Ordinal	Marin-Neto et al, 2009
**Numero de hospitalizaciones / año**	Cantidad de hospitalizaciones por año.	Promedio de hospitalizaciones de causa cardiaca por año. Determinado en las consultas anuales de seguimiento.	Cuantitativa Continua De razón	Viotti et al,

**Resucitación cardio-cerebro-pulmonar.**	Maniobras de soporte vital avanzado en un paro cardio-respiratorio	Necesidad de resucitación cardio-cerebro-pulmonar de causa no traumática el algún momento del tiempo post-tratamiento.<br>Si – No	Cualitativa Dicotómica Nominal	2006<br><br>Petti et al, 2008.
**Cambios ECG/Holter**				
**Cambios electro cardiográficos en ECG previamente normal.**	Alteraciones compatibles con cardiomiopatía Chagasica en ECG previamente normal.	Paciente con ecg normal que presenta cambios asociados a enfermedad de Chagas durante el seguimiento anual.<br>Si – No	Cualitativa Dicotómica Nominal	Rassi A Jr. et al, 2007
**Cambios electro cardiográficos nuevos.**	Nuevas alteraciones compatibles con cardiomiopatía Chagasica.	Paciente con ecg alterado para Chagas que evidencia nuevo alteración durante el seguimiento anual.<br>Si – No	Cualitativa Dicotómica Nominal	Bestetti, 2001
**Cambios en el Holter**	Nuevas alteraciones compatibles con cardiomiopatía Chagasica	Paciente que muestra cambios en Holter durante el seguimiento anual.<br>Si – No	Cualitativa Dicotómica Nominal	Lana et al, 2009.
**Taquicardia ventricular en Holter.**	Presencia nueva de TVS	Paciente presenta durante seguimiento anual nuevo hallazgo de Taquicardia Ventricular Sostenida o No Sostenida.<br>Si – No	Cualitativa Dicotómica Nominal	Barbosa et al, 2011
**Implantación de dispositivo cardiaco.**	Paciente con enfermedad de Chagas que requiere implantación de dispositivo cardiaco.	Paciente con implantación de dispositivo cardiaco que antes no estaba indicado y en post tratamiento es necesario.<br>Si – No	Cualitativa Dicotómica Nominal	Mady C & Nacruth R, 1995.
**Cambios Eco**				
**Anormalidades nuevas de la pared del VI**	Nuevas alteraciones en la movilidad de la pared del VI.	Nuevo aneurisma apical o alteración segmentaria de la movilidad del VI.<br>Si – No	Cualitativa Dicotómica Nominal	Fuentes et al, 2012.<br><br>Mann. 2011
**Cambios en la dimensión VI en sístole.**	Alteración de la dimensión del VI en sístole.	Incremento en la dimensión del VI.<br>Si - No	Cualitativa Dicotómica Nominal	
**Cambios dimensión VI al final de la diástole.**	Alteración de la dimensión del VI al final de la diástole.	Incremento DVID >5mm/línea de base evidenciado durante el seguimiento anual<br>SI - No	Cualitativa Dicotómica Nominal	
**Cambios en la**	Alteración en la FE	Disminución de la FE >5%	Cualitativa	

**Fracción de eyección FE.**		Si – No	Dicotómica Nominal	
**Necesidad de Trasplante cardiaco.**	Paciente con enfermedad de Chagas que requiere trasplante cardiaco.	Paciente con criterios para trasplante cardiaco que antes no estaba indicado y en post tratamiento es necesario.<br>Si – No	Cualitativa Dicotómica Nominal	
**Mortalidad**				
**Muerte súbita**	Muerte repentina de origen cardiaco.	Durante el seguimiento hay muerte súbita en el paciente con Chagas.<br>Si – No	Cualitativa Dicotómica Nominal	
**Muerte por falla cardiaca**	Muerte por descompensación progresiva de la función cardiaca.	Durante el seguimiento hay muerte por falla cardiaca en el paciente con Chagas.<br>Si – No	Cualitativa Dicotómica Nominal	
**Laboratorio**				
**PCR positividad post-tratamiento (Retrospectivo)**	Detección de ADN de *T.cruzi* en muestras de sangre de pacientes que han recibido tratamiento 1 a 11 años previos a la realización de prueba.	Positivo / Negativo	Cualitativa Dicotómica	Aguiar, et al,2012<br>Murcia et al.,2010<br>Fernandes et al.,2009<br>Braga et al., 2000<br>Lauria-Pires et al, 2000<br>Britto et al., 1999<br>Lana et al.,2009
**PCR Positividad (Prospectivo)**	Detección de ADN de *T.cruzi* en sangre en pacientes que han recibido tratamiento en muestras recolectadas antes del inicio y al final del tratamiento (60 días) y a los 6, 12 y 24 meses finalizado el tratamiento.	Positivo/ Negativo	Cualitativa Dicotómica	Marin Neto et al, 2009<br>Fernandes et al.,2009<br>Murcia et al.,2010
**PCR Carga parasitaria (Retrospectivo)**	Medición de la concentración de ADN de *T.cruzi*, equivalente al número de parásitos por mL en muestras de sangre	$0.0\text{-}1x\ 10^5$<br><br>Clasificación de carga parasitaria:<br><br>Baja >2 par/mL<br>Moderada < 10 par/mL	Cuantitativa Razón	Lauria-Pires et al, 2000<br>Braga et al., 2000

	de pacientes que han recibido tratamiento 1 a 11 años previos a la realización de prueba.	Alta > 10 Par/mL		
**PCR Carga parasitaria (Prospectivo)**	Medición de la concentración de ADN de *T.cruzi* obtenido de muestras de sangre de pacientes recolectadas, previa al inicio y al final del tratamiento (60 días) y a los 6, 12 y 24 meses finalizado el tratamiento.	0.0-1x 10^5<br><br>Clasificación de carga parasitaria:<br><br>Baja >2 par/mL<br>Moderada < 10 par/mL<br>Alta > 10 Par/mL	Cuantitativa Razón	Marin Neto et al., 2009
**Tesablot (Retrospectivo y Prospectivo)**	Presencia de anticuerpos IgG anti-T. cruzi en suero, detectados por Western blot utilizando TESA (antígenos de secreción y excreción obtenidos mediante cultivo de formas Tripomastigotes de T. cruzi). En muestras recolectadas al final del tratamiento (60 días) y a los 6, 12 y 24 meses después de finalizado el tratamiento.	Positivo/Negativo	Cualitativa Dicotómica Nominal	Machado de Asis et al., 2012<br>Bosseno et al., 2006
**ELISA Positividad**	Presencia de anticuerpos IgG anti-*T. cruzi* en suero unidos al antígeno, detectados mediante espectrofotometría en muestras de suero recolectadas minímo 3 años	Positivo/Negativo	Cualitativa Dicotómica Nominal	Fernandes et al.,2009<br>Viotti et al., 2011

	después de recibir tratamiento.			
**ELISA Absorbancia**	Medida espectrofotométrica de anticuerpos IgG-anti T.cruzi en función de su afinidad y concentración, en muestras de suero recolectadas minímo 3 años después de recibir tratamiento.	0.0-2.5	Cuantitativa Intervalo	Fernandes et al.,2009 Viotti et al., 2011
**HAI Positividad**	Presencia de anticuerpos IgG anti-T. cruzi en suero, detectados mediante aglutinación, en muestras de suero recolectadas minímo 3 años después de recibir tratamiento.	Reactivo/No reactivo	Cualitativa Dicotómica Nominal	Fernandes et al.,2009 Viotti et al., 2011
**IFI positividad**	Presencia de anticuerpos IgG anti-T. cruzi en suero, detectados mediante fluorescencia, en muestras de suero recolectadas minímo 3 años después de recibir tratamiento.	Reactivo/No reactivo	Cualitativa Dicotómica Nominal	Fernandes et al.,2009 Viotti et al., 2011
**IFI títulos**	Contenido de anticuerpos IgG anti-T.cruzi en diluciones seriadas del suero que se unen a una cantidad estándar de antígeno y determinan el nivel en el cual se produce la reacción positiva, detectada por fluorescencia, en muestras de suero recolectadas minímo 3 años	1/8, 1/16, 1/32, 1/64,1/128, 1/256, 1/512,1/1024, 1/2048	Cualitativa Ordinal	Fernandes et al.,2009 Viotti et al., 2011

	después de recibir tratamiento.			
**Genotipificación de *T.cruzi***	Determinación del contenido genómico para clasificación de *T.cruzi* en Unidades de Tipificación Discretas en ADN de *T.cruzi* obtenido de pacientes que han recibido tratamiento 1 a 11 años previos a la realización de prueba.	DTU´s:<br>TcI<br>TcII<br>TcIII<br>TcIV<br>TcV<br>TcVI	Cualitativa Politómica Nominal	Zingales, 2010 y 2012<br>Ramírez et al., 2010<br>Burgos et al.,2010
**Genotipificación de T.cruzi (Prospectivo)**	Determinación del contenido genómico para clasificación de T.cruzi en Unidades de Tipificación Discretas en ADN de *T.cruzi* de muestras obtenidas previas al inicio y al final del tratamiento (60 días) y a los 6, 12 y 24 meses finalizado el tratamiento.	DTU´s:<br>TcI<br>TcII<br>TcIII<br>TcIV<br>TcV<br>TcVI	Cualitativa Politómica Nominal	Zingales, 2010 y 2012<br>Ramírez et al., 2010<br>Burgos et al.,2010

Tipo de estudio y diseño general Objetivo 3

Cohorte prospectiva de pacientes con antecedente de tratamiento farmacológico por enfermedad de Chagas. Los pacientes registrados en la base de datos con este antecedente serán identificados por los investigadores de este estudio. Quienes cumplan con los criterios de inclusión y exclusión y previa obtención del consentimiento informado serán incluidos en esta fase del estudio, para ser interrogados, para toma de muestras hemáticas y exámenes cardiológicos.

Universo de estudio, selección y tamaño de muestra, unidad de análisis y observación. Criterios de inclusión y exclusión Objetivo 3.

Para el desarrollo de este objetivo se realizará convocatoria de los 400 pacientes tratados en el INS, se espera al menos lograr el 50% de la muestra (200 pacientes).

Criterios de Inclusión:

- Pacientes adultos que hayan recibido tratamiento tripanocida por diagnóstico de enfermedad de Chagas fase crónica.
- Pacientes con seguimiento post tratamiento que pertenezcan a la cohorte objeto de estudio.

Criterios de exclusión:

- Pacientes que no presenten información completa que permita el seguimiento (dirección, teléfono, etc.) en bases de datos.
- Pacientes que no tengan resultados de exámenes cardiacos previos (antes de iniciar tratamiento)

Procedimientos para la recolección de información, instrumentos a utilizar y métodos para el control y calidad de los datos Objetivo 3.

A los pacientes quienes cumplan con los criterios de Inclusión se les realizará al momento del registro y luego anualmente valoración clínica para determinar la clase funcional de acuerdo a la clasificación NYHA. Además, valoración con electrocardiograma/Holter y Ecocardiograma para determinar cambios en el sistema eléctrico y estructural del corazón, respectivamente con periodicidad anual. De esta manera se clasificaran de acuerdo a las escalas actuales que determinan la evolución de la cardiomiopatía chagasica, se usaran las clasificaciones AHA (American Heart Association), Consenso de Brasil, Andes modificada y Kuschnir. Todos los datos serán registrados en la historia clínica sistemática.

Se llevará a cabo toma de muestras de sangre en tubo seco y en tubos con EDTA, para realizar análisis serológicos (ELISA, IFI, HAI y TESA-Blot) y moleculares (PCR), respectivamente. La primera toma de muestra se realizará al momento de la inclusión luego a los seis meses y posteriormente cada año. (ver anexo 1 y 2)

La información será digitada en la historia clínica sistematizada. Se realizarán entrenamientos sucesivos a los investigadores en la aplicación de los formatos de recolección de la información y se realizarán pilotos para el ajuste y resolución de dudas específicas en las variables del documento. Se harán dos evaluaciones de la calidad de la información registrada en los formatos, por medio de verificación de una muestra aleatoria del total de los formatos registrados, correspondiente al 5% de las mismas. Errores superiores al 20% del total de variables registradas obligarán a la revisión del total de la información. Se realizarán entrenamientos sucesivos a los investigadores en la aplicación de los formatos de recolección de la información y se realizarán pilotos para el ajuste y resolución de dudas específicas en las variables del documento.

Dificultades y limitaciones del estudio Objetivo 3

La principal limitación de este estudio se relaciona con sesgo de selección por parte de los sujetos elegibles al mismo, quienes pueden no ser incluidos en el mismo debido al estatus vital potencialmente relacionado con la misma enfermedad de Chagas. Habría que limitar claramente el periodo transcurrido desde la ocurrencia de la enfermedad con el momento de esta entrevista para prever las pérdidas debidas a fallecimientos o a la no localización de los sujetos del estudio.
Inhibición de muestras: la adición de clorhidrato de guanidina a la muestra de sangre aunque es indispensable para su conservación y digestión proteica, eventualmente puede generar inhibición del ADN produciendo que en estas muestras no pueda realizarse la detección del parásito.

Plan de análisis de los resultados Objetivo 3

A partir de la revisión de la literatura científica se extrajeron variables que permitieran la medición, evaluación y clasificación de la tendencia de respuesta al tratamiento.
Se contemplaran tres aspectos: parasitológico, serológico, ecocardiográfico y eventualmente clínico, con estos, se diseñara un score para clasificar la tendencia de respuesta al tratamiento etiológico de la enfermedad de Chagas en fase crónica y de acuerdo a este se catalogarán como:

A. Grandes Respondedores
B. Medianos Respondedores
C. No respondedores

Se empleará el software Stata 10.0 (StataCorp. 2007. *Stata Statistical Software: Release 10.* College Station, TX: StataCorp LP.)

Métodos y modelos de análisis de los datos según tipo de variables Objetivo 3

Para evaluar el rendimiento operativo del score generado, se realizará una regresión logística multivariada con las variables incluidas en el score, con un número especifico de pacientes a partir de la cual, por medio de una curva ROC, se obtendrá el rendimiento operativo que dará información sobre validez y seguridad del mismo.
Una vez validado la escala se someterá a todos los pacientes incluidos en este objetivo con el fin de determinar la respuesta al tratamiento etiológico.

CONSIDERACIONES ETICAS GENERALES

Durante el desarrollo del estudio, se obtendrá de los sujetos participantes,

consentimiento informado de acuerdo a lo estipulado por el Centro Coordinador de Datos y Muestras (CCDM), una copia del mismo será entregada al paciente una vez se obtenga.
Para garantizar la confidencialidad de la información registrada en la historia clínica sistematizada, se generará una lista donde se codificará el registro de cada paciente. La codificación de los datos registrados en la base de datos limitará la individualización de la información registrada en la misma. Igualmente, los investigadores y digitadores firmarán tanto un acuerdo de confidencialidad, donde se comprometen a no divulgar los datos relacionados con el estudio. La base de datos será de acceso restringido.

PRESUPUESTO GLOBAL

		**Contrapartida**		
**Rubros**	**Financiado**	**Ejecutora(s)**	**Otras**	**Total**
COSTOS OPERATIVOS	1,000,000	1,500,000	0	2,500,000
EQUIPOS	151,740,000	66,000,000	0	217,740,000
EVENTOS ACADÉMICOS	4,000,000	2,000,000	0	6,000,000
GASTOS DE VIAJES	10,000,000	10,000,000	0	20,000,000
MATERIALES	85,359,600	40,000,000	0	125,359,600
PRESTACIÓN DE SERVICIOS TÉCNICOS	2,000,000	0	0	2,000,000
PUBLICACIONES Y PATENTES	5,000,000	0	0	5,000,000
RECURSO HUMANO CIENTÍFICO Y DE APOYO	249,678,587	40,000,000	60,000,000	349,678,587
**Totales**	508,778,187	159500000	60000000	728,278,187

JUSTIFICACION DEL PRESUPUESTO.

Para cumplir a cabalidad con los objetivos mencionados, se requiere un equipo de recurso humano el cual consta profesionales en Medicina, Bacteriología y un auxiliar técnico en Enfermería, financiados por

Colciencias, quienes se encargarán de llevar a cabo la caracterización de la cohorte de pacientes, su tratamiento etiológico, efectos adversos y respuesta terapéutica. Se realizarán viajes a los departamentos de Casanare y Boyacá, con el fin de cumplir con los objetivos trazados en pacientes presentes en dichos departamentos.

Se contará con una asesoría en Epidemiología para realizar los análisis de la información recolectada y se participará en un curso y una capacitación en técnicas moleculares de diagnóstico y genotipificación de T.cruzi.

Se adquirirán equipos médicos (ecocardiógrafo, electrocardiógrafo, tensiómetro, fonendoscopio, balanza, entre otros), además material tipográfico (formatos solicitud de exámenes, recetarios, etc) que permitirán la atención y valoración cardiológica de los pacientes.

Se comprarán equipos de Biología molecular (Robot para extracción de ADN y Nanodrop) para extracción y cuantificación de ADN, diagnóstico molecular, determinación de carga parasitaria y genotipificación de T.cruzi. Adicionalmente, se comprarán reactivos (Kits, iniciadores, sondas, dNTPs, entre otros) necesarios para ejecutar pruebas moleculares (PCR-RT) y pruebas serológicas (ELISA, HAI, IFI y TESA-blot).

Finalmente, se destinará presupuesto para llevar a cabo la divulgación de los resultados de la caracterización en dos eventos científicos nacionales, aun no definidos, dos publicaciones en revistas indexadas, aún no definidas. En cada uno de los eventos científicos participarán 2 personas.

PRODUCTOS ESPERADOS (Entregables y fechas de entrega)

RESULTADO/PRODUCTO ESPERADO	FECHA DE ENTREGA DEL PRODUCTO	ENTREGABLE
Estudiante de maestría en ciencias biológicas o salud pública.	Mes 36 de 36	Certificación de la Institución Educativa
Estudiante de medicina encargado de transcripción de historias clínicas.	Mes 36 de 36	Certificación de la Institución Educativa
Articulo en revista acerca de la utilidad de métodos moleculares en el diagnóstico de la enfermedad de Chagas.	Mes 30 de 36	Carta ó correo electrónico de aceptación de la publicación en una Revista Indexada
Artículo acerca de caracterización de cohorte de pacientes su tratamiento etiológico, efectos adversos y respuesta terapéutica.	Mes 30 de 36	Carta ó correo electrónico de aceptación de la publicación en una Revista Indexada

Ponencia acerca de los hallazgos sobre respuesta al tratamiento etiológico para enfermedad de Chagas	Mes 12 de 36	Constancia de presentación de resultados
Ponencia acerca de los resultados en cuanto a caracterización clínica y RAM	Mes 24 de 36	Constancia de presentación de resultados
Estandarización de historia clínica unificada y sistematizada	Mes 6 de 36	Historia clínica unificada y sistematizada
Caracterización cohorte de pacientes	Mes 18 de 36	Informe detallado de caracterización de pacientes
Estandarización de pruebas moleculares	Mes 18 de 36	Protocolo de estandarización de pruebas moleculares
Evaluación postratamiento	Mes 24 de 36	Informe detallado de resultados y análisis de variables que permiten realizar evaluación postratamiento

CRONOGRAMA DEL PROTOCOLO (Actividades con fechas estimadas).

**Actividad**	**MM/AAAA**
Revisión bibliográfica	Continua
Desarrollo y aprobación avales institucionales científicos y éticos para la ejecución del protocolo	11/2012
Preparación de infraestructura	12/2012
Organización de historias clínicas	12/2012
Selección de pacientes	12/2012
Recolección de datos y muestras	01/2013
Análisis de pruebas de laboratorio	02/2013
Análisis de datos	06/2013
Informes preliminares	07/2013
Presentación en evento científico	2013 – 2014
Sometimiento de manuscrito revista indexada	2014
Informe final	2014

REFERENCIAS BIBLIOGRÁFICAS.

Acquatella H. Echocardiography in Chagas Heart Disease. Circulation. 2007;115:1124-113.

Aguiar C.; Batista AM.; Pavan TBS.; Almeida EA.; Guariento ME..; Wanderley J.S.et. alSerological profiles and evaluation of parasitaemia by PCR and blood culture in individuals chronically infected by Trypanosoma cruzi treated with benzonidazole. Trop Med Int Health 2012; 17:3 (368-373)

Alarcón González R.; Caballero Castellanos H.; Navaza Buzón D.; Favier Torres MA.; Yais Elcea AM. Caracterización de pacientes con Enfermedad de Chagas. Municipio Guanare, Estado Portuguesa. Enero a Julio de 2010.III Congreso Regional de Medicina Familiar Wonca Iberoamericana – CIMF y X Seminario Internacional de Atención Primaria de Salud. Versión virtual; 2010 Mar 12-16; La Habana, Cuba. 2010

Itcheh J.; Moscatelli G.; Moroni S.; Garcia-Bournissen F.; Freilij H. Events After the Use of Benznidazole in Infants and Children With Chagas Disease. Pediatrics 2011; 127: 212-218

Andrade JP.; Marin Neto JA.; Paola A.; Vilas-Boas F.; Moraes Oliveira GM.; Bacal F.; Bocchi EA. I Latin American Guidelines for the Diagnosis and Treatment of Chagas' Heart Disease. Executive Summary. Arq Bras Cardiol 2011;96(6):434-442.

Avila Tang E.; Matsui E.; Wiesch DG.; Samet JM. Epidemiology of Asthma and Allergic Disease. In Adkinson NF. Middleton's Allergy: Principles and Practice, 7th ed.Maryland:Mosby; 2008. p. 715-756

Avila HA.; Sigman DS.; Cohen LM.; Millikan RC.; Simpson L. Polymerase chain reaction amplification of Trypanosoma cruzi kinetoplast minicircle DNA isolated from whole blood lysates: diagnosis of chronic Chagas' disease. Mol Biochem Parasitol. 1998;48(2):211-21

Barbosa MM.; Nunes MCP. Estratificación del riesgo en la enfermedad de Chagas. Rev Esp Cardiol. 2012;65(Supl 2):17-21

Basquiera AL.;Sembaj A.;Aguerri AM.;OmelianiukM.;GuzmánS.;Barral JM.;Caeiro TF.;Madoery RJ.; Salomone OA. Risk progression to chronic Chagas cardiomyopathy: influence of male sex and of parasitaemia detected by polymerase chain reaction. Heart 2003; 89:1186-1190.

Bern C. Antitrypanosomal Therapy for Chronic Chagas' Disease. N Eng J Med 2011.; 364:2527-34

Bestetti RB. Predictors of unfavourable prognosis in chronicChagas' disease. Trop. Med. Int. Health 2001;6(6):476-83.

Bosseno MF.; Bastrenta B.; Brenière SF.; Flores-Chavez M.; Dalenz J.; Revollo S.et. al. Polymerase chain reaction det.ection and serologic follow-up after treatment with benznidazole in Bolivian children infected with a natural mixture of Trypanosoma cruzi I and II. Am J Trop Med Hyg2006; 75:3 (497-501)

Braga MS.; Lauria-Pires L.; Argañaraz ER.; Nascimento RJ.; Teixeira ARL. Persistent infections in chronic chagas' disease patients treated with anti-Trypanosoma cruzinitroderivatives. Rev Inst Med Trop Sao Paulo 2000; 42:3 (157-161)

Brasil P.; Castro L.; Masslocher-Moreno A.; Sangenis L.; Braga JU. ELISA versus PCR for diagnosis of chronic Chagas disease: systematic review and meta-analysis. Brasil et al. BMC Infectious Diseases 2010, 10:337.

Britto C.; Cardoso MA.; Marques P.; Fernandes O.; Morel CM. Polymerase chain reaction detection: new insights into the diagnosis of chronic Chagas disease. Mem Inst Oswaldo Cruz. 1999;94 Suppl 1:305-6

Britto C.; Cardoso MA.; Ravel C.; Santoro A.; Pereira JB.; Coura JR. et al. Trypanosoma cruzi: parasite detection and strain discrimination in chronic chagasic patients from northeastern Brazil using PCR amplification of kinetoplast DNA and nonradioactive hybridization. Exp Parasitol. 1995 Dec;81(4):462-71.

Britto C.; Cardoso MA.; Wincker P.; Morel CM. A simple protocol for the physical cleavage of Trypanosoma cruzi kinetoplast DNA present in blood samples and its use in polymerase chain reaction (PCR)-based diagnosis of chronic Chagas disease.Mem Inst Oswaldo Cruz. 1993 Jan-Mar;88(1):171-2.

Britto C.; Cardoso A.; Silveira C.; Macedo V.; Fernandes O. Polymerase chain reaction (PCR) as a laboratory tool for the evaluation of the parasitological cure in Chagas disease after specific treatment. Medicina 1999; 59 Suppl 2 (176-178)

Burgos JM.; Diez M.; Vigliano C.; Bisio M.; Risso M.; Duffy T. et. al. Molecular identification of Trypanosoma cruzidiscret.e typing units in end-stage chronic Chagas heart disease and reactivation after heart transplantation. Clin Infect Dis. 2010; Sep 1;51(5):485-95.

Campbell-Lendrum DH.; Angulo VM.; Esteban L.; Tarazona Z.; Parra GJ.; Restrepo M. et al. House-level risk factors for triatomine infestation in Colombia. Int. J. Epidemiol2007; 36(4): 866-872.

Carrilero B.; Murcia L.; Martínez-Lage L. Segovia M. Side effects of benznidazole treatment in a cohort of patients with Chagas disease in non-endemic country. Rev Esp Quimioter 2011; 24: 123-6.

Chaves AM.; Villar JC.; Luengas CA.; Villamizar MC.; Hernández L.; Celis A. et. al. Función diastólica en sujetos con serología positiva paraenfermedad de Chagas procedentes del estudio CHICAMOCHA. Rev ColombCardiol 2006; 13: 79-84.

Chobanian AV.; Bakris GL.; Black HR.; Cushman WC.; Green LA.; Izzo JL. et. al. The Seventh Report of the Joint National Committee on Prevention, Detection, Evaluation andTreatment of High Blood Pressure and the National High Blood PressureEducation Program Coordinating Committee. JAMA 2003; 289: 2560-72.

Coelho H.L.; Arrais P.S.; Gomes A.P. Ceara State Pharmacovigilance System: a year of experience. Cad Saude Publica 1999; 15: 631–40.

Coronado X.; Ortiz S.; Lastra O.; Larrondo M.; Rozas M.; Solari A: Instability of Trypanosoma cruzi DNA in blood lysates: importance for PCR DNA-based diagnosis. Mol Diagn 2005, 9:35-40.

Coura J.R.; de Abreu L.L.; Willcox H.P.; Petana W. Comparative controlled study on the use of benznidazole, nifurtimox and placebo, in the chronic form of Chagas' disease, in a field area with interrupted transmission. Rev Soc Bras Med Trop. 1997; 30: 139-44.

Crema E.; Resende Silva EC.; Melo Franciscon P.; Rodrigues Júnior V.; Martins Júnior A.; Oliveira Teles CJ. et. al. Prevalence of cholelithiasis in patients with chagasic megaesophagus. Rev Soc Bras Med Trop 2011; 44(3): 324-326.

Crocco L.; Rodríguez C.; Catalá S.; Nattero J. Chagas disease in Argentina: tools for schoolchildren to exercise vector surveillance and identify household risk factors. Cad. Saúde Pública 2005; 21(2):646-651.

Crowther JR. ELISA: Theory and practice: Methods in molecular biology. Totowa.; New Jersey; 1995

Cucunuba ZM.; FlorezAC.; Cardenas A.; Pavıa P.; Montilla M.; Aldana R. et. al. Prevalence and Risk Factors for Chagas Disease in Pregnant Women in Casanare, Colombia. Am J Trop Med Hyg 2012; 87:837-842.

Cummings P. Effects of differences between peer reviewers suggested by authors and by editors. JAMA. 2006;296:1231.

Departamento Administrativo Nacional de Estadística (DANE). Cartilla de conceptos básicos e indicadores demográficos. Colombia: Dane; 2007.

Departamento administrativo nacional de estadística (DANE). Clasificación Internacional Uniforme de Ocupaciones Adaptada para Colombia CIUO-88 A. C. Colombia: DANE; 2005.

Departamento administrativo nacional de estadística (DANE). Colombia una nación multicultural, su diversidad étnica. Colombia: Dane; 2007.

Dorn PL.; Selgean S.; Guillot M: Simplified method for preservation and polymerase chain reaction-amplification of Trypanosoma cruzi DNA in human blood. Mem Inst Oswaldo Cruz 1997, 92:253-255.

Duffy T.; Bisio M.; Burgos J.M.; Levin M.J.; Schijman A.G.; Altcheh J.; Freilij H.; Diez M.; Favaloro R.R. Accurate real-time PCR strategy for monitoring bloodstream parasitic loads in chagas disease patients PLoS Neglected Tropical Diseases 2009 3:4

Fernandes CD.; Tiecher FM.; BalbinotMM.;Liarte DB.; Scholl D.; Steindel M.et.al. Efficacy of benznidazol treatment for asymptomatic chagasic patients from state of Rio Grande do Sul evaluated during a three years follow-up. Mem Inst Oswaldo Cruz 2009; 104:1 (27-32)

Fuentes R.; Maturana M.; de la Cruz R. Efi cacia de nifurtimox para el tratamiento de pacientes con enfermedad de Chagas crónica. Rev Chil Infect 2012; 29 (1): 82-86

Galvão L.; Chiari E.; Macedo AM.; Luquetti AO.; Silva SA.; Andrade A. et al PCR Assay for Monitoring Trypanosoma cruzi Parasitemia in Childhood after Specific Chemotherapy. Journal of Clinical Microbiology 2003 41:11

Garzon SA.; Lorga AM.; Nicolau JC. Electrocardiography in Chagas' heart disease São Paulo Med J. 1995; 113(2): 802-13.

Gilbert C. Academic press Dictionary of science and Technology.Editorial : Academic press; 1992.

Godlee F. Publishing study protocols: making them visible will improve registration, reporting and recruitment. BMC News and Views. 2001;2:4.

Gómez EA.; Senior JM.; Vélez S.; Navarrete S.; Sánchez DF.; Roa NL. et. al. Guías colombianas sobre la evaluación y el manejo de la falla cardíaca crónica del adulto. Rev Col Cardiol 2007;14(supl2):13-50.

Greenberg B.; Kahn AM. Clinical Assessment of Heart Failure. In: Bonow RO. Braunwald's Heart Disease - A Textbook of Cardiovascular Medicine, 9th ed. St. Louis: Elsevier; 2011. p. 505-516.

Grupi CJ.; Moffa PJ.;Barbosa SA., Sanches PC.; Barragan Filho EG.; V. Bellotti VM. Holter monitoring in Chagas' heart disease. São Paulo Med J 1995; 113(2): 835-40.

Guhl F.; Nichols R.S.; Montoya R.; Rosas F.; Velasco V.M.; Mora E. et. al. Rápida negativización serológica después del tratamiento etiológico para enfermedad de Chagas en un grupo de escolares colombianos. Curso de Diagnóstico, Manejo y Tratamiento de la enfermedad de Chagas OPS/MSF/SSA 2006; 205-212.

Guyatt G. Preparing a research protocol to improve chances for success. J Clin Epidemiol. 2006;59:893-9.

Hidron AI.; Gilman RH.;Justiniano J.;Blackstock AJ.;LaFuente C.; et al. (2010) Chagas Cardiomyopathy in the Context of the Chronic Disease Transition. PLoSNeglTropDis 4(5): e688

Higuchi R.;Fokler C.;Dollinger G.; Watson R. Kinetic PCR analysis: Real-time monitoring of DNA amplification reactions. Bio/Technology 1993;11:1026-30.

Hunt SA.; Abraham WT.; Chin MH.; Feldman AM.; Francis GS.; Ganiats TG. et. al. ACC/AHA 2005 Guideline Update for the Diagnosis and Management of Chronic Heart Failure in the Adult. Circulation. 2005;112:154-235.

Ianni BM.; Mady C.; Arteaga E.; Fernandes F. Doenças Cardiovasculares Observadas durante o Seguimento de um Grupo de Pacientes na Forma Indeterminada daDoença de Chagas. Arq Bras Cardiol 1998; 71(1): 21-24

Isselbacher EM.; Kligerman SJ.; Lam KM.; Hurtado RM. A 47-Year-Old Man with Abdominal and Flank Pain [case records]. N Engl J Med 2010;362:254-62.

Lana M.; Lopes LA.; Martins HR.; Bahia MT.; Machado-de-Assis GF.; Wendling Ap. et. al. Clinical and laboratory status of patients with chronic Chagas disease living in a vector-controlled area in Minas Gerais, Brazil, before and nine years after aetiological treatment. Mem Inst Oswaldo Cruz 2009;104(8): 1139-1147.

Lauria-Pires L.; Braga MS.; Vexenat AC.; Nitz N.; Simões-Barbosa A.; Tinoco DL. et. al. Progressive chronic chagas heart disease ten years after Treatment with anti-trypanosoma cruzi nitroderivatives. Am. J. Trop. Med. Hyg 2000;63(3, 4):111–118.

Machado-de-Assis GF.; Silva AR.; Do Bem VA.; BahiaMT.; Martins-Filho OA.; Dias J.et. al. Posttherapeutic cure criteria in Chagas' disease: conventional serology followed by supplementary serological.; parasitological.; and molecular tests. Clin Vaccine Immunol 2012; Aug;19(8):1283-91.

Mady C.; Nacruth R. Natural history of chronic Chagas' heart disease: prognosis factors. São Paulo Med J. 1995; 113(2): 791-796.

Mann DL. Management of Heart Failure Patients with Reduced Ejection Fraction Electrocardiography. In: Bonow RO. Braunwald's Heart Disease - A Textbook of Cardiovascular Medicine, 9th ed. St. Louis: Elsevier; 2011. p. 543 - 577

Marin-Neto JA.; Rassi A Jr.; Avezum A Jr.; Mattos AC.; Rassi A.; Morillo CA. et. al. The BENEFIT trial: testing the hypothesis that trypanocidal therapy is beneficial for patients with chronic Chagas heart disease. MemInst Oswaldo Cruz. 2009;104(Suppl 1):319-24.

Matsuda NM.; Miller SM.; Évora PRB.The chronic gastrointestinal manifestations of chagas disease.Clinics 2009; 64(12): 1219-24.

Ministerio da Saude. Secretaria de Vigilancia em Saude. Brazilian Consensus on Chagas disease. Rev Soc Bras Med Trop 2005;38 (suppl 3): 7–29

Ministerio de la Protección Social MPS, República de Colombia. Guía de Atención Clínica de la enfermedad de Chagas 2010.

Mirvis DM.; Goldberger AL. Electrocardiography. In: Bonow RO. Braunwald's Heart Disease - A Textbook of Cardiovascular Medicine, 9th ed. St. Louis: Elsevier; 2011. p. 126-167

Mora G.; Echeverry MC.; Rey GE.; López MC.; Posada LM.; Rivas FA. Frecuencia de anticuerpos anti-Trypanosoma cruzi en pacientes portadores de marcapasos de la Clínica San Pedro Claver de Bogotá. Biomédica 2007;27:483-9.

Moreira OC.;Ramírez JD.; Velázquez E.;Melo MF.; Lima-Ferreira C.;Guhl F.; Sosa-Estani S.; Marin-Neto JA.;Morillo CA.;BrittoC.Towards the establishment of a consensus real-time qPCR to monitor Trypanosoma cruziparasitemia in patients with chronic Chagas disease cardiomyopathy: A substudy from the BENEFIT trial.Acta Trop. 2013 Jan;125(1):23-31.

Murcia L.; Carrilero B.; Muñoz MJ.; Iborra MA.; Segovia M. Usefulness of PCR for monitoring benznidazole response in patients with chronic Chagas' disease: A prospective study in a non-disease-endemic country. J AntimicrobChemother 2010; 65:8 (1759-1764)

Nakazawa M.; Rosa DS.; Pereira VR.;Moura MO.; Furtado VC.; Souza WV.; Barros MN.;Abath FG.; Gomes YM.Excretory-secretory antigens of Trypanosoma cruzi are potentially useful for serodiagnosis of chronic Chagas' disease. ClinDiagn Lab Immunol. 2001 Sep;8(5):1024-7.

Olgin J.; Zipes DP. Specific Arrhythmias: Diagnosis and Treatment. In: Bonow RO. Braunwald's Heart Disease - A Textbook of Cardiovascular Medicine, 9th ed. St. Louis: Elsevier; 2011. p. 781-824

Peinado R. Presíncope: ¿un síntoma con igual significado pronóstico que el síncope? [editorial]. Rev Esp Cardiol 2004;57(7):613-6

Perez-ayala A.; Perez-molina JA.; Norman F.; Monge-maillo B.; Faro MV.; Lopez-velez R. Gastro-intestinal Chagas disease in migrants to Spain: prevalence and methods for early diagnosis. Ann. Trop. Med. Parasitol 2011; 105(1): 25-9

Petti M.; Viotti R.; Armenti A.; Bertocchi G.; Lococo B.; Álvarez MG. et. al. Predictores de insuficiencia cardiaca en la miocardiopatíachagásica crónica con disfunción asintomática del ventrículo izquierdo. Rev Esp Cardiol. 2008;61(2):116-22

Pinazo M.J.; Muñoz J.; Posada E.; López-Chejade P.; Gállego M.; Ayala E. et. al. Tolerance of benznidazole in treatment of Chagas' disease in adults. Antimicrob Agents Chemother 2010; 54: 4896-9

Prata A. Clinical and epidemiological aspects of Chagas disease[review].Lancet Infect. Dis 2000; 1: 92–100

Ramírez JD.; Guhl F.; Rendón LM.; Rosas F.; Marin-Net.o JA. Chagas Cardiomyopathy Manifestations and Trypanosoma cruzi Genotypes Circulating in Chronic Chagasic Patients. PLoSNeglTropDis 2010; 4 (11).

Ramírez JD.;Guhl F.;Umezawa ES.;Morillo CA.; Rosas F.; Marin-Neto JA.;Restrepo S. Evaluation of adult chronic Chagas' heart disease diagnosis by molecular and serological methods. J ClinMicrobiol. 2009 Dec; 47(12):3945-51.

Rassi A Jr.; Rassi A.; Little WC.; Xavier SS.; Rassi SG.; Rassi AG. et. al. Development and Validation of a Risk Score for Predicting Death in Chagas' Heart Disease. N Engl J Med 2006;355:799-808.

Rassi A Jr.; Rassi A.; Rassi SG. Studies Predictors of Mortality in Chronic Chagas Disease: A Systematic Review of Observational. Circulation. 2007;115:1101-1108

Rassi A.; Rezende JM. Estudo clínico-radiológico do esôfago e dos cólons na fase aguda da doença de Chagas com relato de três casos de remissão espontânea de aperistalse do esôfago do grupo I. Rev. Soc. Bras. Med. Trop 2011; 44(1):70-75.

Roitt I. Roitt Inmunología. Buenos aires: Argentina. Editorial Médica Panamericana : 2008

Rosas F.; Guhl F.; Velasco V.; Jumbo L.; Jaramillo C.; Rodriguez D. et. al. Morbilidad de la enfermedad de Chagas en fase crónica en Colombia. Detección de pacientes chagásicos con cardiopatía en un área endémica del departamento de Boyacá. Rev Col Cardiol 2002; 9: 349-359.

Sackett DL. On the determinants of academic success as a clinician scientist. Clin Invest Med 2001;24(2):94e100.

Salles G.; Xavier S.; Sousa A.; Hasslocher-Moreno A.; Cardoso C. Prognostic Value of QT Interval Parameters for Mortality Risk Stratification in Chagas' Disease: Results of a Long-Term Follow-Up Study. Circulation. 2003;108:305-312.

Secretaria de salud de México D.F. Guía para la Elaboración de Protocolos de Investigación Operativa en Calidad. Dirección General de Calidad y Educación en

salud. Dirección de Planeación e Innovación para la calidad. Subdirección de investigación en calidad. Abril 2002.

Solari A.; Ortíz S.; Soto A.; Arancibia C.; Campillay R.; Contreras M. et al. Treatment of Trypanosoma cruzi-infected children with nifurtimox: A 3 year follow-up by PCR Journal of Antimicrobial Chemotherapy 2001 48:4.

Storch W. Immunofluorescence in clinical inmunology: a primer and atlas. Berlín: AlemaniaEdicionesBirkhauser; 2000.

Suárez Pérez EL.; Pérez Cardona CM. Basic components in the development of research proposals in health sciences. P R Health Sci J. 1999; 18 Suppl A:,1-49.

The WHO Adverse Reaction Terminology – WHO ART. Terminology for coding clinical information in relation to drug therapy 2005.

Umezawa ES.;Nascimento MS.;Kesper NJ.;Coura.;JR.; Borges-Pereira.; J.; Junqueira.; ACV.; et al. Immunoblot Assay Using Excreted-Secreted antigens of Trypanossomacruzi in Serodiagnosis of Congenital.; Acute and Chronic Chagas' Disease. Journal of Clinical Microbiology.; 34(9): 2143-7.; 1996.

Valencia C.N.; Mancilla M.; Ramos D.; Zulantay L.; Molina M.; Torres A. et al. Tratamiento de la enfermedad de Chagas crónica en Chile. Efectos adversos de Nifurtimox. Rev. Ibero-Latinoam. Parasitol 2012;71: 97-108.

Venkatesan P.; Wakelin D. ELISAs for parasitologists: or lies.; damned lies and ELISAs. Parasitol Today. 1993;9:229-32

Villar JC. Desenlaces clínicos de sujetos con infección crónica por Trypanosoma cruzi tratados o no con agentestripanocidas. Un metaanálisis de estudios observacionales. MEDUNAB 2002; 5(15): 166-73.

Villar JC.; Congreso Medicina Tropical, Medellin, 2009

Viotti R.; Vigliano C.; Alvarez MG.; Lococo B.; Pet.ti M.Impact of Aet.iological Treatment on Conventional and Multiplex Serology in Chronic Chagas Disease. PLoSNegl Trop Dis 2012; 5(9): e1314.

Viotti R.; Vigliano C.; Lococo B.; Bertocchi G.; Petti M.;, Alvarez MA. et. al. Long-Term Cardiac Outcomes of Treating Chronic Chagas Disease with Benznidazole versus No Treatment. A Nonrandomized Trial. Ann Intern Med. 2006;144:724-734.

Viotti RJ.; Vigliano C.; Laucella S.; Lococo B.; Petti M.; Bertocchi G. et. al. Value of echocardiography for diagnosis and prognosis ofchronic Chagas disease cardiomyopathy without heartfailure. Heart 2004;90:655–660.

World Health Organization WHO. Comités de fármacoterapia - Guía práctica. 2003.

Yun O.; Lima M.A.; Ellman T.; Chambi W.; Castillo S.; Flevaud L. et. al. Feasibility, Drug Safety, and Effectiveness of Etiological Treatment Programs for Chagas Disease in Honduras, Guatemala, and Bolivia: 10-Year Experience of Médecins Sans Frontières. PLoS Negl Trop Dis 2009; 3: e488.

Zingales B.; Andrade SG.; Briones MRS.; Campbell DA.;Chiari E.;Fernandes O.; et al. (2009). A new consensus for Trypanosoma cruzi intraspecific nomenclature: second revision meeting recommends TcI to TcVI. Mem. Inst. Oswaldo Cruz 104: 1051–1054.

Zingales B.; Miles MA.; Campbell DA.;Tibayrenc M.;Macedo AM.; Teixeira MM.; et al. (2012) The revised Trypanosoma cruzisubspecific nomenclature: Rationale.; epidemiological relevance and research applications. Infect Genet Evol 12: 240-253.

ANEXO 1.

MÉTODOS SEROLOGICOS.
PROTOCOLO PARA TOMA, IDENTIFICACIÓN, CONSERVACIÓN y PROCESAMIENTO DE MUESTRAS PARA PRUEBAS SEROLOGICAS DE ENFERMEDAD DE CHAGAS

1. Obtención de muestras de suero para pruebas parasitológicas

- Por punción venosa tomar 5 ml de sangre sin anticoagulante.
- Dejar retraer el coágulo a temperatura ambiente por 30 minutos.
- Centrifugar a 1.500 r.p.m. durante 10 minutos.
- Separar el suero en tubo o en un vial con tapa.
- Identificar el vial con los datos del paciente y el código correspondiente.

2. Obtención de antígeno de *T. cruzi* para IFI:

Para obtener el antígeno de *T.cruzi* es necesario ejecutar el protocolo descrito a continuación:

- Tomar cepas de *T.cruzi* (epimastigotes) puras, obtenidas de cultivo en fase logarítmica de crecimiento.
- Filtrar el cultivo, en condiciones de completa asepsia utilizando gasa estéril.
- Centrifugar el sobrenadante producto de la filtración del cultivo, en tubo de polipropileno graduado de 50 mL, por 20 minutos a 3.000 ± 500 r.p.m., descartar el sobrenadante con pipeta pasteur y lavar 4 veces con PBS pH 7.2+0.4 estéril o solución salina estéril al 0.85%. El sobrenadante del primer lavado debe centrifugarse y lavar con PBS pH 7.2+0.4 estéril o solución salina estéril al 0.85%.
- Posterior a el último lavado, resuspender el sedimento en Formol 1% y dejar 18 horas a 4 ±2°C
- Centrifugar 20 minutos a 3.000 ± 500 r.p.m .y lavar sedimento 3 veces con PBS pH 7.2+0.4 estéril o solución salina estéril al 0.85%.
- Llenar el tubo graduado que contiene el sedimento con el antígeno con aproximadamente 45 ml de PBS pH 7.2+0.4 estéril o solución salina estéril al 0.85%, homogenizar perfectamente en agitador de Mazini 50±5 rpm y proceder a realizar el recuento de parásitos contenido en la alícuota, colocando una gota de la suspensión (10uL) en la lámina fluorescencia de 18 pozos y observar en el Microscopio óptico con objetivo de 40X.
- Buscar que la concentración de parásitos en la suspensión final del antígeno contenga un promedio de 20 a 30 parásitos por campo en objetivo de 40X. Para diluir con PBS pH 7.2+0.4 estéril o solución salina estéril tanto como sea necesario, para obtener la concentración anteriormente mencionada.
- Distribuir la suspensión de antígeno convenientemente diluido, colocando aproximadamente 0.01 mL ±0.001 mL (10±1 µl) en cada pozo de la lámina para

IFI. Durante todo el proceso de dispensación la suspensión antigénica debe agitarse constantemente para evitar la sedimentación de los parásitos y distribuir con jeringa de insulina que tenga aguja recortada o con multirepetidora que proporcione el volumen mencionado, a razón de una gota (10±1µL) por círculo.

• Secar las láminas por 15 minutos a temperatura ambiente y luego colocarlas en Incubadora a 37±1°C por 30 minutos

• Retirar de la incubadora, dejar a temperatura ambiente por 10 minutos y empacar en paquetes de cinco (5) láminas en papel milano, teniendo cuidado al manipular la superficie de la lámina que contiene el antígeno.

• Almacenar las láminas de antígeno en bolsas de cierre hermético, que contengan silica gel para evitar la humedad, marcadas en su parte externa con un rótulo que contenga: la identificación del antígeno, fecha, número de lote y cantidad de láminas contenidas en la bolsa.

• Colocar las bolsas en un recipiente de cierre hermético que es sellado con cinta de enmascarar y también identificado con los datos antes mencionados, los cuales son almacenados en el Revco a -70º C, 30±2º C o en el cuarto frío -18±2°C.

3. Obtención de antígeno De *T. cruzi* para ELISA

- Tomar cepas de *T.cruzi* (epimastigotes) puras, obtenidas de cultivo en fase logarítmica de crecimiento.
- Concentrar el cultivo transvasándolo a tubos Falcón de 50 ml y centrifugar a 4°C, 3.000 r.p.m. durante 20 minutos.
- Descartar el sobrenadante de cada uno de los frascos con pipeteador, para conservar la mayor cantidad de parásitos.
- Lavar el sedimento tres (3) veces con Solución Salina al 0.85 % durante 20 minutos a 4°C y 3.000 r.p.m, tener en cuenta que se debe descartar el sobrenadante como en el paso anterior.
- Pesar en balanza analítica todos los tubos Falcón vacíos con tapa, que serán usados para liofilización.
- Agrupar el sedimento obtenido de los lavados de todos los frascos Falcón en uno solo ó en un número adecuado de frascos previamente pesados dependiendo de la cantidad de sedimento obtenido, adicionar a cada uno de ellos 2 ml de Solución salina al 0.85 % y llevarlo a congelación por 18 horas a -70°C.
- Liofilizar los sedimentos y recoger la totalidad del liofilizado, raspando las paredes del tubo con espátula y pesarlo. Obtener el peso final por diferencia del peso obtenido del tubo Falcón con el liofilizado menos el peso del tubo Falcón vacío.
- Reconstituir el sedimento con Solución Salina al 0,85% a 4°C con un volumen correspondiente a la décima parte. Es decir, el peso final en miligramos se divide entre 10.
- Seguidamente sonicar tres veces a 20 KHz durante 15 segundos, teniendo en cuenta de utilizar una cubeta de hielo para mantener la red de frío.

- Posteriormente para completar la fragmentación del antígeno llevar la preparación a nitrógeno líquido (-196º C) durante 9 veces, teniendo en cuenta de asegurar el tubo Falcón con una cuerda triple, de tal forma que el nitrógeno no la pueda romper. En cada uno de estos pasos dejar descongelar totalmente la preparación y luego si introducirla en la bombona de nitrógeno liquido.
- Finalmente distribuir en alícuotas de 150 ul, determinar su concentración de proteínas por el método de Bradford y marcar cada una de las alícuotas con la fecha de preparación y su concentración de proteínas.
- Almacenar los viales obtenidos a –20º C.

4. Prueba IFI para determinación de anticuerpos IgG anti-*T.cruzi*

- Rotular las microplacas de fondo en U con los números correspondientes a las muestras a procesar (8 muestras por microplaca).
- Agregar un volumen (20μl ó 25μl) de PBS pH 7.2 en todas las celdas de la microplaca.
- Agregar (20μl ó 25μl) de las muestras incluyendo inicialmente los sueros controles internos de referencia (+) y (-). Posteriormente agregar el mismo volumen de cada una de las muestras de suero a procesar.
- Mezclar completamente y preparar las diluciones seriadas al doble por transferencia de (20μl ó 25μl..) a cada pozo sucesivo. Descartar el volumen final (20μl ó 25μl..) de la última dilución. Obtendremos las diluciones (1/2, 1/4, 1/8, 1/16, 1/32, 1/64, 1/128, 1/256, 1/512, 1/1024, 1/2048…) de la siguiente forma:

- Diluciones

1/2

1/4

1/2048

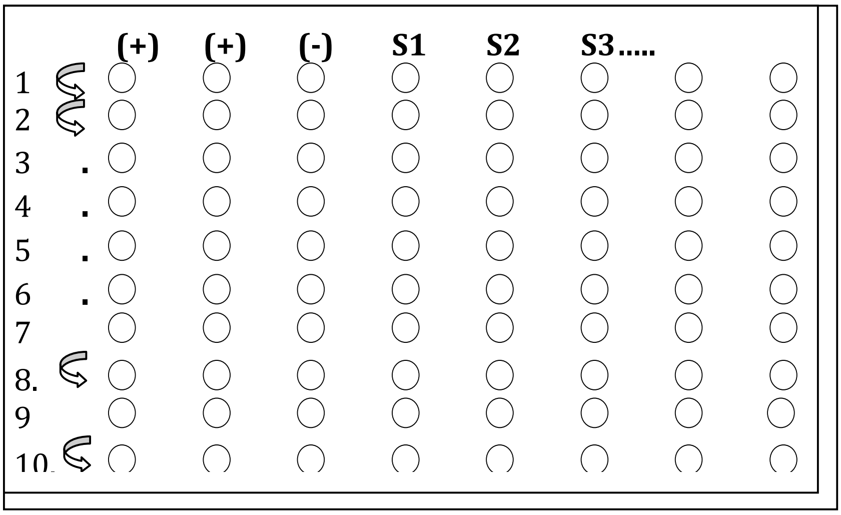

- Preparar las láminas portadoras del Ag hasta que alcancen la temperatura ambiente. Roturarlas con el número del código de las muestras.
- Dispensar 12 ul de cada una de las diluciones realizadas sobre los pozos de las láminas de Ag. Empezar de la más diluida (1/2048) a la más concentrada (1/8).

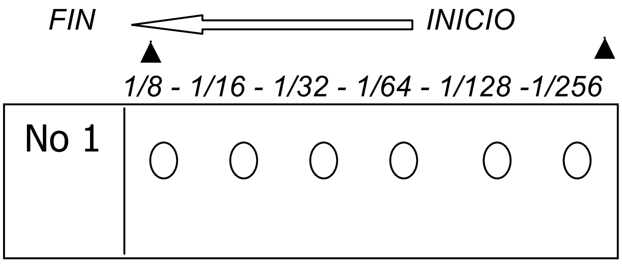

- Colocar las láminas en cámara húmeda a 37º C de 45 - 60 minutos en posición perfectamente horizontal evitando movimientos bruscos.
- Lavar cada lámina con PBS pH 7.2, utilizando un frasco lavador.
- Lavar las láminas en PBS pH 7.2 por 5 minutos por inmersión, utilizando cubetas de coloración y rotación suave. Este paso debe realizarse 2 veces.
- Dejar secar las láminas a temperatura ambiente o utilizando un ventilador suave. No utilizar calor
- Prepara el conjugado previamente titulado y cubrir las áreas circulares de las láminas con 12 ul de conjugado anti- IgG, diluido en Azul de Evans 1:1.000.
- Incubar nuevamente las láminas en cámara húmeda a 37º C por 45 - 60 minutos.
- Lavar cada lámina con PBS pH 7.2, utilizando un frasco lavador.
- Lavar las láminas en PBS pH 7.2 durante 5 minutos por inmersión, utilizando cubetas de coloración y rotación suave. Este paso debe realizarse 2 veces.
- Realizar un último lavado con agua destilada por 5 minutos por inmersión, utilizando cubetas de coloración y rotación suave.
- Secar las láminas con aire comprimido muy suavemente, con ventilador o dejarlas secar espontáneamente (no exponer a la luz para evitar pérdida de fluorescencia) y agregarles 1 gota de glicerina bufferada pH 9.0.

- Colocar laminillas de 22 x 60 mm para cubrir completamente cada uno de los pozos de la lámina.
- Observar al microscopio de fluorescencia con objetivo de 40X.
- **Lectura :** Siempre se debe montar la reacción con un suero control positivo, un suero control negativo y un control de PBS pH 7.2

  - **Reacción positiva:** Los parásitos se observan de color verde manzana brillante tanto el cuerpo como los flagelos.
  - **Reacción negativa:** Los parásitos se observan de color rojo ladrillo opaco, sin fluorescencia.
  - **Control de PBS pH 7.2**: Los parásitos se observan de color rojo.
- **Interpretación de la técnica:** Se considera como positivo para enfermedad de Chagas un título mayor o igual a 1/32.

5. Técnica ELISA para la determinación de anticuerpos IgG anti-*T cruzi*

Para realizar las pruebas de ELISA para diagnóstico de *T.cruzi,* es necesario llevar a cabo los siguientes procedimientos:

- **Sensibilización del antígeno.** Adicionar en cada uno de los pozos un volumen de 100μl de antígeno en concentración óptima, incubar por 3 horas en cámara húmeda a 4ºC. Posteriormente, realizar tres lavados en el lavador de microplacas, utilizando la tarjeta de 4 lavados x 12. De una u otra forma, los lavados se deben realizar con solución de trabajo PBS-Tween. 0.1 M pH 7,4 para eliminar restos del antígeno aplicado y evitar un cambio de pH en la reacción.
- **Antígeno de concentración antigénica óptima o de trabajo:** Realizar dilución en buffer carbonato 0.05 M pH 9.6, cuya función principal es estabilizar el antígeno en la dilución óptima de trabajo. Esta concentración para la técnica de Tripanosomiasis es de 7,5 μg/ml
- **Adición de la muestra.** Agregar 100 μl de la dilución óptima de cada una de las muestras de los pacientes en estudio y los respectivos controles conocidos. Dejar 2 horas de incubación en cámara húmeda a temperatura ambiente. Realizar los lavados de la placa de la misma forma como se describió en el numeral 1
- **Dilución óptima de la muestra:** Las muestras problemas deben ser diluidas de la 1:1000 (10 μl de muestra en 10 ml de buffer PBS-Tween). Cada muestra en su dilución respectiva debe procesarse por triplicado.

- **Adición del conjugado.** Adicionar 100 μl de la dilución óptima del conjugado anti - IgG ligado a fosfatasa alcalina en cada uno de los pozos. Incubar por 18 horas en cámara húmeda a 4 ºC y posteriormente, realizar tres lavados como se indicó antes.
- **Dilución óptima del conjugado (anti - IgG humana unido a fosfatasa alcalina).** El conjugado utilizado (anti - IgG humana unido a fosfatasa alcalina comercial) se debe titular previamente para obtener la dilución óptima de trabajo. Esta titulación se realiza con controles de referencia conocidos, teniendo en cuenta de utilizar títulos desde 1:1000 hasta 1:12000. Las diluciones se realizan en Buffer PBS-Tween.
- **Adición del sustrato cromogénico.** Adicionar 100 μl de la dilución del sustrato (Para-nitro-fenil-fosfato – Dietanolamina) e incubar en cámara húmeda por 30 minutos a temperatura ambiente.
- **Interrupción de la reacción.** Detener la reacción con 25 μl de hidróxido de sodio (NaOH) 3 M. El hidróxido de sodio también proporciona a la reacción inmunoenzimática un aumento de la intensidad y estabilidad del tono amarillo que le confiere el cromógeno a la reacción.

- **Lectura:** Realizar la lectura de la densidad óptica de cada una de las muestras, para lo cual se utiliza un fotocolorímetro Multiskan a una longitud de onda de 405 nm. La muestra restante luego del procesamiento de la misma será manejada así: 0±4ºC por 5 días a -18±2 °C (cuarto frío) temperatura donde se encuentra el banco de muestras

- **Preparación de Controles**

    - **Controles Internos:** Se utilizarán los siguientes controles internos:

- Blanco de reactivos: con cada lote de muestras deben analizarse un blanco de reactivos en los cuales la muestra es reemplazada por un volumen equivalente de conjugado. Este blanco debe ser procesado de igual forma que las muestras.
- Control de antígeno: con cada lote de muestras deben analizarse un control de antígeno en los cuales la muestra es reemplazada por un volumen equivalente de antígeno. Este blanco debe ser procesado de igual forma que las muestras.
- Control de muestras: para validar la prueba, con cada lote de muestras debe determinarse simultáneamente cuatro controles de referencia de muestras de suero de densidades ópticas conocidas (dos positivas y dos negativas), cuatro de líquido cefalorraquídeo (dos positivos y dos negativos), cuatro de papel filtro (dos positivas y dos negativas) o cuatro de humor vítreo (dos positivas y dos negativas), dependiendo del montaje que se vaya a realizar. El registro de los resultados de estos controles se lleva a cabo en la tabla de trazabilidad ubicada en el los cuaderno de trabajo correspondientes a cada patología, de acuerdo con las instrucciones del lomo.

- **Control Externo:** La exactitud de la prueba de ELISA para cisticercosis y tripanosomiasis se evalúa con el Control de Calidad Externo Nacional, con una frecuencia de dos evaluaciones anuales. Para IFI Chagas también se cuenta con un Control de Calidad Externo Internacional, también con una frecuencia de dos evaluaciones anuales, tal como se evidencia en los reportes.

- **Interpretación de los resultados**

Las densidades ópticas o absorbancias de muestras de suero menores al punto de corte (0,300) son consideradas como negativas y las absorbancias mayores o iguales a este punto, son consideradas como positivas.

6. Técnica HAI para la determinación de anticuerpos IgG anti-*T cruzi*

Se realizará la prueba de HAI mediante el uso del Kit Chagatest.(Wiener Lab)

7. Técnica TESA-BLOT para la determinación de anticuerpos IgG anti-*T cruzi*

Se realizará mediante el uso de la prueba comercial TesaBlot(Enfermedad de Chagas), comercializada por Biomeriux.

ANEXO 2.

METODOS MOLECULARES.

PROTOCOLO PARA TOMA DE MUESTRA, CONSERVACION Y PROCESAMIENTO DE PRUEBAS MOLECULARES PARA DETECCIÓN DE *Trypanosoma cruzi*

A. TOMA DE MUESTRA DE SANGRE

- Ubicar el paciente en una posición cómoda para la toma de muestra
- Marcar y rotular con los datos del paciente y el código correspondiente.
- Inspeccionar la vena que se va a puncionar.
- Realizar asepsia con alcohol sobre el sitio de la punción.
- Colocar el torniquete con suficiente tensión sin excederse
- Realizar la punción sobre la vena
- Tomar primero la muestra de sangre en tubo seco tapa amarilla.
- Tomar dos tubos con EDTA tapa lila de 10 ml.
- Indicar al paciente que debe hacer presión en el sitio punzado y colocar algodón sobre la herida de la punción.

Nota: Es indispensable tener en cuenta las normas de Bioseguridad y barreras de protección necesarias durante el proceso de toma de muestra.

B. CONSERVACION DE LA MUESTRA DE SANGRE PARA PCR

- Tomar los dos tubos tapa lila y mezclar la sangre en un tubo falcón de 50ml.
- Anadir volumen igual al obtenido de clorhidrato de guanidina 6M-EDTA 0.2M
- Mezclar durante 15 minutos por agitación suave
- Conservar las muestra a temperatura ambiente hasta la extracción de ADN.

Nota: Si la extracción de ADN es posterior a seis meses se debe refrigerar la muestra a 4°C.

C. PREPARACION DE CLORHIDRATO DE GUANIDINA

- Pesar 22,33 g de EDTA 0,2M.
- Agregar agua destilada hasta 300 ml.
- Agitar hasta disolver completamente.
- Agregar 10 perlas de hidróxido de sodio y 286,52 g de clorhidrato de guanidina 6M.
- Completar con agua destilada hasta 500 ml.
- Cuadrar pH a 8.0 con hidróxido de sodio al 40 %.
- Esterilizar a 15 libras de presión (121°C) durante 15 minutos
- Dejar enfriar y almacenar en nevera a 4°C

D. CONTROLES

1. CEPAS CONTROL

Utilizar las siguientes cepas control:

- **DTU TcI:** CG
- **DTU TcII:** PG98
- **DTU TcIII:** CM17
- **DTU TcIV:** CANIII
- **DTU TcV :** MN cl 2
- **DTU TcVI:** Cl Brener

2. CONTROL INTERNO DE AMPLIFICACIÓN

- Para mejorar la precisión de la cuantificación se incorporará al ensayo un plásmido recombinante lineal como estándar interno (IAC) que se agrega a la muestras de sangre original y se utiliza para normalizar el valor crudo de la carga parasitaria, según el rendimiento de la extracción de ADN de la muestra sanguínea y la eficiencia de amplificación (Duffy *et al.*, 2009).

3. CONTROLES DE EXTRACCIÓN Y DE REACCIÓN

- Al realizar la extracción de ADN se utilizará como control de extracción agua en lugar de muestra.

- Para todas las reacciones de PCR se utilizará como control negativo ADN obtenido de pacientes sanos, como control blanco se usará agua en lugar de ADN. Adicionalmente, se someterá a amplificación el control de extracción.

E. EXTRACCION DE ADN DE CONTROLES

1. Contaminar muestras de sangre en clorhidrato de guanidina de un paciente control sano con 10^6 parásitos (epimastigostes de las cepas utilizadas como control) y realizar diluciones seriadas, antes de la extracción de ADN.

2. Realizar extracción de ADN de cada una de las diluciones de sangre contaminada con parásitos mediante el kit comercial (High Pure PCR Template preparation Kit, Roche Applied Science) ó por extracción automatizada.

3. Realizar diluciones seriadas de las diluciones de ADN extraído en el paso 2.

4. Realizar extracción de de ADN de cepas control de *T.cruzi* obtenidas de cultivo en medio BHI en fase logarítmica de crecimiento.

F. EXTRACCIÓN DE ADN DE MUESTRAS

1. Realizar extracción de ADN de cada de sangre en clorhidrato de guanidina 6M mediante el kit comercial (High Pure PCR Template preparation Kit, Roche Applied Science) ó por extracción automatizada.

G. AMPLIFICACIÓN DE ADN MEDIANTE PCR-RT CON SONDAS Taq-Man

Los ensayos de PCR se realizarán en el termociclador ABI Prism 7500 Fast System y el análisis de los resultados se realizará mediante el software Software High Resolution Melt (HRM) v3.0. El procedimiento para la amplificación y cuantificación de ADN de *T.cruzi* se describe a continuación:

1. Para un volumen final de 20ul, realizar la mezcla de reacción añadiendo 10 ul de Master Mix Taqman, 2 ul de ADN y los iniciadores cruzi1/cruzi2 y la sonda cruzi 3, para amplificación de ADN nuclear y los iniciadores kDNA Fw/kDNA Rv y la sonda kDNA P, para amplificación de ADN de Kinetoplasto (Moreira et al., 2012). Los programas de ciclaje así como las secuencias se relacionan en la Tabla 1.

2. Añadir a la mezcla de reacción 2 ul de las diluciones de ADN extraído de las cepas control mencionadas previamente realizar curvas de amplificación desde 1 parasito/mL hasta 10^{5} parásitos/mL.

3. Añadir a la mezcla de reacción 2 ul de las diluciones de ADN extraído de la Cepa CG de TcI realizar curva estándar para cuantificación desde 10^{-2} a 10^{5} parásitos/mL.

4. Añadir a la mezcla de reacción 2 ul de ADN obtenido de las muestras de pacientes con enfermedad de Chagas y realizar detección y cuantificación de *T.cruzi.*

Primers	Secuencia	Programa de Ciclaje
cruzi 1 (Forward)	ASTCGGCTGATCGTTTTCGA	50 °C x 2 min.<br>95 °C x 10 min.<br><br>**40 ciclos :**<br>95 °C x 15 min.<br>58 °C x 2 min.
cruzi 2 (Reverse)	AATTCCTCCAAGCAGCGGATA	
cruzi 3 (Sonda)	FAM-CACACACTGGACACCAA-NFQ-MGB	
kDNA Fw (Forward)	GGGCGTTCAAATTTTG	50 °C x 2 min.<br>95 °C x 10 min.
kDNA	CAATCGAACCCCACCT	

Rv(Reverse)		**40 ciclos :**<br>95 °C x 15 min.<br>60 °C x 2 min.
kDNA P (Probe)	FAM-TCATGCATCTCCCCCGTACATTATTT-TAMRA	
IACTq	VIC-AGCATCTGTTCTTGAAGGT-NFQ-MGB	

H. GENOTIPIFICACIÓN DE *T.cruzi*

La Genotipificación en sus seis DTU, se realizará mediante los algoritmos propuestos por Ramírez et al., 2010 y Burgos et al., 2010. Utilizando como marcadores moleculares: SL-IR, 24Sa-rDNA y A10, cuyas secuencias de iniciadores se relacionan en la Tabla 2 y en la Figura 1..

Tabla 2. Iniciadores para genotipificación.

Marcador Molecular	Primers
SL-IR	**TCC**: CCC CCC TCC CAG GCC ACA CTG<br>**TC1**: GTG TCC GCC ACC TCC TTCGGG CC<br>**TC2** : CCT GCA GGC ACA CGT GTG TGT G
24Sa-rDNA	**D71**: 5-AAG GTG CGT CGA CAG TGT GG-3<br>**D72:** 5- TTT TCA GAA TGG CCG AAC AGT-3
A10	**Pr1:** CCGCTAAGCAGTTCTGTCCATA<br>**P6** GTGATCGCAGGAAACGTG

Figura 1. Algoritmo de marcadores moleculares para genotipificación (Modificado por Ramìrez JD, 2013, a partir de Ramírez et al., 2010).

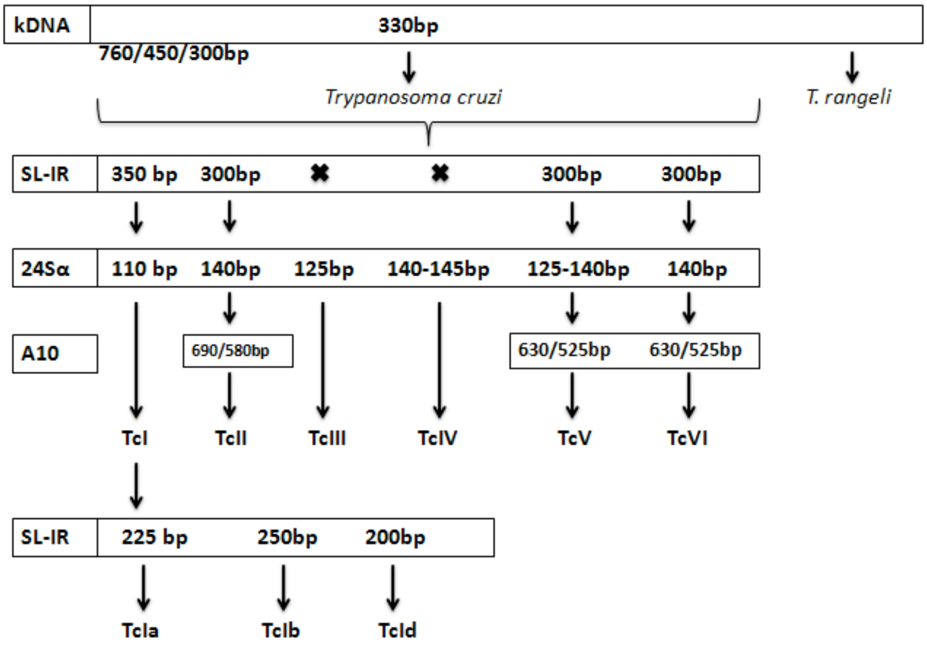

Supplement: S5 Appendix — Project: Characterization of a cohort of patients with Chagas disease, its etiological treatment, adverse events and therapeutic response. (PDF) [file pntd.0004997.s007.pdf]
